# β-sitosterol alleviates dextran sulfate sodium-induced experimental colitis via inhibition of NLRP3/Caspase-1/GSDMD-mediated pyroptosis

**DOI:** 10.3389/fphar.2023.1218477

**Published:** 2023-10-26

**Authors:** Di Zhang, Fei Ge, Jing Ji, Yu-Jing Li, Fu-Rong Zhang, Shu-Yan Wang, Shu-Jing Zhang, Dong-Mei Zhang, Meng Chen

**Affiliations:** ^1^ School of Traditional Chinese Medicine, Beijing University of Chinese Medicine, Beijing, China; ^2^ Key Laboratory of Chinese Internal Medicine of Ministry of Education and Beijing, Dongzhimen Hospital, Affiliated Hospital of Beijing University of Chinese Medicine, Beijing, China

**Keywords:** β-sitosterol, ulcerative colitis, inflammatory factors, Caspase-1, GSDMD, pyroptosis

## Abstract

**Background:** Inflammation-related NLRP3/Caspase-1/GSDMD-mediated pyroptosis is involved in the progression of ulcerative colitis (UC). β-sitosterol (SIT) was reported to have anti-inflammatory effects on experimental colitis, while the regulation of SIT on pyroptosis is unclear. Therefore, the present study aimed to define the protective and healing effects of SIT on dextran sulfate sodium (DSS)-induced experimental UC rats and human epithelial colorectal adenocarcinoma cells (Caco-2) and explore the underlying mechanisms that are responsible for its effects on NLRP3/Caspase-1/GSDMD-mediated pyroptosis in UC.

**Methods:** UC model rats were established by oral 4% DSS. Following colitis injury, the animals received SIT (doses of 50, 100, and 200 mg/kg) treatment for 2 weeks. For *in vitro* study, we exposed Caco-2–50 mg/mL DSS with or without SIT (concentrations of 8 and 16 μg/mL). Disease activity index (DAI) and histopathological injury were assessed *in vivo*. Activation proteins of nuclear factor kappa B (NF-κB) signaling axis, and tight junction-related proteins of zonula occludens-1 (ZO-1) and occludin were detected in colon tissues. TNF-α, IL-1β, and IL-18 in serum and cell supernatant were measured by enzyme-linked immunosorbent assay (ELISA). Changes in NLRP3/Caspase-1/GSDMD-mediated pyroptosis signaling pathway activation were analyzed both in tissues and cells.

**Results:** Our findings suggested that SIT treatment attenuated the severity of 4% DSS-induced UC by protecting rats from weight and colon length loss, and macroscopic damage. SIT also reduced proinflammatory factors production (TNF-α, IL-1β, and IL-18) in serum and cell supernatant. Mechanistically, SIT downregulated the expression levels of pyroptosis-related proteins including Caspase-1, cleaved-Caspase-1, NLRP3, GSDMD, and GSDMD-N in colon tissues and Caco-2 cells. Further analysis indicated that SIT maintained the colonic barrier integrity by enhancing the protein expression of ZO-1 and occludin.

**Conclusion:** We confirmed that SIT exerts protective and therapeutic effects on DSS-induced colitis injury by suppressing NLRP3/Caspase-1/GSDMD-mediated pyroptosis and inflammation response. These findings demonstrated that SIT could be a potential medication for UC treatment.

## 1 Introduction

Ulcerative colitis (UC), a global public health problem, which is a chronic non-specific, non-infectious, and inflammatory intestinal disease. It is a continuous mucosal ulcer with unknown origin and usually begins in the rectum, involves the colonic mucosa and submucosa layer, and then spreads to the cecum for the longest, which is characterized by continuous and diffuse distribution. Clinical manifestations of this illness are chronic and recurrent with abdominal pain, diarrhea, fecal bleeding, weight loss, and intestinal mucosal (Ordás et al., 2012). In recent years, UC has had increasing prevalence and incidence in developing and developed countries worldwide due to the rapid economic development and westernized diet uptake (Ng et al., 2017). Additionally, the incidence among people with UC in China has nearly tripled in the past decades (Ye et al., 2013). Research shows that patients with long-term UC have a higher risk of developing colorectal cancer and colitis-associated cancer is a major cause of death in patients with UC (Rogler, 2014), thereby resulting in a high economic burden on individuals and society health systems. For the above reasons, effective intervention in the course of UC is urgent.

Though the etiology of UC remains undefined, the inflammatory response definitely exists in the pathogenesis of UC (Du and Ha, 2020). Studies have suggested the large aggregation of inflammatory molecules such as TNF-α, IL-1β, and IL-18 could lead to colonic tissue injury and epithelial integrity disruption in the gut while inhibiting cytokine-mediated inflammation could treat or cure UC (Elmaksoud et al., 2021). NF-κB is a key transcriptional regulator in the regulation of proinflammatory mediators and chemokines production and secretion, which leads to an inflammatory cascade (Cui et al., 2019). The abnormal activation of NF-κB signaling was founded in colonic tissues of UC patients and experimental models (Neurath et al., 1996). Pyroptosis is a newly found form of pro-inflammatory programmed cell death unlike traditional necrosis and apoptosis (Shi et al., 2015). Pyroptosis plays an essential role in host defense and inflammatory responses, in which Caspase-1 or Caspase-11/4/5 was activated during this process (Yu P. et al., 2021). Activation of inflammatory caspases can trigger pyroptosis and release pro-inflammatory cytokines IL-1β and IL-18, which are relevant to inflammatory diseases (Yuan et al., 2018). In addition, over-expression of pyroptosis-related proteins, including nucleotide-binding oligomerization segment-like receptor family 3 (NLRP3), apoptosis-associated speck-like protein (ASC), Caspase-1, and gasdermin D-N-terminal (GSDMD-N) in colonic tissues has been observed in experimental colitis (Chao et al., 2020; Wei et al., 2021) and suppression of the NLRP3/Caspase-1/GSDMD-mediated pyroptosis signaling pathway can attenuate the damage of UC (Jie et al., 2021), thus therapy targeting pyroptosis could be promising and merits further investigation.

**FIGURE 1:**
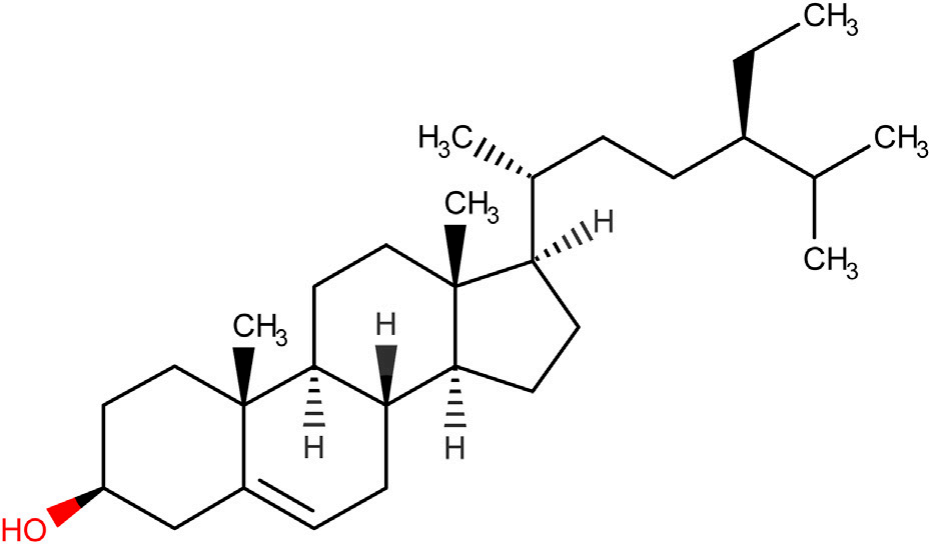
Chemical structure of β-sitosterol. (DrugBank Accession Number: DB14038).

While immunosuppressants, glucocorticoids, salicylic acid, and biological agents are the frontline therapy for clinical patients with UC (Nakamura et al., 2008; Berends et al., 2019), there are many side effects associated with these pharmaceutical medications (Moreau and Mas, 2015). Therefore, more researchers have paid attention to natural plant substances, which will be safe and beneficial with less deleterious effects (Cao et al., 2019). Phytosterols are a critical class of bioorganic compounds that are abundant in a range of organisms such as plants, animals, and fungi, and exert an important role in the physiological processes of eukaryotes (Zhang et al., 2022). β-sitosterol (SIT) (Figure 1), the major compound of phytosterol, is a plant-derived natural product widely present in many countries. Its pharmacological effects and biological activities were well documented in the literature including anti-inflammation (Loizou et al., 2010; Sun et al., 2020), anti-anxiety (Panayotis et al., 2021), antiviral properties (Chen et al., 2022), anti-oxidant stress, immunomodulation (Cheng et al., 2020), anti-diabetes (Babu et al., 2020), anti-tumor (Bae et al., 2021), anti-microbes (Pierre Luhata and Usuki, 2021), hepatoprotective effects (Kim et al., 2014), cardioprotective effects (Lin et al., 2020), anti-diabetes (Ponnulakshmi et al., 2019), as well as regulation of gut microbiota (Yu Y. et al., 2021). These studies showed that SIT could be a potential measure of various diseases. Furthermore, evidence from experimental studies on SIT indicates its anti-inflammatory ability and can be used as a safe pharmaceutical complement in the treatment of experimental colitis (Lee et al., 2012; Aldini et al., 2014; Bin Sayeed et al., 2016; Feng et al., 2017; Ding et al., 2019). Of note, SIT has been proven experimentally that it does not produce cytotoxic impacts under long-term use (Malini and Vanithakumari, 1990; Paniagua-Pérez et al., 2005; Feng et al., 2020). Despite this, further experimental studies are required to uncover the role of SIT in the recovery of experimental UC and elucidate its possible anti-pyroptosis mechanism behind this.

Thus, in this study, we established the DSS-induced experimental colitis in rats and Caco-2 cells to assess the protective and therapeutic effects of SIT on UC and further explore its underlying mechanism.

## 2 Materials and methods

### 2.1 Drug, and reagents

Dextran sulfate sodium and β-sitosterol (SIT, purity >95% and HPLC ≥98%) were purchased from Shanghai Yuanye Bio-Technology Co., Ltd. (Shanghai, China). Carboxymethyl cellulose was purchased from Biotopped Technology Co. Ltd. (Beijing, China). Enzyme-linked immunosorbent assay (ELISA) kits of TNF-α, IL-1β, and IL-18 were obtained from Cloud-Clone Corp (Wuhan, China). Antibodies against NLRP3, occludin, β-Actin, goat-anti-rabbit IgG H&L (HRP), and goat-anti-mouse IgG H&L (HRP) were purchased from Abcam (Cambridge, United Kingdom). Antibodies against Caspase-1, GSDMD, IKB alpha, and ZO-1 were obtained from Proteintech (Wuhan, China). Antibodies against NF-kappaB p65, phospho-NF-kappaB p65, and phospho-IkB alpha were purchased from Invitrogen (Carlsbad, CA). Antibody against cleaved Caspase-1 was purchased from Cell Signalling Technology (Danvers, MA, United States). Antibody against GSDMD-N terminal was purchased from ABclonal (Wuhan, China). The Cell Counting Kit-8 (CCK-8) was purchased from Biorigin (Beijing, China).

The following reagents were obtained from GenePool Biotechnology (Beijing, China): Total RNA Extraction with DNase Ⅰ Kit, mRNA cDNA Synthesis Kit, mRNA qPCR Kit, RNA Loading Buffer, BSA Blocking Buffer, SDS-PAGE Gel Ki, SDS-PAGE Loading Buffer, Tris-Glycine Running Buffer, WB Transfer Buffer, and TBST. The following reagents were purchased from Beyotime Biotechnology (Shanghai, China): Cell lysis buffer for Western and IP, Protein Extraction Kit, BCA protein assay kit, enhanced chemiluminescence (ECL) kit, Citrate-EDTA Antigen Retrieval Solution, and Hematoxylin and Eosin Staining Kit. The following reagents were purchased from Gibco (Carlsbad, CA, United States): fetal bovine serum (FBS), minimum essential medium (MEM), Penicillin-Streptomycin antibiotics, non-essential amino acids (NEAA), and 0.25% trypsin solution with EDTA. All other chemicals were of reagent grade.

### 2.2 Animal experimental design and treatment

Sixty male Sprague-Dawley rats (6–8 weeks old, weighing 180–200 g) were purchased from Beijing Sibeifu Bioscience, Co., Ltd. (Beijing, China) (License NO. SCXK [Beijing] 2019-0010). All animals were raised under standard specific pathogen-free (SPF) conditions (temperature, 25°C ± 1°C; humidity, 60% ± 5%) with a 12 h light/dark cycle per day and allowed *ad libitum* eat and drink. Ethical approvals for the animal experiments were obtained from the animal ethics committee of Beijing University of Chinese Medicine (NO. BUCM-4-2022102901-4029).

**FIGURE 2:**
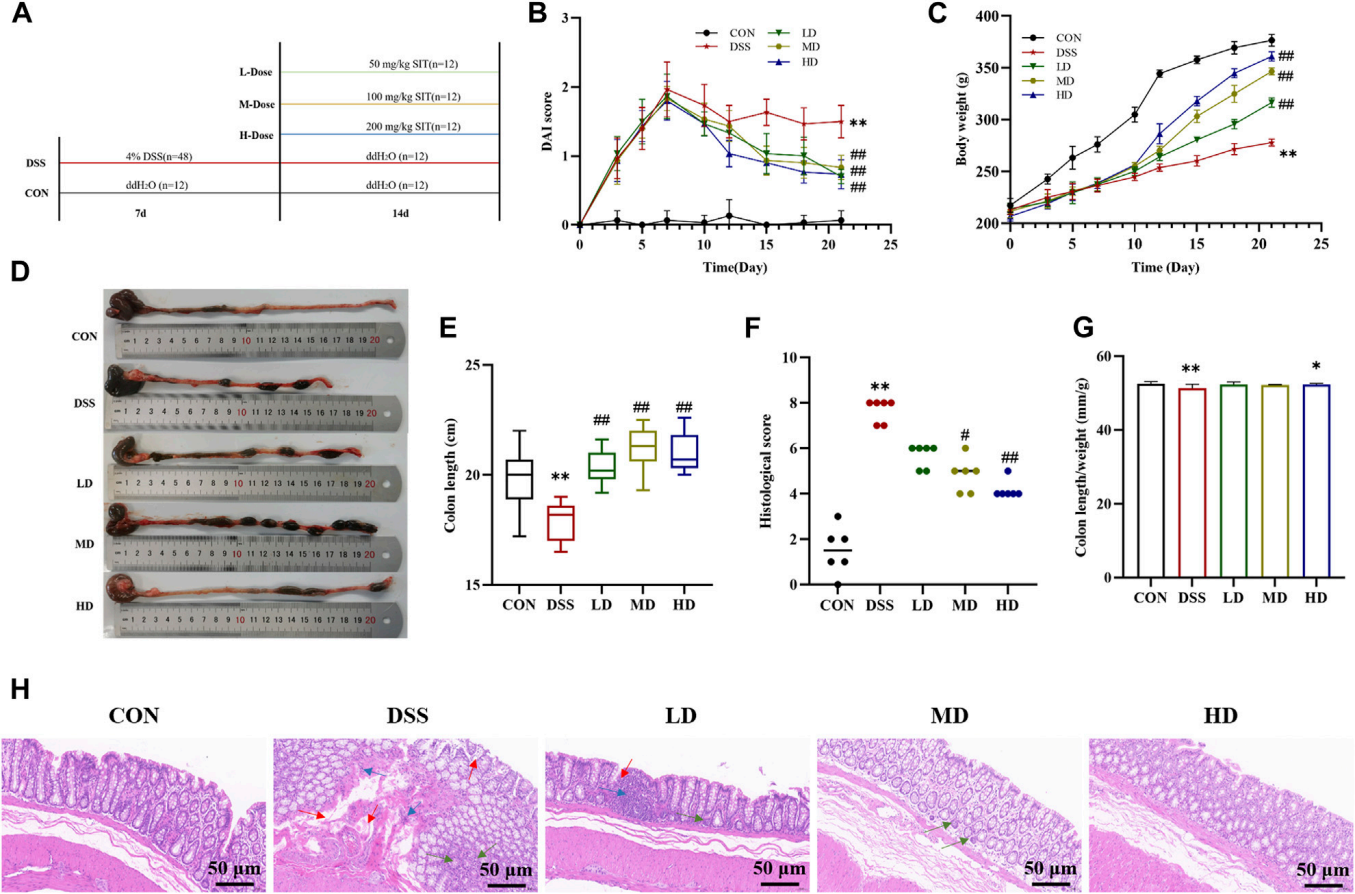
SIT protects rats from DSS-induced UC. **(A)** An experimental design *in vivo*. **(B)** Changes of body weight (N = 10 per group). **(C)** The disease activity index (DAI) scores (N = 10 per group). **(D)** Representative picture of the colon. **(E)** The colon lengths (N = 11 per group). **(F)** The colon length/weight ratio (N = 11 per group). **(G)** The colonic histopathological scores (N = 6 per group). **(H)** Representative histopathologic image of colon. H&E staining, 100 ×; scale bar, 50 μm. Red arrow: distortion or loss of crypts; Blue arrow: infiltration of inflammatory cells; Green arrow: depletion of goblet cells. The data are presented as mean ± SD. CON, control group; DSS, dextran sulfate sodium-induced group; SIT, β-sitosterol; LD, low-dose SIT group; MD, middle-dose SIT group; HD, high-dose SIT group; H&E, hematoxylin and eosin; SD, standard deviation. ^**^ *p* < 0.01 compared with CON group; ^#^ *p* < 0.05, ^##^ *p* < 0.01 compared with DSS group.

This experimental design consists of two steps: a DSS-induced colitis stage and a drug treatment stage as described in Figure 2A. 60 rats were randomly assigned to five groups: 1) Control group (CON): received only double-drilled water; 2) DSS group (DSS): received 4% DSS (molecular weight: 40 000 Da; purity >98%) dissolved in double-drilled water in their daily drinking water; 3) Low-dose group (LD): received DSS and then gavaged with 50 mg/kg of SIT (purity >95%) suspended in 0.1% carboxymethyl cellulose; 4) Middle-dose group (MD): received DSS and then gavaged with 100 mg/kg SIT; 5) High-dose group (HD): received DSS and then gavaged with 200 mg/kg SIT. The intervention period of DSS was from day 1 to day 7 and the period of drug treatment was from day 8 to day 21.

Following the 14-day intervention, rats were all deeply anesthetized after 24 h fasting with 1% sodium pentobarbital (40 mg/kg). After recording the colon length and weight, blood samples were collected and centrifuged for serum. Then the colon specimens were immediately removed and then fixed in 4% paraformaldehyde for histopathological studies or stored at −80°C for molecular biology detection.

### 2.3 Observation of UC symptoms and signs in rats

During this experimental period, body weight, stool status, and rectal bleeding were recorded every day to calculate disease activity index (DAI) scores on a previously established scoring system (Kihara et al., 2003) and listed in Table 1. DAI score is the average of the three categories. All evaluations were performed while unaware of the conditions.

**TABLE 1:** Disease activity index (DAI) scoring system.

Score	Weight loss (%)	Stool consistency	Gross bleeding/rectal bleeding
0	0	Normal	None
1	1–5	Loose stool	Haemoccult positive
2	6–10	Loose stool	Haemoccult positive
3	11–20	Loose stool	Haemoccult positive
4	>20	Diarrhea	Severe bleeding

### 2.4 Histological examination

Hematoxylin and eosin (H&E) detection was conducted with a standard protocol. The colon tissues were fixed in 4% paraformaldehyde and embedded in paraffin. Then, the paraffin-blocked samples were cut into 5-µm sections on slides for staining with hematoxylin and eosin. Slides were scanned using a Panoramic MIDI Scan Whole Slide Scanner (3DHISTECH Ltd., Budapest, Hungary) and viewed with Panoramic Viewer 1.15.4 (3DHISTECH); in addition, histopathological scores for histopathological changes were calculated based on a previously developed scoring system (Dieleman et al., 1998) and listed in Table 2. The individual scoring was blinded to the identity of the slides.

**TABLE 2:** Histopathological scoring system.

Score	Inflammation	Mucosal damage	Regeneration	Crypt damage	Range of lesions (%)
0	None	None	Complete regeneration or normal tissue	None	0
1	Mild	Mucous layer	Alomost complete regeneration	Basal 1/3 damage	1%–25%
2	Moderate	Mucousa and submucosa	Regeneration with crypt depletion	Basal 2/3 damage	26%–50%
3	Severe	Transmural	Surface epithelium not intact	Crypt lost; surface epithelium present	51%–75%
4	-	-	No tissue repair	Crypt and surface epithelium lost	76%–100%

### 2.5 Cell preparation and viability assay

Human epithelial colorectal adenocarcinoma (Caco-2) cell lines (originally obtained from ATCC) were purchased from IMMOCELL (Xiamen, China). The Caco-2 cells were maintained in minimum essential medium (MEM) containing 10% fetal bovine serum (FBS), 100 U/ml of penicillin, 100 μg/mL of streptomycin, and 1% non-essential amino acids supplements. Cells were maintained at 37°C with 5% CO_2_ atmosphere.

Caco-2 cell viability was measured by CCK-8 assay followed by a standard protocol. After preparing single-cell suspensions, cells (5 × 10^4^ cells/well) were added to 96-well plates and continued to culture till 80% confluency. Then, 10 μL/well of CCK-8 solution was added and incubated protected from light for 2 h at 37°C. The 450 nm absorbance values were detected with a multifunctional microplate reader (Thermo, Manassas, United States) to measure the cell growth inhibition rate.

### 2.6 Caco-2 inflammation model establishment and treatment

Cells were cultured overnight with or without SIT (HPLC ≥98%) dissolved in absolute ethanol (concentrations of 8 μg/mL as Low-dose group and 16 μg/mL as High-dose group). Supernatants of cells were discarded and washed with PBS, and 50 mg/mL DSS was added to establish the inflammation model.

### 2.7 Cytokine assays

The serum of rats and supernatant of cells were collected and then calculated for TNF-α, IL-1β, and IL-18 levels using ELISA kits according to the manufacturer’s scheme.

### 2.8 Quantitative polymerase chain reaction

Relative levels of NLRP3, Caspase-1, GSDMD, IL-1β, and IL-18 mRNAs in colonic tissues were analyzed by a quantitative real-time (qRT)-PCR. Total RNA of colonic tissues was isolated using the Total RNA Extraction Kit and cDNA was synthesized by using the mRNA cDNA Synthesis Kit in accordance with the manufacturer’s protocols. The specific primers for NLRP3, Caspase-1, GSDMD, IL-1β, and IL-18 were designed and synthesized by GenePool Biotechnology (Beijing, China) and listed in Table 3. The PCR reaction parameters were predetermined: 95°C for 5 min, followed by a 35 cycle-denaturation for 30 s at 95°C, 55°C for 30 s, and extension for 1 min at 72°C. Each sample underwent three biological replications for statistical analysis to determine significant differences. GAPDH was an internal control and relative gene expression levels were calculated using the 2^−ΔΔCT^ method.

**TABLE 3:** Primers used in quantitative RT-PCR assay.

Gene		Primer sequences (5′-3′)
IL-1β	Forward	CCC​AAC​TGG​TAC​ATC​AGC​ACC​TCT​C
	Reverse	CTA​TGT​CCC​GAC​CAT​TGC​TG
IL-18	Forward	CCG​AAC​AGC​CAA​CGA​ATC​C
	Reverse	ACA​TCC​TTC​CAT​CCT​TCA​CAG​A
Caspase-1	Forward	CTG​GTC​TTG​TGA​CTT​GGA​GGA
	Reverse	TCA​GTG​GTT​GGC​ATC​TGT​AGT
NLRP3	Forward	AGA​CCT​CCA​AGA​CCA​CGA​CTG
	Reverse	CAT​CCG​CAG​CCA​ATG​AAC​AGA
GSDMD	Forward	GCA​GTG​GTG​AGC​AGG​TAG​AG
	Reverse	CCA​GAG​CCT​TAG​TAG​CCA​GTA​G
GAPDH	Forward	TGG​AGT​CTA​CTG​GCG​TCT​T
	Reverse	TGT​CAT​ATT​TCT​CGT​GGT​TCA

### 2.9 Western blot analysis

The total proteins of tissues and cells were extracted using Western and IP lysate buffer, BCA protein assay kit was applied to determine protein concentration. After homogenization, an equal quantity of proteins was separated with SDS-PAGE and transferred to PVDF membranes (Millipore corp., Massachusetts, United States), then blocked with 5% BSA. Primary antibodies of NLRP3 (dilution 1:1 000), Caspase-1 (dilution 1:2 000), cleaved-Caspase-1 (dilution 1:1 000), GSDMD (dilution 1:2 000), GSDMD-N (dilution 1:1 000), p65 (dilution 1:1 000), p-p65 (dilution 1:1 000), IκBα (dilution 1:1 000), p-IκBα (dilution 1:1 000), ZO-1 (dilution 1:5 000), occludin (dilution 1:1 000), and β-Actin (dilution 1:5 000) were incubated overnight at 4°C. After being washed with TBST three times, membranes were then incubated with secondary antibodies against Goat Anti-Rabbit IgG H&L (HRP) (dilution 1:5 000) or Goat Anti-Mouse IgG H&L (HRP) (dilution 1:5 000) for 1 h at 37°C, and bands were visualized with an ECL kit captured with Tanon 5200 Chemiluminescent Imaging System (Tanon, Shanghai, China). Finally, the relative grey values of the target proteins blots normalized to β-Actin were analyzed by the ImageJ software (National Institutes of Health, United States).

### 2.10 Statistical analysis

Statistical analysis was performed by SPSS 26.0 system. Quantitative results were expressed as arithmetic mean plus or minus the standard deviation (SD) and analyzed using one-way analysis of variance (ANOVA) followed by least significant difference (LSD)’s multiple-comparison test, while Kruskal-Wallis test was performed for difference analysis of non-parametric data. *p*-value < 0.05 was considered statistically significant.

## 3 Results

### 3.1 SIT alleviates DSS-induced UC

In order to investigate the therapeutic effects of SIT *in vivo*, UC model rats were induced by 4% DSS for 7 consecutive days. During the experiment period, rats in the control group remained in a normal state, and body weight was rising steadily, while those in the model group represented a poor mental state with disheveled fur, decreased feeding, persistent fecal bleeding, and obvious body weight loss. Compared to the control group DAI score in the DSS-induced model group was significantly increased, which was consistent with the clinical characteristics. Notably, administration of SIT recovered the body weight and colon length gradually (Figures 2C–E) as well as decreased DAI score (Figure 2B) in DSS-induced rats, especially at the dose of 200 mg/kg.

Furthermore, colonic shortening caused by DSS was evidently mitigated after SIT intake. As can be seen from Figure 2F, DSS in the UC group led to a significant decrease in colon length/weight ratio compared with the control group. High dose of SIT agonist partly offset the decrease of colon length/weight ratio. Under microscopy, results by H&E staining showed that there were pathological lesions and superficial inflammation in the DSS-induced colonic tissues characterized by inflammatory cell infiltration, crypt architectural distortion or absence and mucosa defects or damage. Consistent with the remission of clinical signs, the pathological changes were improved to varying degrees following the administration of SIT (Figures 2F,G). Collectively, these results indicated that SIT is protective against DSS-induced colitis and the effects might be dose-dependent.

### 3.2 Effects of SIT on the viability of Caco-2 cells

**FIGURE 3:**
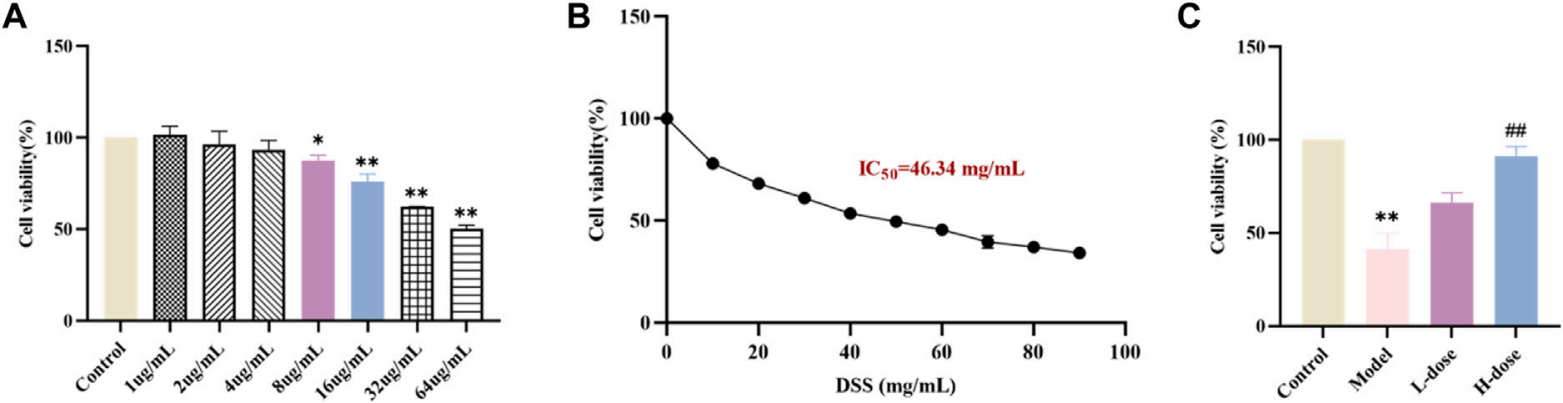
SIT protects Caco-2 cells from DSS-induced damage. **(A)** The viability of Caco-2 cells under the treatment with different concentrations SIT-containing medium for 24 h, and the viability was assayed by CCK-8. **(B)** Caco-2 cells were cultured with 10, 20, 30, 40, 50, 60, 70, 80, and 90 mg/mL DSS-containing medium for 24 h. **(C)** The cells were first cultured with SIT-containing medium for 24 h, incubated in 50 mg/mL DSS-containing medium for 6 h, and then replaced with fresh complete medium to culture for 18 h. N = 6 per group. The data are presented as mean ± SD. DSS, dextran sulfate sodium-induced group; SIT, β-sitosterol; SD, standard deviation. ^*^ *p* < 0.05, ^**^ *p* < 0.01 compared with Control group; ^##^ *p* < 0.01 compared with Model group.

Before the formal *in vitro* experiments, toxicity evaluation of different concentrations of SIT (1, 2, 4, 8, 16, 32, and 64 μg/mL) on Caco-2 was carried out using the CCK-8 assay. After the 24 h-intervention, we observed that there was a significant decline in cell viability of Caco-2 following the higher concentrations of SIT (Figure 3A). Based on this, we chose the relatively safe concentrations for low-dose SIT (LD, 8 μg/mL) and high-dose SIT (HD, 16 μg/mL), respectively. In addition, at the concentration of 46.34 mg/mL DSS, growth of 50% cells was inhibited (Figure 3B). For convenience, we used 50 mg/mL DSS for subsequent experimental Caco-2 modeling, and found that SIT serves protectively against DSS-induced Caco-2 damage, (Figure 3C).

### 3.3 SIT inhibits proinflammatory mediators

**FIGURE 4:**
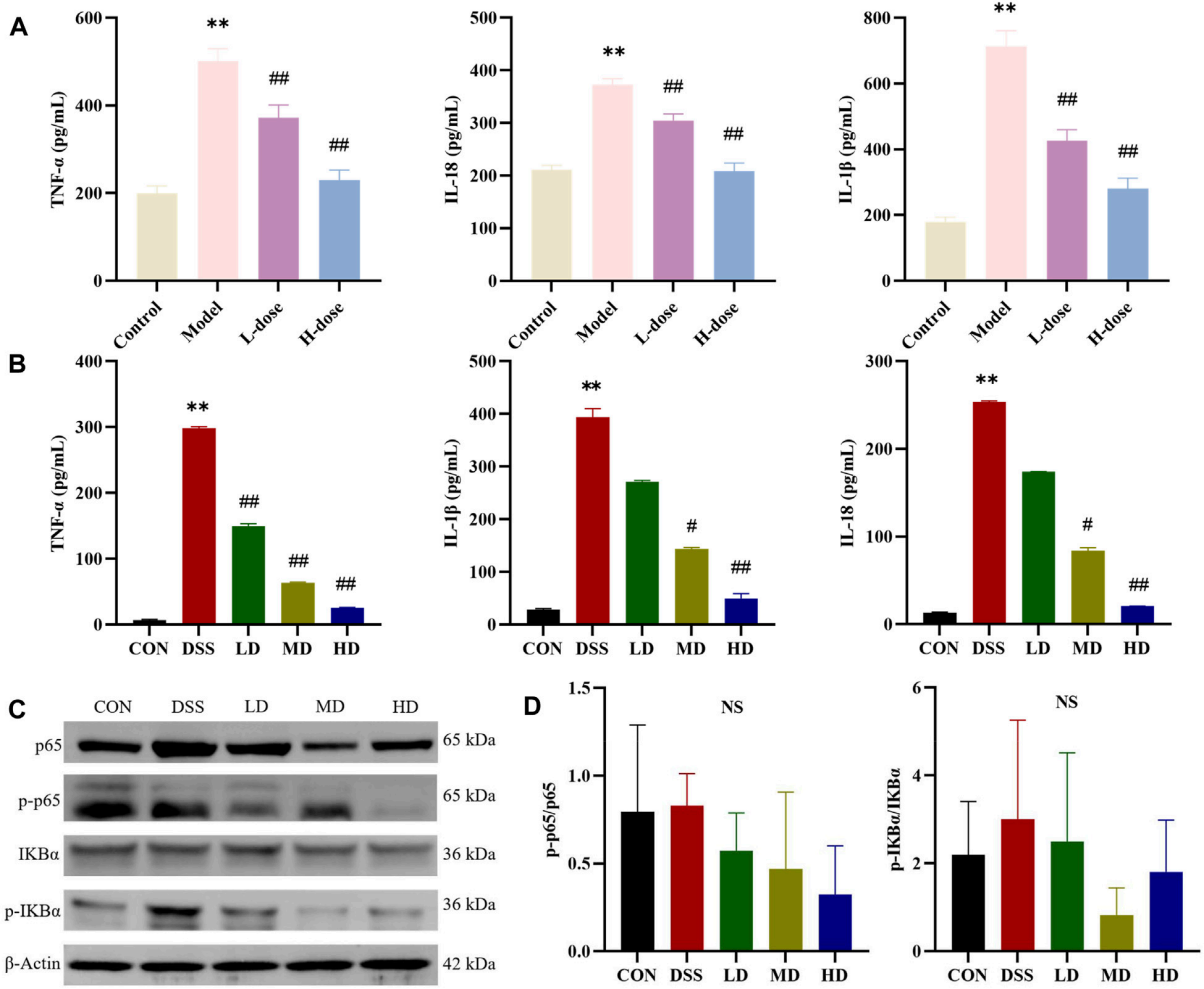
SIT regulates the pro-inflammatory cytokines and NF-κB pathway. The expression levels of TNF-α, IL-1β, and IL-18 in serum **(A)** and Caco-2 cells **(B)** were analyzed by ELISA (N = 6 per group). **(C)** Gel electrophoresis images of p-p65, p65, p-IκBα, and IκBα were analyzed by Western blot. **(D)** Analysis of protein expression levels (N = 3 per group). The data are presented as mean ± SD. NF-κB, nuclear transcription factor-kappa B; CON, control group; DSS, dextran sulfate sodium-induced group; SIT, β-sitosterol; LD, low-dose SIT group; MD, middle-dose SIT group; HD, high-dose SIT group; TNF, tumor necrosis factor; IL, interleukin; ELISA, enzyme-linked immunosorbent assay; SD, standard deviation. ^**^ *p* < 0.01 compared with CON or Control group; ^#^ *p* < 0.05, ^##^ *p* < 0.01 compared with DSS or Model group; NS, not statistically significant.

To validate the suppressive effects of SIT on inflammation involved in UC, we detected the levels of inflammatory-related factors. The ELISA results showed that there was a significantly increase of TNF-α, IL-1β, and IL-18 levels after DSS treatment compared with the control group in rats and SIT effectively decreased the expression (Figure 4A). We further noticed the protective effects of SIT on Caco-2 from inflammation after being exposed to DSS (Figure 4B).

### 3.4 SIT regulates the NF-κB inflammatory pathway

NF-κB signaling participates in the regulation of inflammatory response. We wondered whether the anti-inflammatory role of SIT was associated with the signaling. The expression levels of the major proteins p-p65, p65, p-IκBα, and IκBα in the NF-kB signaling pathways were consequently measured by Western blot. The experimental results showed that the levels of p-p65 and p-IκBα in DSS-induced colonic tissues were abnormally higher compared to the control group. In comparison with the DSS group, the protein expression ratio of p-p65/p65 and p-IκBα/IκBα were attenuated after stimulation with SIT (Figures 4C,D) while none were statistically significant. These results indicate that NF-κB signaling might be involved in the reduction of DSS-induced UC inflammation by SIT administration.

### 3.5 SIT suppresses critical indicators activity of pyroptosis

**FIGURE 5:**
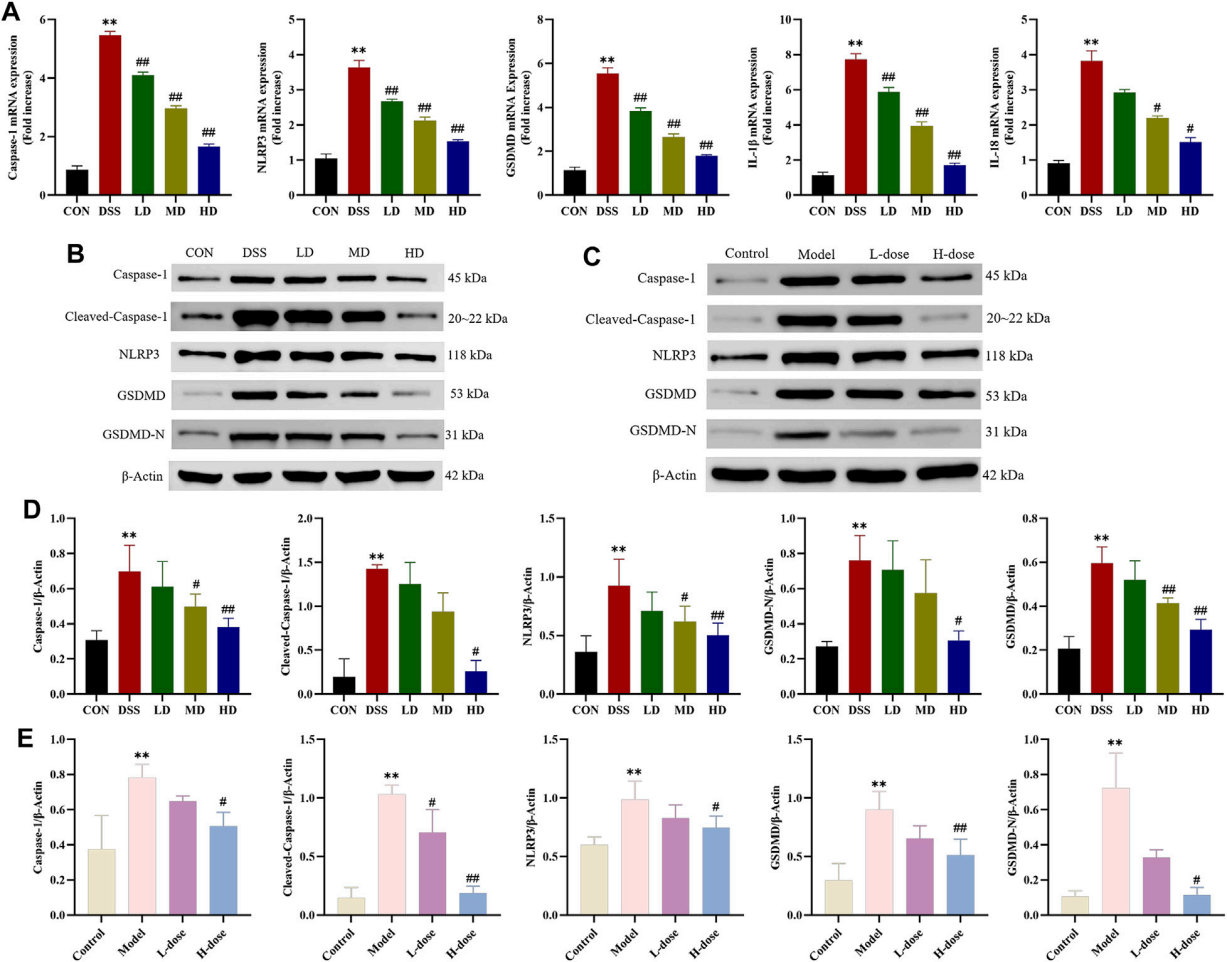
SIT inhibits DSS-induced Caspase-1 mediated pyroptosis pathway activation. **(A)** The mRNA levels of Caspase-1, NLRP3, GSDMD, IL-1β, and IL-18 in colon tissues. Gel electrophoresis images of Caspase-1, Cleaved-Caspase-1, NLRP3, GSDMD, and GSDMD-N in colon tissues **(B)** and Caco-2 cells **(D)** were analyzed by Western blot. Analysis of protein expression levels was analyzed and shown in **(C)** and **(E)**. N = 3 per group. The data are presented as mean ± SD. CON, control group; DSS, dextran sulfate sodium-induced group; SIT, β-sitosterol; LD, low-dose SIT group; MD, middle-dose SIT group; HD, high-dose SIT group; NLRP3, nucleotide-binding oligomerization domain (Nod)-like receptor thermal protein domain associated protein 3; GSDMD, gasdermin D; GSDMD-N, gasdermin D-N terminal; IL, interleukin; SD, standard deviation. ^**^ *p* < 0.01 compared with CON or Control group; ^#^ *p* < 0.05, ^##^ *p* < 0.01 compared with DSS or Model group.

Pyroptosis is a type of programmed cell death (PCD) and participates in inflammatory disease processes involved in UC. For this reason, we further measured the expression of critical indicators of NLRP3/Caspase-1/GSDMD-mediated pyroptosis to explicit the anti-inflammatory effects of SIT. RT-qPCR analysis was carried out to assess the gene expression of Caspase-1, NLRP3, GSDMD, IL-1β, and IL-18 in colonic tissues. When compared to the control group, the mRNA expression levels of the above genes were upregulated in the DSS group while significantly downregulated with SIT administration (Figure 5A).

As expected, Western blot analysis showed that protein expression levels of Caspase-1, Cleaved-Caspase-1, NLRP3, GSDMD, and GSDMD-N distinctly increased after DSS treatment in colonic tissues and Caco-2 cells and significantly counteracted following the intervention with SIT (Figures 5B–E). Meanwhile, ELISA indicated that SIT treatment significantly inhibited DSS-induced increased levels of IL-1β and IL-18 in serum and cell supernatant; it has been shown that secretion of cytokines IL-1β and IL-18 were associated with the NLRP3 inflammasome and induced an inflammatory cell death mode termed as pyroptosis (He et al., 2015). Comprehensively, the above results suggest that SIT plays a possible therapeutic role in UC via regulating Caspase-1-mediated pyroptosis.

### 3.6 SIT attenuates colonic mucosal barrier

**FIGURE 6:**
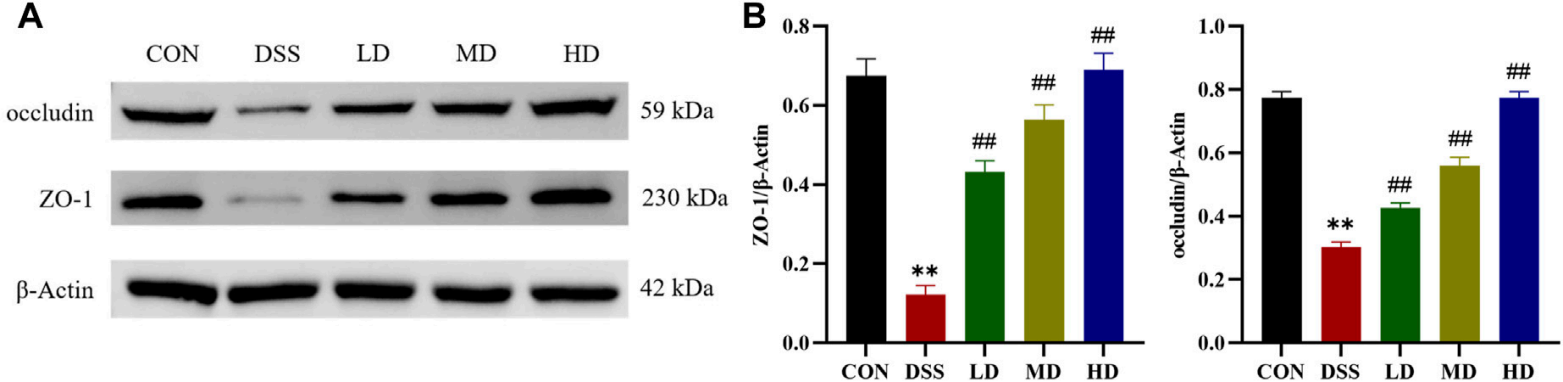
SIT alleviates colitis from barrier damage. **(A)** Gel electrophoresis images of occludin and ZO-1 in colon tissues were analyzed by Western blot. **(B)** Analysis of protein expression levels (N = 3 per group). The data are presented as mean ± SD. CON, control group; DSS, dextran sulfate sodium-induced group; SIT, β-sitosterol; LD, low-dose SIT group; MD, middle-dose SIT group; HD, high-dose SIT group; ZO, zonula occludens; SD, standard deviation. ^**^ *p* < 0.01 compared with CON group; ^##^ *p* < 0.01 compared with DSS group.

Tight junction proteins are the essential parts for the maintenance of the gut mucosal barrier integrity, which could against the invasion of luminal detrimental substances and lower the risk of microbe-induced inflammation (Parikh et al., 2019). Therefore, we further verified whether the protective mechanism of SIT on the colon is related to the restoration of intestinal barrier function. ZO-1 and occludin, are important two tight junction-associated proteins (Ji et al., 2016), which were measured by Western blot. The expression levels of ZO-1 and occludin were decreased compared with the control group, which were significantly reversed by SIT as provided in Figure 6, suggesting that SIT could alleviate colitis through enhancing mucosal barrier integrity.

## 4 Discussion

UC is a main subtype of chronic relapsing inflammatory bowel disease and is highly prevalent worldwide. The nature of UC is complex and the pathogenesis of UC has not been well elucidated yet. Current therapeutic agents are not entirely desirable in terms of potency with many side effects, erratic efficacy, and recurrence or failure after reduction or termination of administration as well as a heavy financial burden (Bressler et al., 2015; Berends et al., 2019). Hence new alternative therapeutic medication from natural products is of high interest in research. SIT is a well-known bioactive phytosterol naturally plentiful in dietary and non-dietary plant cell membranes, accounting for about 65% of human herbal nutrition forming (Weihrauch and Gardner, 1978). They are not only highly found in lipid-rich plant foods such as nuts, seeds, and legumes but also in vegetables and fruits (Khan et al., 2022). Over the past few decades, research on SIT was at an ever-accelerating pace and has suggested it may exert preventable and therapeutic effects on UC (Lee et al., 2012; Ding et al., 2019) with much less research on its role in pyroptosis.

DSS-induced experimental colitis is a reliable and mature animal model, which resembles clinically pathological symptoms and histological features in chronic UC (Tessner et al., 1998). In our current study, we demonstrated that SIT did significantly relieve the body weight loss in rats with 4% DSS-induced UC, with an increased DAI score, a main indicator in the assessment of the severity of UC. In addition, colonic shortening, another indirect indicator in the evaluation of the severity of UC, was attenuated by SIT treatment, indicating that SIT has therapeutic potential in UC rats. For colon pathological injury, we detected histological analysis and found that administration of SIT could improve cryptal glands and submucosa and reduce inflammatory cells infiltrating. Also, with the increase of SIT dosages, the therapeutic effects improve correspondingly, suggesting that SIT may be a potential drug of choice for UC treatment.

Self-limiting acute inflammation is crucial for the body to eliminate the danger and restore homeostasis, while unresolved inflammation contributes to the pathogenesis of autoimmune diseases including UC (Afonina et al., 2017)^.^ An increasing body of evidence has shown that aberrant inflammatory responses are the key contributors to the progression and exacerbation of UC. Pyroptosis, a novel type of programmed cell death, is mainly elicited by either classical Caspase-1-mediated or non-classical Caspase-11/4/5/11-mediated pathways in inflammation (Fang et al., 2020). Research has indicated that both Caspase-1-mediated and Caspase-11-mediated pyroptosis are closely linked to the development of UC (Chao et al., 2020), in which NLRP3 inflammasome plays a key role (Chen et al., 2019; Zhen and Zhang, 2019). NLRP3 first interacts with ASC and combines with Caspase-1 to assemble the inflammasome complex (Lu et al., 2014). Cleaved Caspase-1 then cleaves GSDMD to the N-terminal fragment (GSDMD-N). After that, the activated N-terminal domain of GSDMD translocates and forms cell membrane pores, thus resulting in the occurrence of pyroptosis and triggering the damage of the epithelial cells in the gut, and releasing pro-inflammatory cytokines (Yuan et al., 2018). The secretion of IL-1β and IL-18 can further amplify and perpetuate the inflammatory reaction (He et al., 2015; Shi et al., 2015). Of note, as the co-substrate of multiple inflammasomes and two main pathways of pyroptosis, GSDMD performs the primary function of pyroptosis executioner (Kayagaki et al., 2015; Shi et al., 2015).

Inflammatory cytokines IL-1β and IL-18 are the main markers for pyroptosis and their overproduction was found in various regions of the colon in UC active patients (Thinwa et al., 2014). Recent findings suggested that both Caspase-1 and Caspase-11 activation possess the function to induce the release of IL-1β and IL-18, but only Caspase-1 could directly cleave them (Man and Kanneganti, 2015); in addition, epithelium IL-18 secretion was independent on NLRP3 but dependent on Caspase-1 (Song-Zhao et al., 2014). Numerous experimental studies on animals have demonstrated inhibiting Caspase-1-dependent pyroptosis could protect against DSS-induced colitis (Tian et al., 2020; Cui et al., 2022; Xue et al., 2022). In view of this, we observed the regulation effect of SIT on inflammatory mediators in our present study. The results showed that SIT alleviated DSS-induced inflammation both in UC rats and Caco-2 cells with downregulation of pro-inflammatory cytokines including TNF-α, IL-1β, and IL-18. Furthermore, we explored the canonical Caspase-1 dependent pathway which underlies UC and we found that SIT treatment downregulated the protein levels of Caspase-1, cleaved-Caspase-1, NLRP3, GSDMD, and GSDMD- N in tissues and cells as expected. Taken together, SIT did ameliorate experimental colitis by inhibiting the NLRP3/Caspase-1/GSDMD-dependent pyroptosis pathway.

Furthermore, NF-κB pathway played an important role in the inflammatory responses of UC (Tong et al., 2021). In mammals, NF-κB family can form homo- or heterodimers of which the most common form is a dimer of p50 and p65. Normally, NF-κB remains inactive with the combination of members of IKB family (Lawrence, 2009). Clinical studies also have demonstrated excessive inflammation activated by NF-κB exists in UC (Zhou et al., 2018). Activation of NF-κB signaling cascade response was reported to participate in the regulation of NLRP3 inflammasome transcription and Caspase-1 has been proven as an active activator of NF-κB (Danelishvili et al., 2011; Hu et al., 2019; Zhen and Zhang, 2019). Previous studies have demonstrated that SIT could reduce the secretion of inflammatory mediators such as TNF-α and IL-1β; in addition, SIT has the ability to suppress the initiation of NLRP3 and the activation of Caspase-1 as well as partial inhibition of NF-κB *in vitro* (Liao et al., 2018). In this study, we also assessed the major proteins of NF-κB pathway. We observed the over-expressed phosphorylation levels of p65 subunit and IκBα, the protein content in colon tissues was suppressed under the SIT treatment. However, it cannot be stated with certainty that SIT-mediated inflammation alleviation is associated with NF-κB pathway in UC in light of our present results.

The mechanical barrier is the most important barrier among the intestinal mucosal barrier, with the structural basis consisting of intact intestinal epithelial cells and tight junction (TJ) proteins between the cells (Odenwald and Turner, 2017). Once the intestinal barrier was disrupted, the bacteria and toxins from the gut would translocate to the mucosa and activate deleterious intestinal inflammation (Liao et al., 2018; Zhang et al., 2018). Previous literature has demonstrated that the dysfunction of intestinal barrier integrity is responsible for the exacerbation of inflammatory bowel disease including UC (Zhao et al., 2020). Therefore, restoring the intestinal barrier integrity can act in preventing or treating UC. To gain further insight into the actions of SIT on the colonic barrier function, we detected the expression levels of the TJ-associated proteins ZO-1 and occludin. Western blot results suggested that the protein expression of ZO-1 and occludin were markedly weakened by DSS treatment while increasing in response to SIT administration. These results suggest that SIT could protect the gut against the occurrence of intestinal inflammation by maintaining mucosal barrier integrity, and therefore halt UC progression.

## 5 Conclusion

**FIGURE 7:**
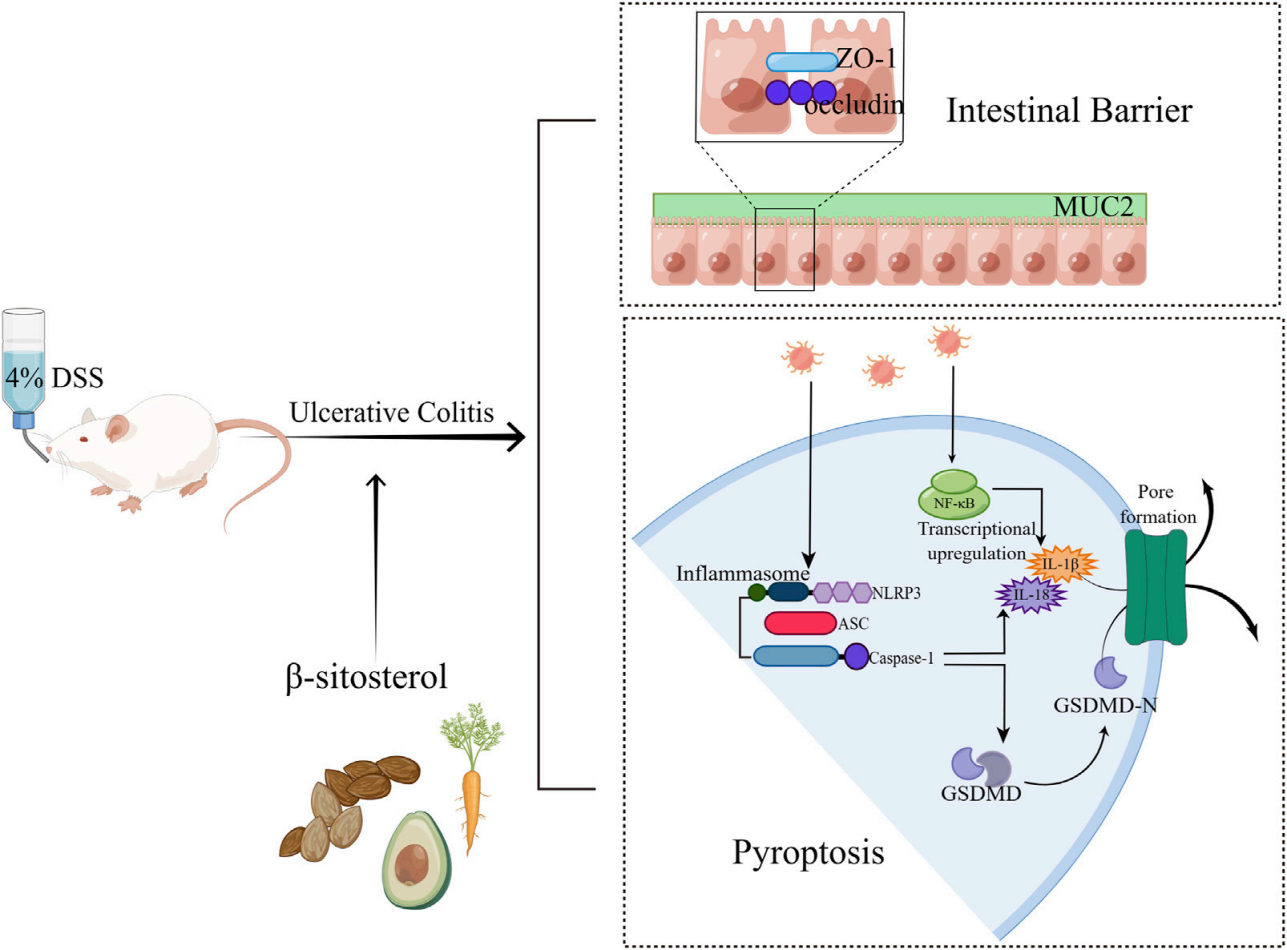
Overview of the effects of SIT on DSS-induced UC (By FigDraw, ID: SSITU8168e).

In summary, SIT not only inhibits the production of pro-inflammatory mediators and suppresses pyroptosis via suppression the NLRP3/Caspase-1/GSDMD-dependent pathway, but also enhances the function of the intestinal mucosal barrier through regulation of epithelial tight junction proteins expression (Figure 7). These findings indicate that SIT is an effective drug candidate and may be potential in clinical applications of UC treatment in the future.

As far as we know, it is the first evidence to show that SIT is effective in regulating pyroptosis in experimental UC. Although many studies have shown that SIT has good safety profiles, its poor stability, low water solubility, and short half-life also confine its broader application, and await to be addressed.

## Data Availability

The original contributions presented in the study are included in the article/Supplementary Material, further inquiries can be directed to the corresponding authors.

## Abbreviations

ASC, apoptosis-associated speck-like protein; CON, control; DAI, disease activity index; DSS, dextran sulfate sodium; ELISA, enzyme-linked immunosorbent assay; GAPDH, glyceraldehyde-3-phosphate dehydrogenase; GSDMD, gasdermin D; GSDMD-N, gasdermin D-N-terminal; HD, high-dose; HRP, horse radish peroxidase; IHC, immunohistochemistry; IL, interleukin; IBD, inflammatory bowel disease; IL, interleukin; LD, low-dose; MD, middle-dose; NF-κB, nuclear transcription factor-kappa B; NLRP3, nucleotide-binding oligomerization domain (Nod)-like receptor thermal protein domain associated protein 3; PVDF, polyvinylidene fluoride; TNF, tumor necrosis factor; SD, standard deviation; SDS-PAGE, sodium dodecyl sulfate-polyacrylamide gel; UC, ulcerative colitis; ZO, zonula occludens.
