# Supplementary figures and images for: β-sitosterol alleviates dextran sulfate sodium-induced experimental colitis via inhibition of NLRP3/Caspase-1/GSDMD-mediated pyroptosis (part 1 of 2)
Source: Front Pharmacol. 2023 Oct 26;14:1218477. doi: 10.3389/fphar.2023.1218477 (PMC10637366; doi:10.3389/fphar.2023.1218477)

occludin

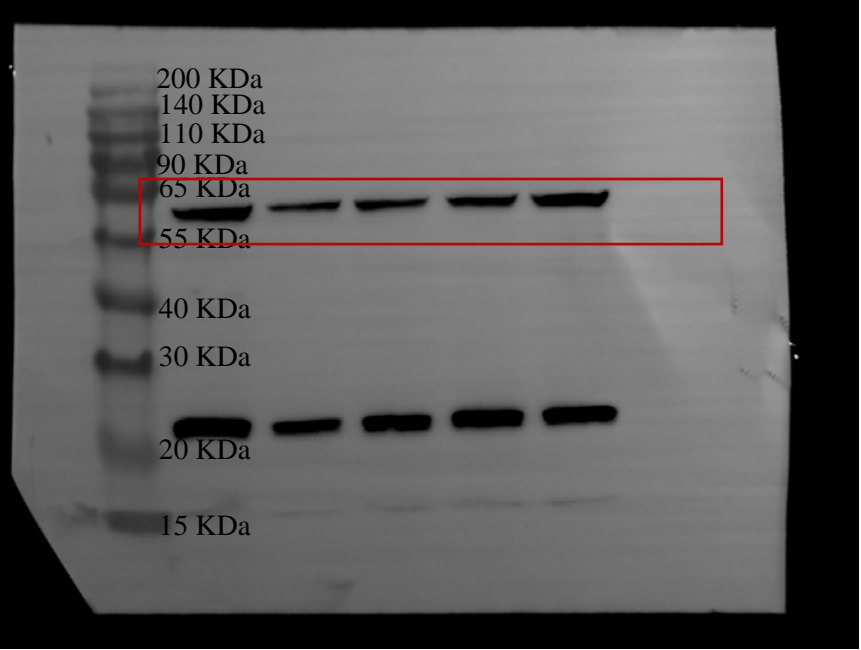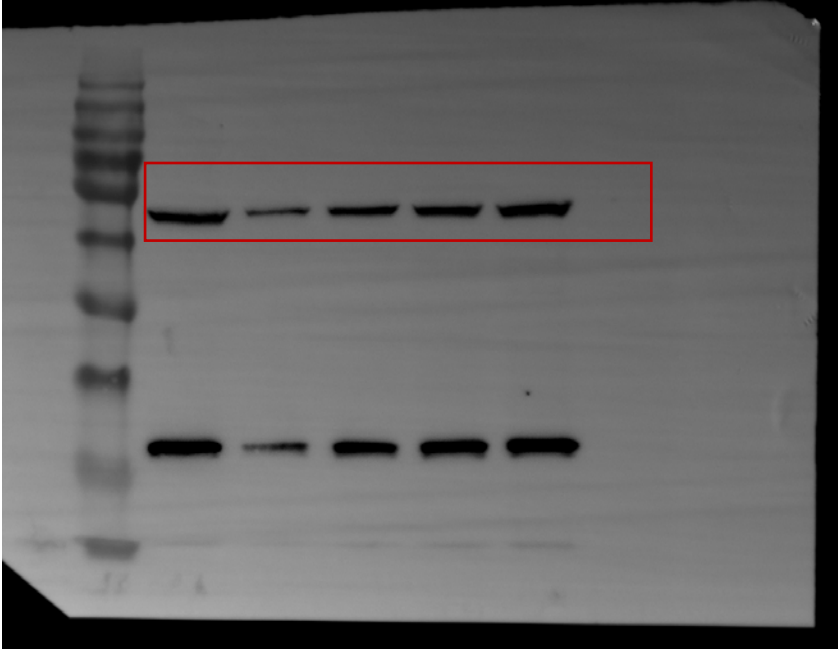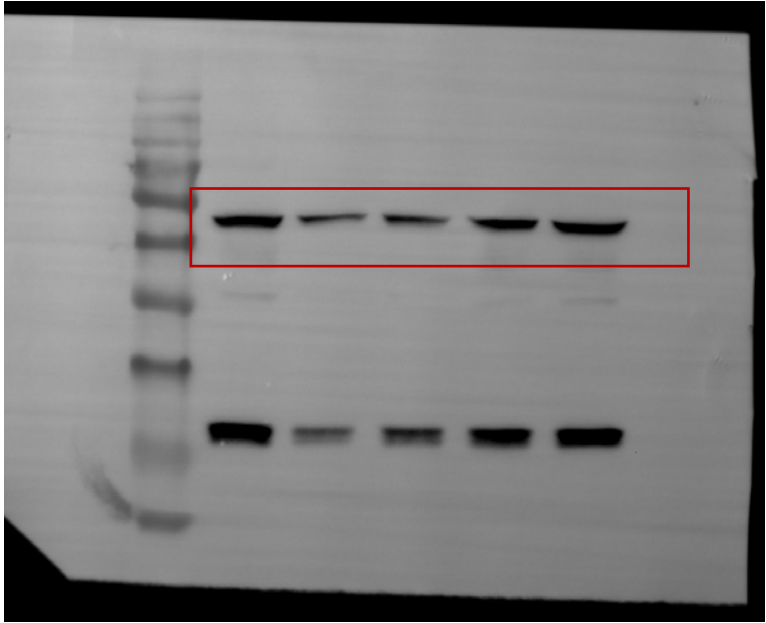

ZO-1

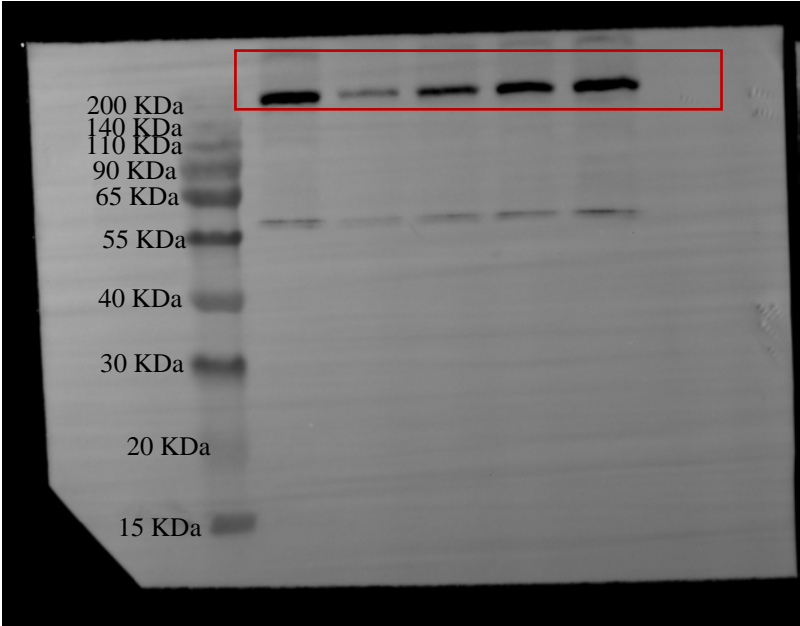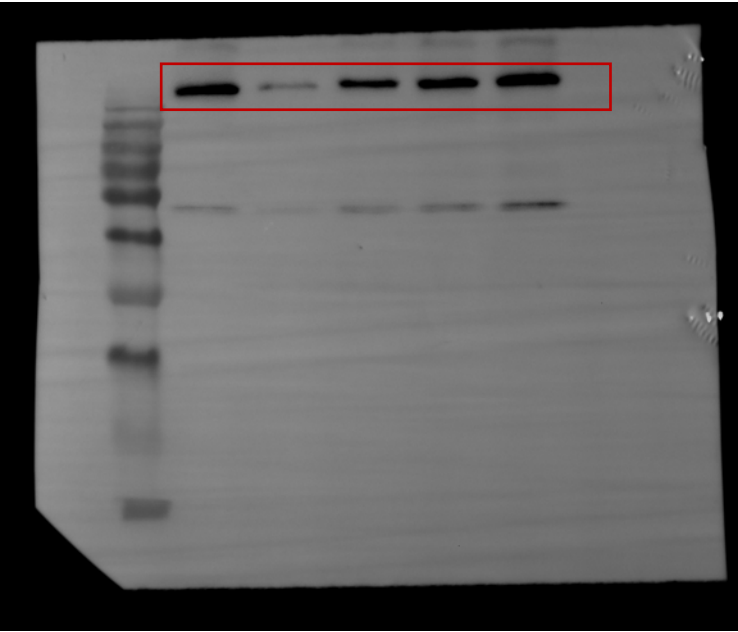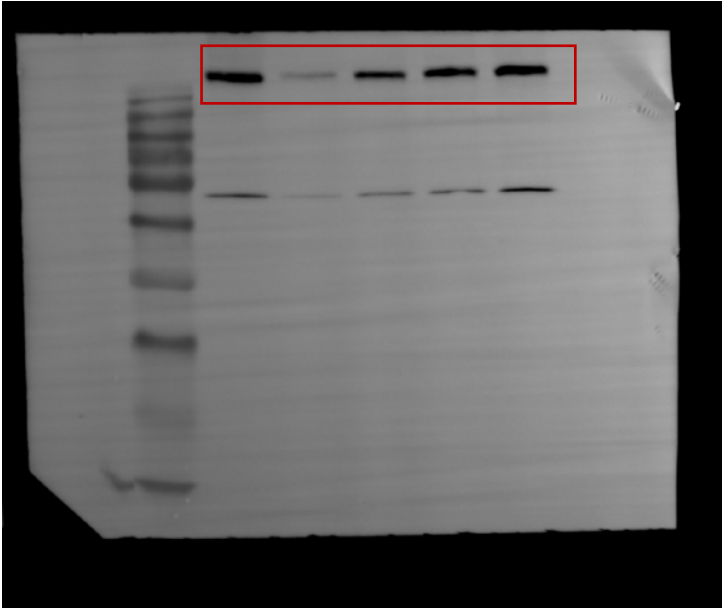

$\beta$ -Actin

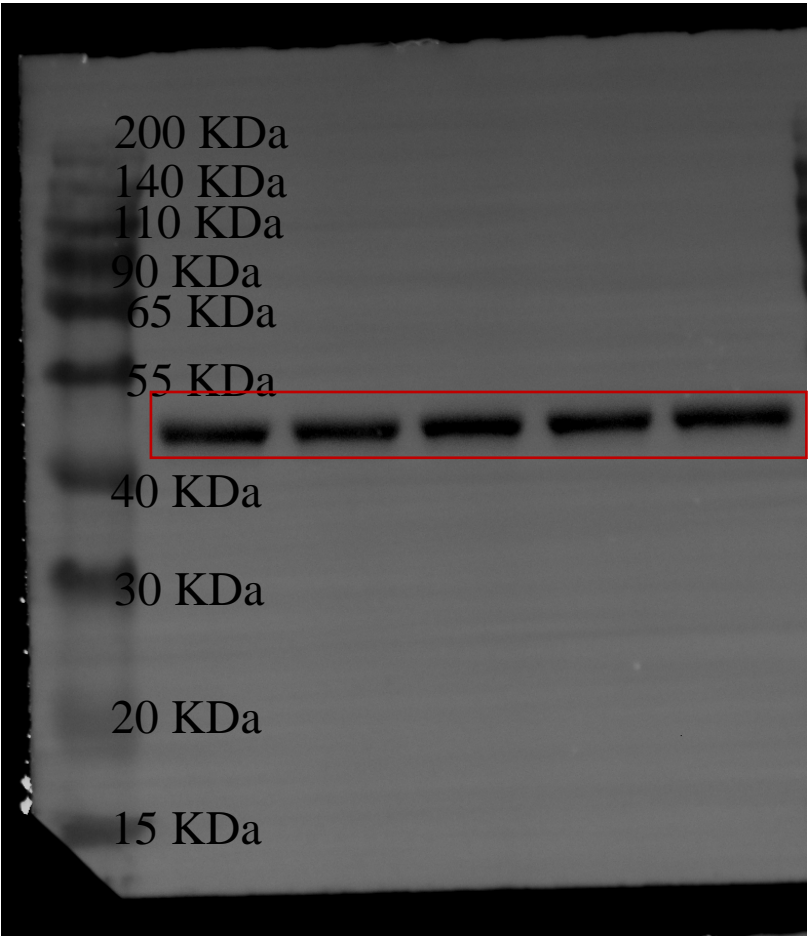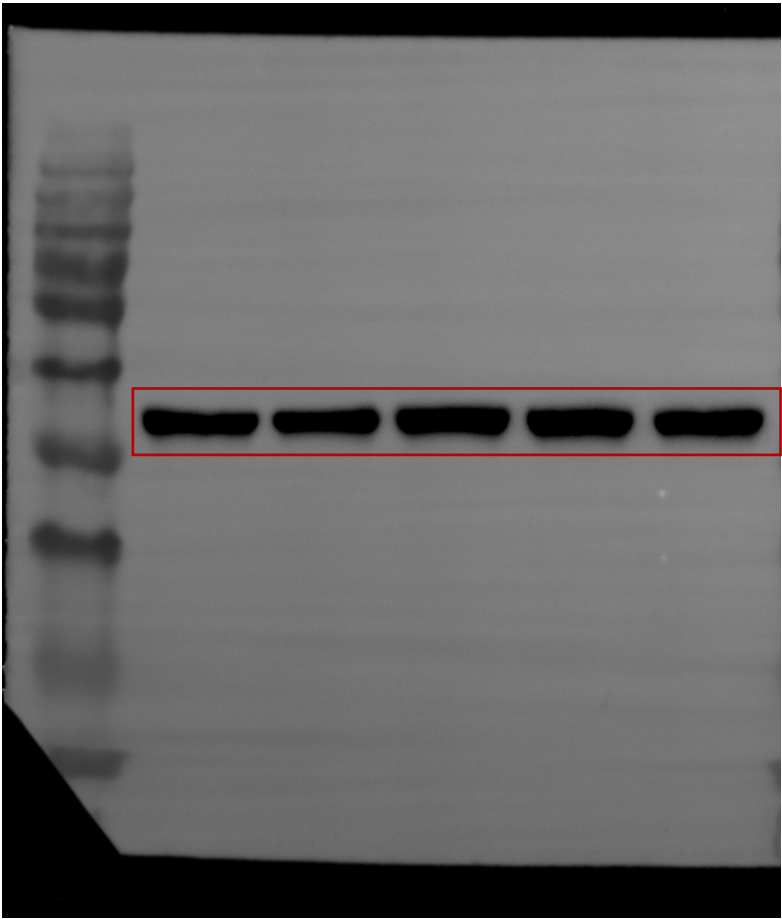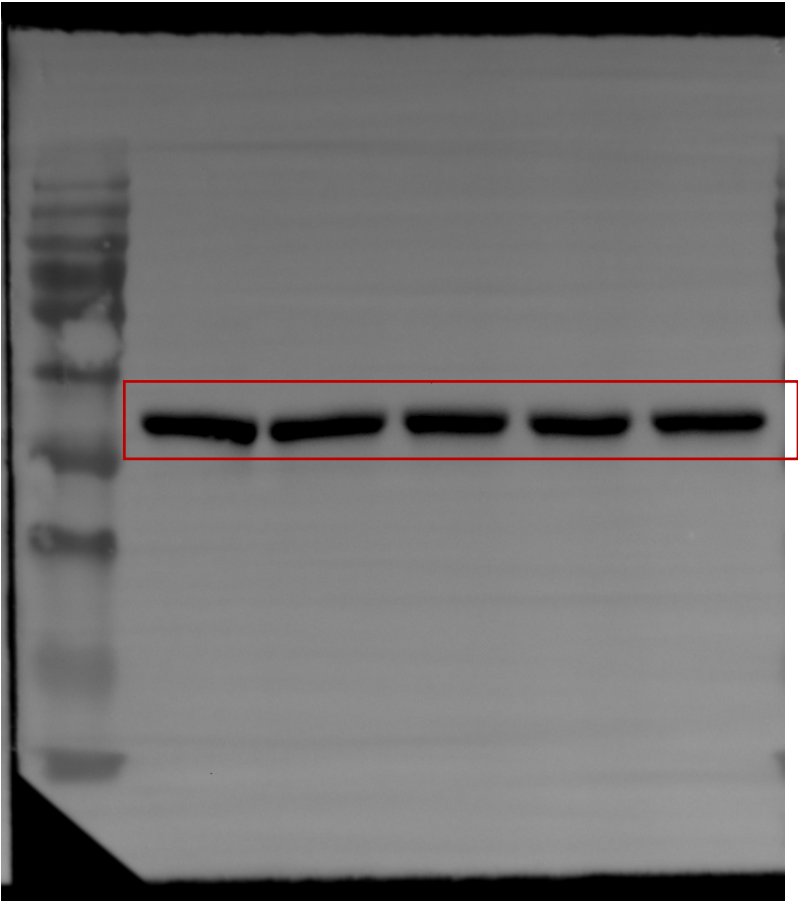

Supplement: Supplementary file 1 [file DataSheet3.ZIP › original data of figure 6/1. WB image of tight junctions.pdf]

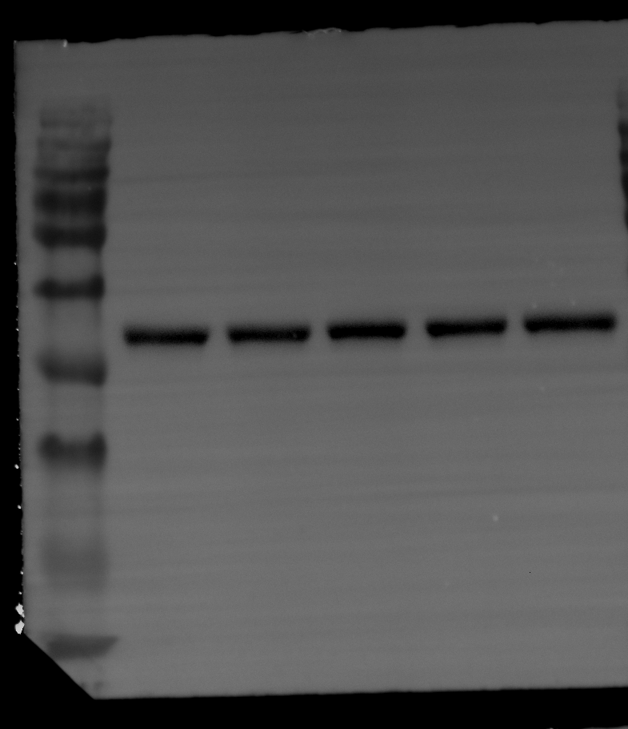

Supplement: Supplementary file 1 [file DataSheet3.ZIP › original data of figure 6/Actin-1.tif]

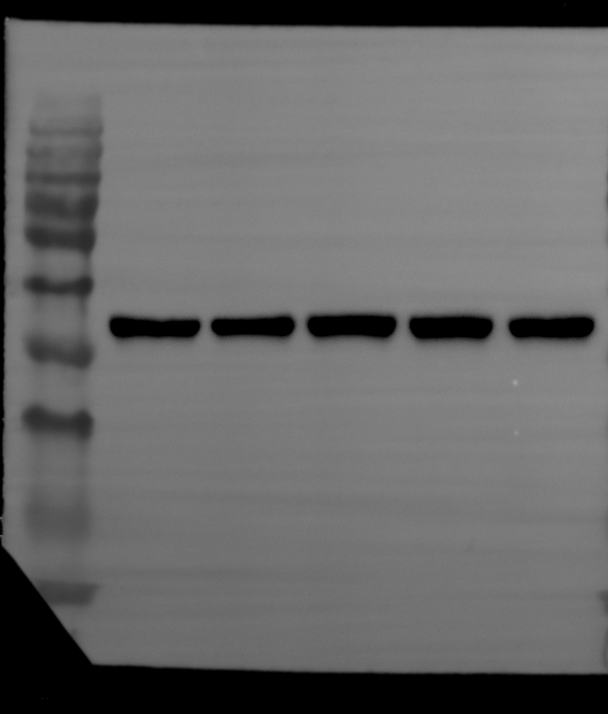

Supplement: Supplementary file 1 [file DataSheet3.ZIP › original data of figure 6/Actin-2.tif]

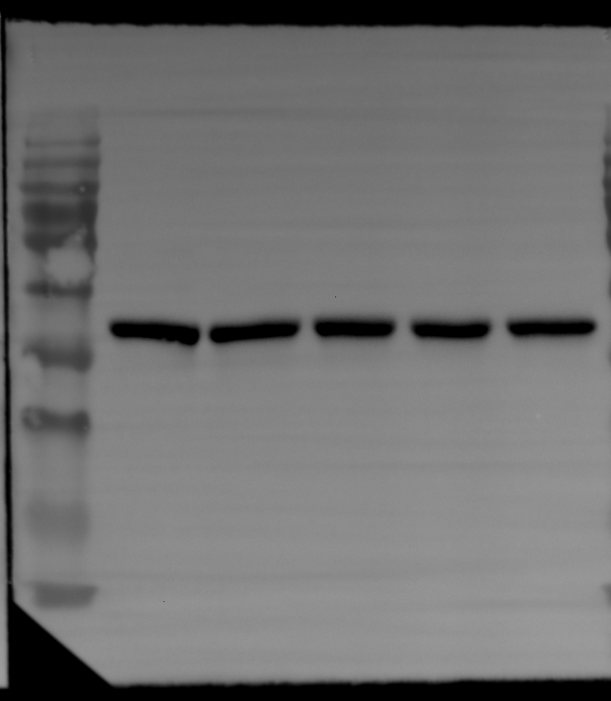

Supplement: Supplementary file 1 [file DataSheet3.ZIP › original data of figure 6/Actin-3.tif]

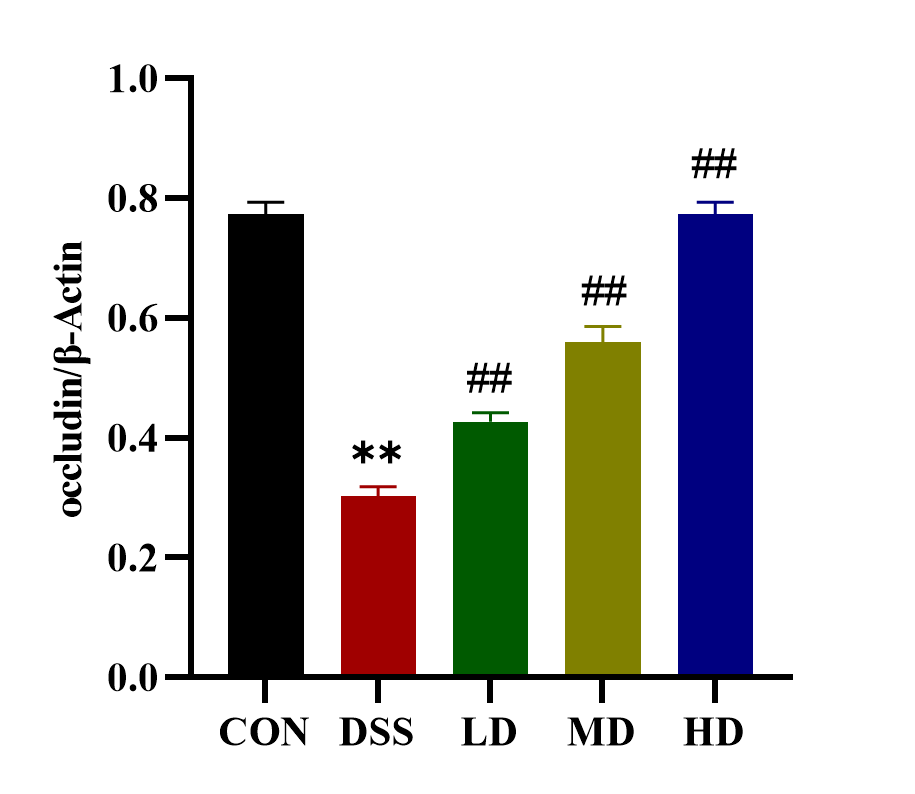

Supplement: Supplementary file 1 [file DataSheet3.ZIP › original data of figure 6/Figure1 occludin.tif]

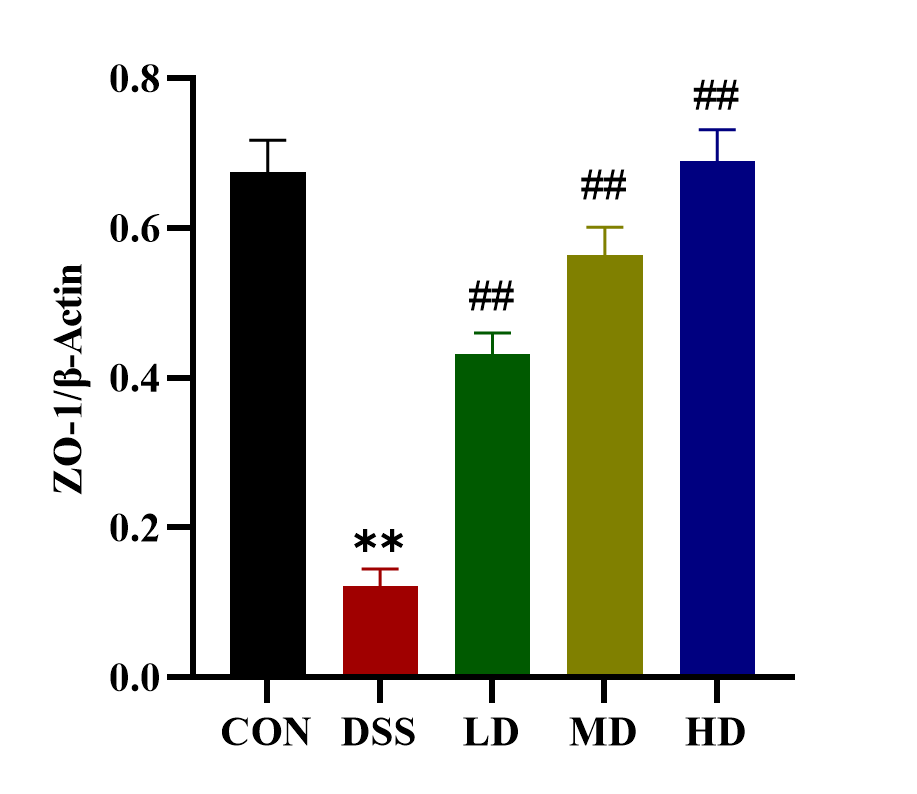

Supplement: Supplementary file 1 [file DataSheet3.ZIP › original data of figure 6/Figure2 ZO-1.tif]

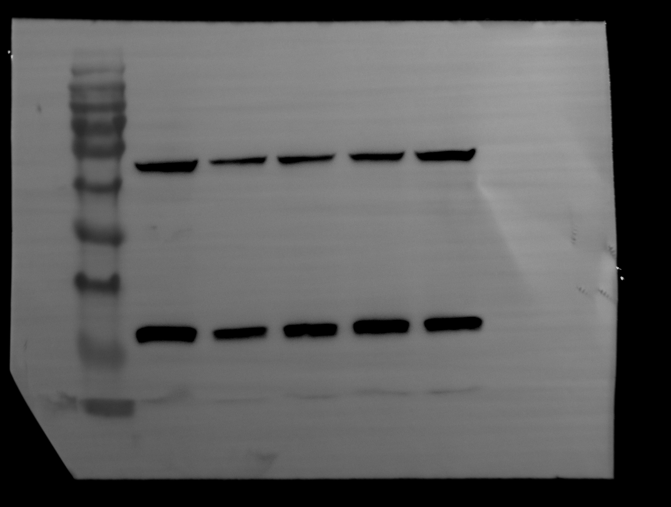

Supplement: Supplementary file 1 [file DataSheet3.ZIP › original data of figure 6/occludin-1.tif]

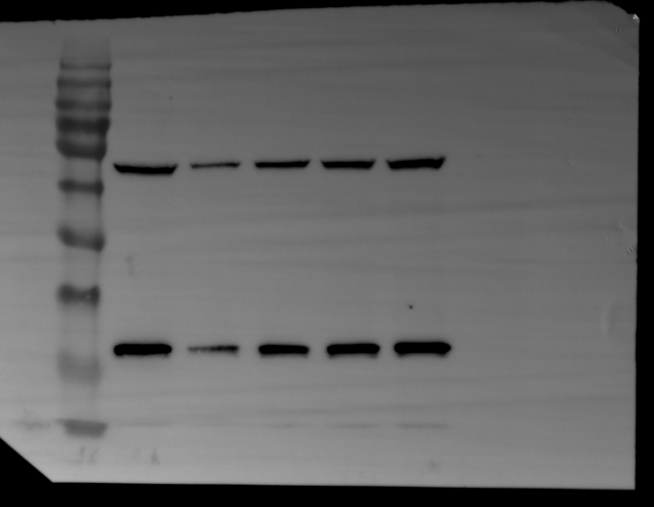

Supplement: Supplementary file 1 [file DataSheet3.ZIP › original data of figure 6/occludin-2.tif]

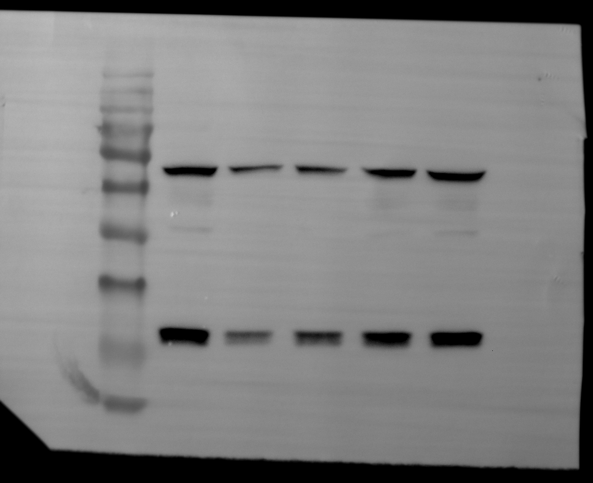

Supplement: Supplementary file 1 [file DataSheet3.ZIP › original data of figure 6/occludin-3.tif]

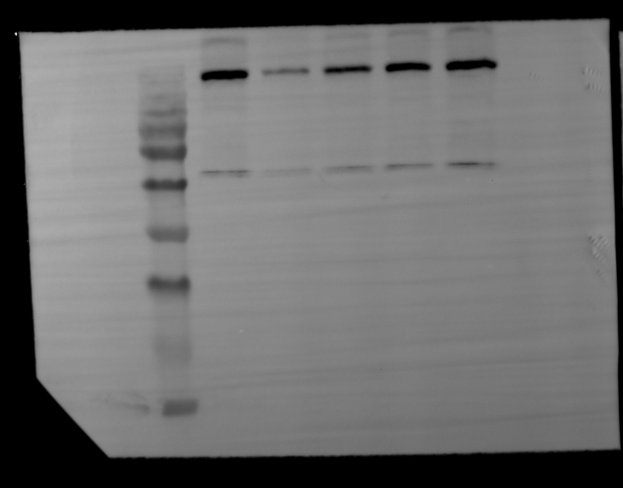

Supplement: Supplementary file 1 [file DataSheet3.ZIP › original data of figure 6/ZO1-1.tif]

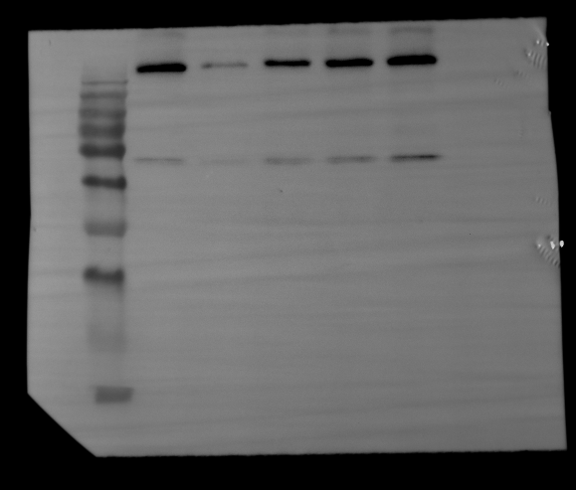

Supplement: Supplementary file 1 [file DataSheet3.ZIP › original data of figure 6/ZO1-2.tif]

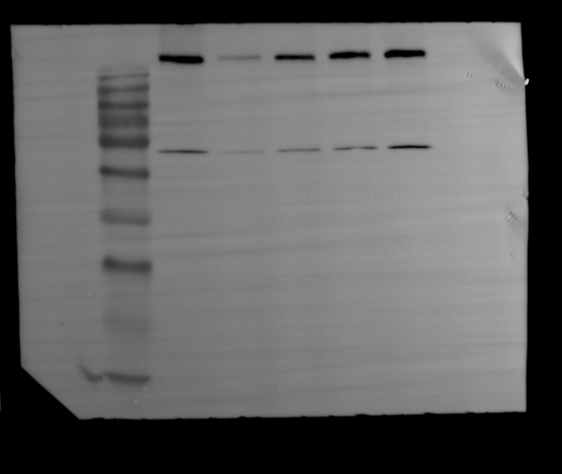

Supplement: Supplementary file 1 [file DataSheet3.ZIP › original data of figure 6/ZO1-3.tif]

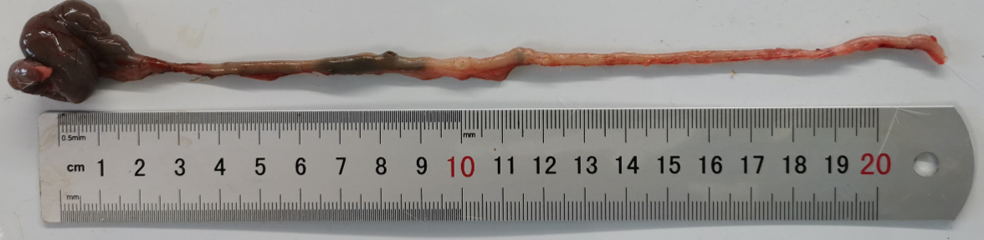

Supplement: Supplementary file 2 [file DataSheet4.ZIP › original data of figure 2-1/Figure 1 CON of colon tissue.tif]

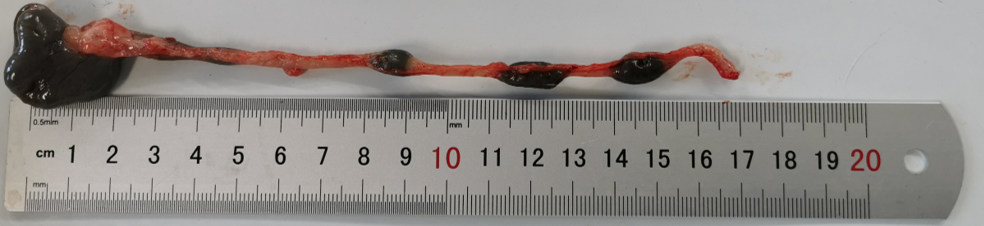

Supplement: Supplementary file 2 [file DataSheet4.ZIP › original data of figure 2-1/Figure 2 DSS of colon tissue.tif]

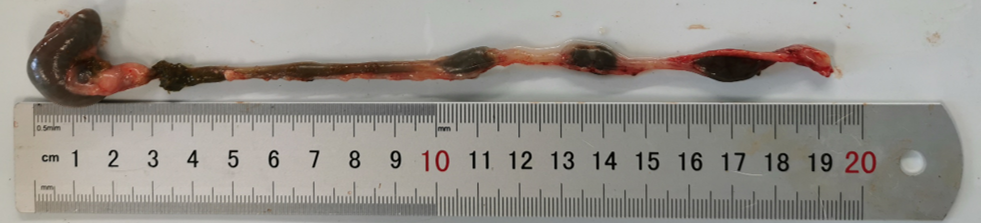

Supplement: Supplementary file 2 [file DataSheet4.ZIP › original data of figure 2-1/Figure 3 LD of colon tissue.tif]

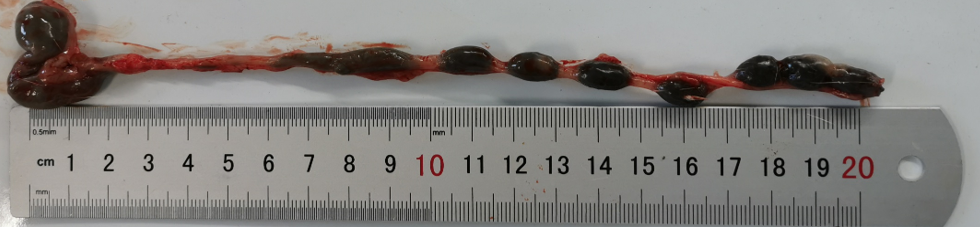

Supplement: Supplementary file 2 [file DataSheet4.ZIP › original data of figure 2-1/Figure 4 MD of colon tissue.tif]

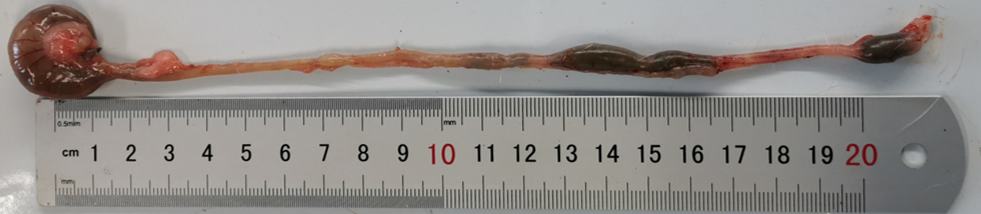

Supplement: Supplementary file 2 [file DataSheet4.ZIP › original data of figure 2-1/Figure 5 HD of colon tissue.tif]

IKB $\alpha$

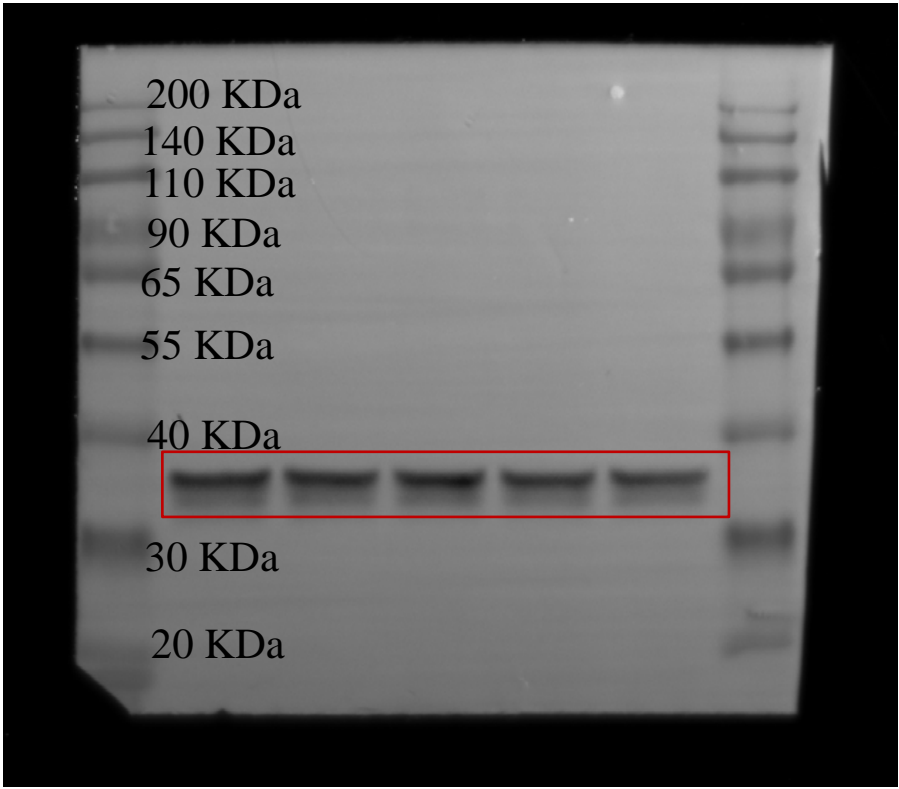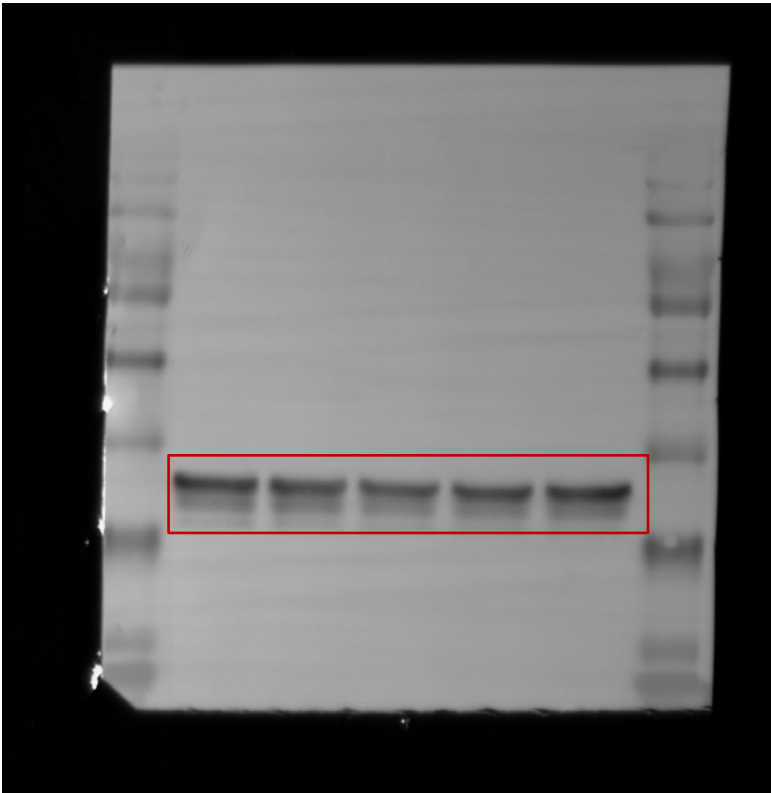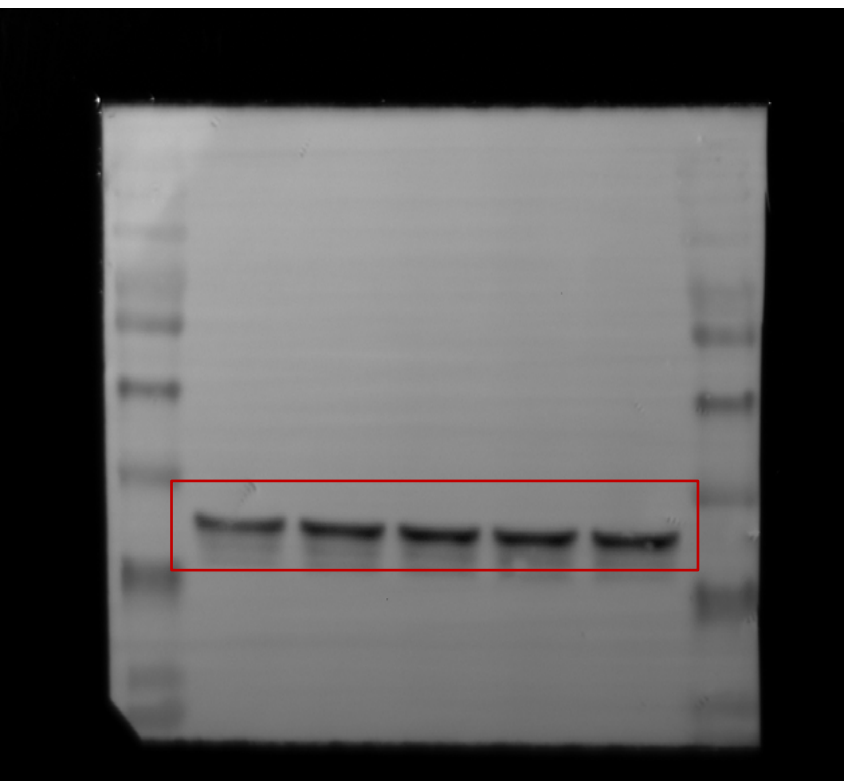

p-IKB $\alpha$

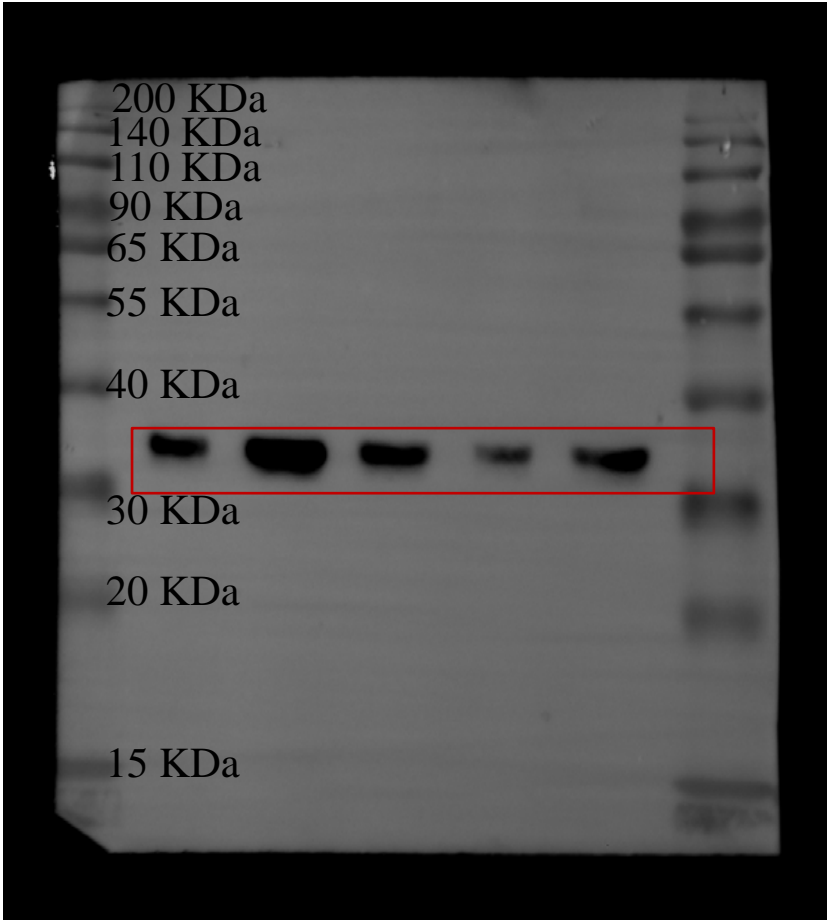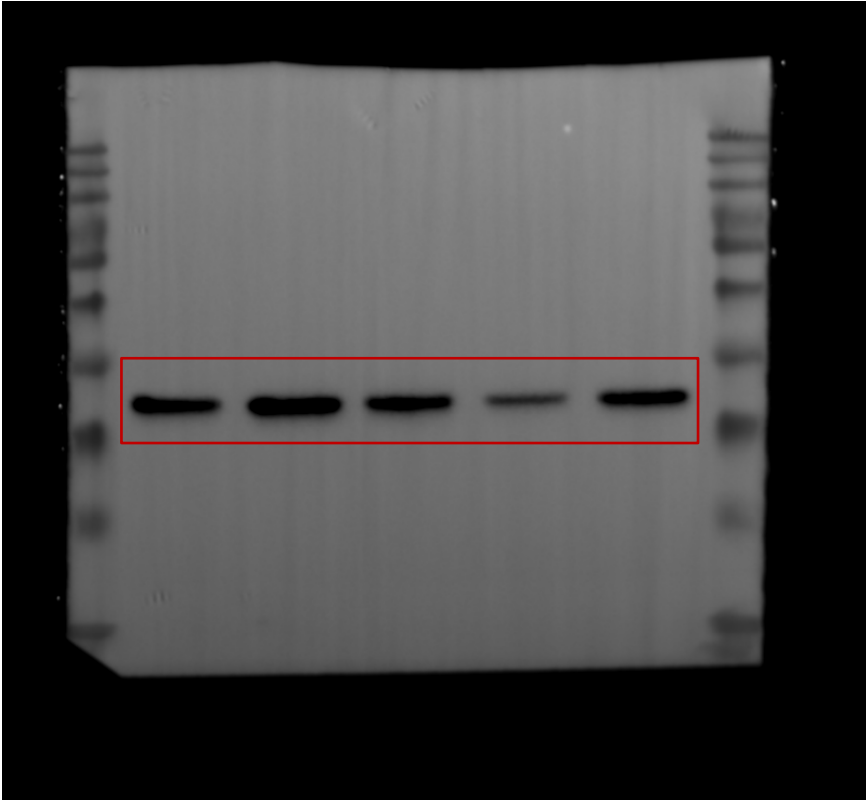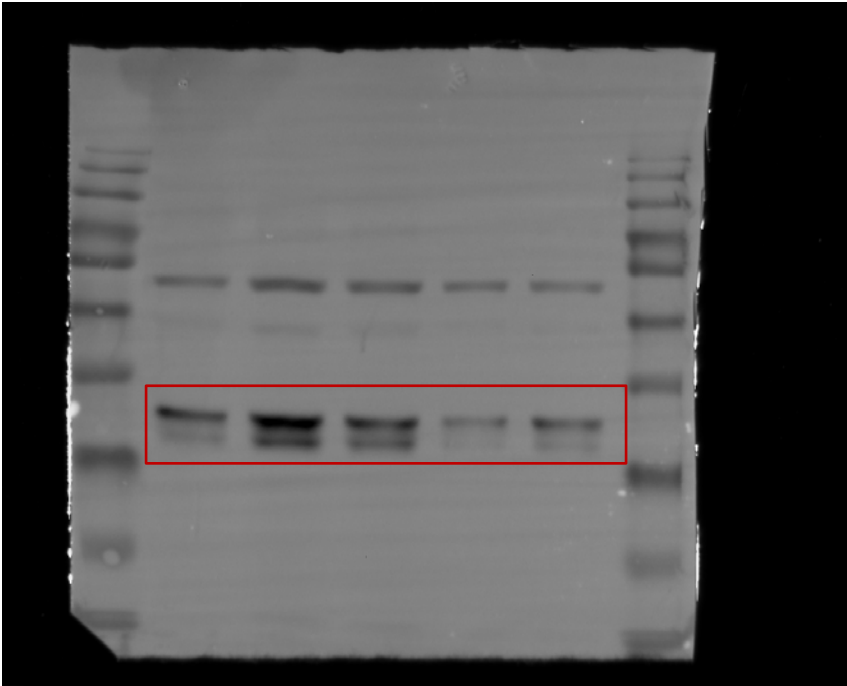

p65

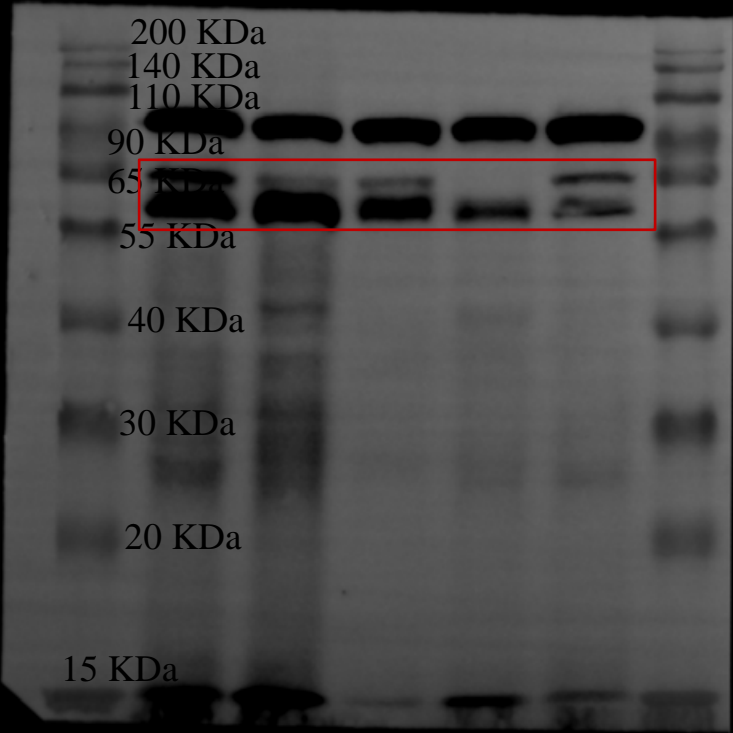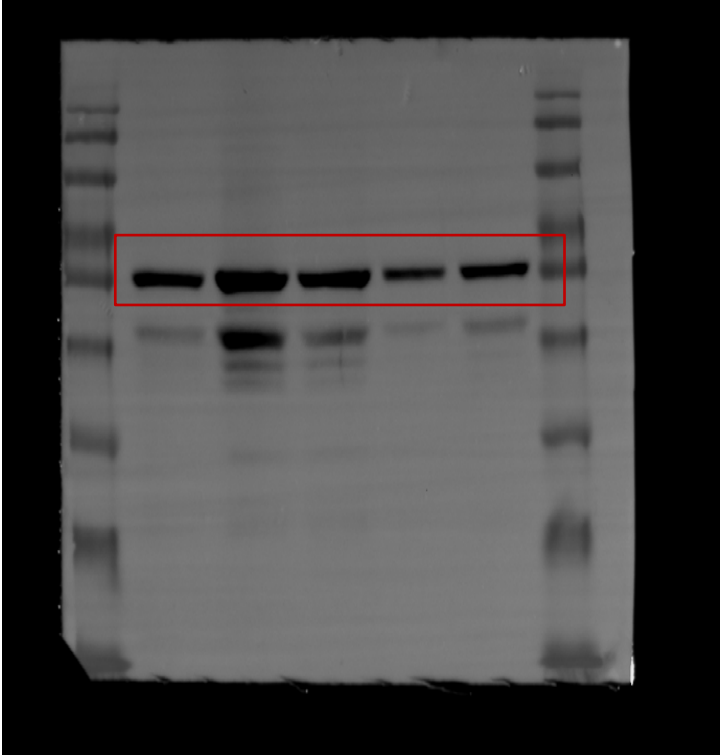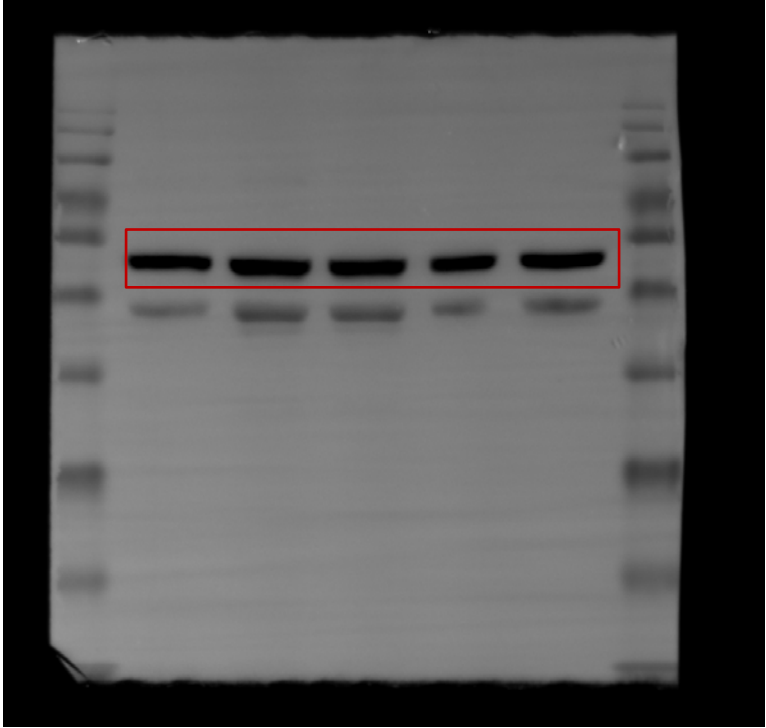

p-p65

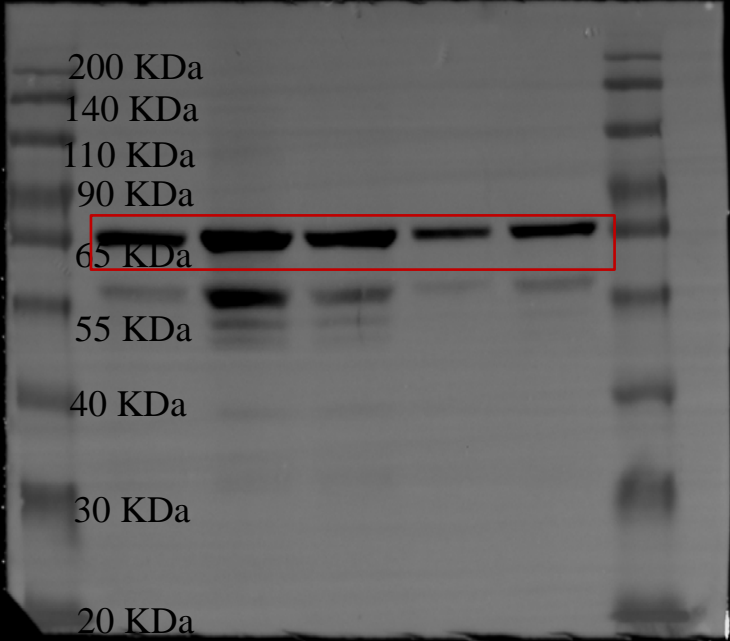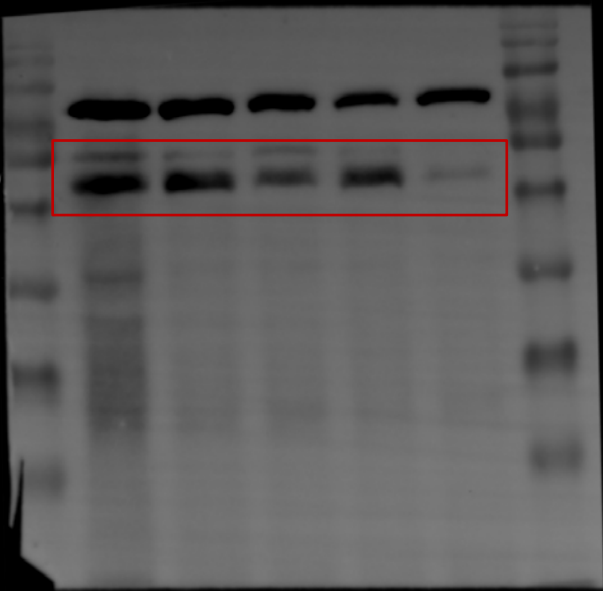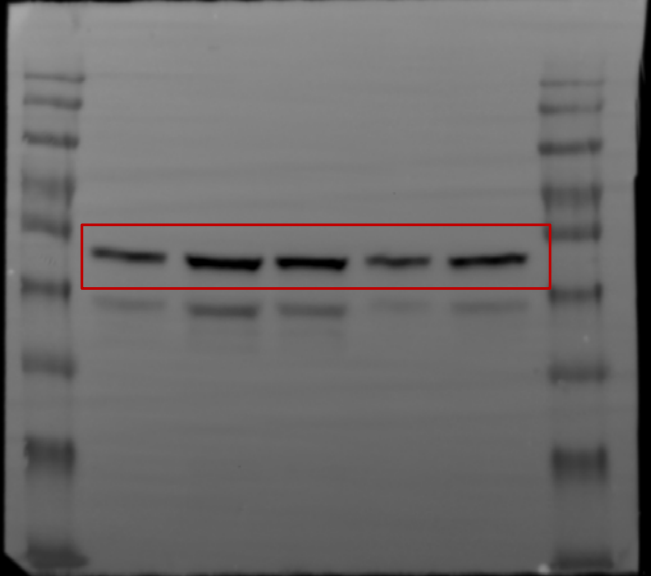

$\beta$ -Actin

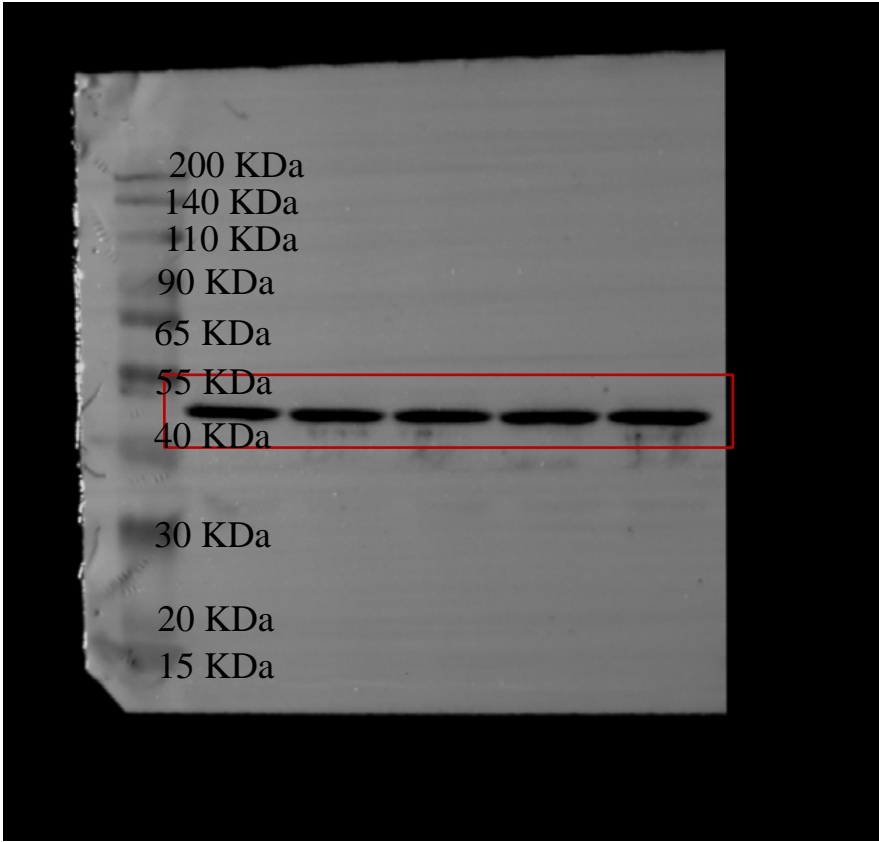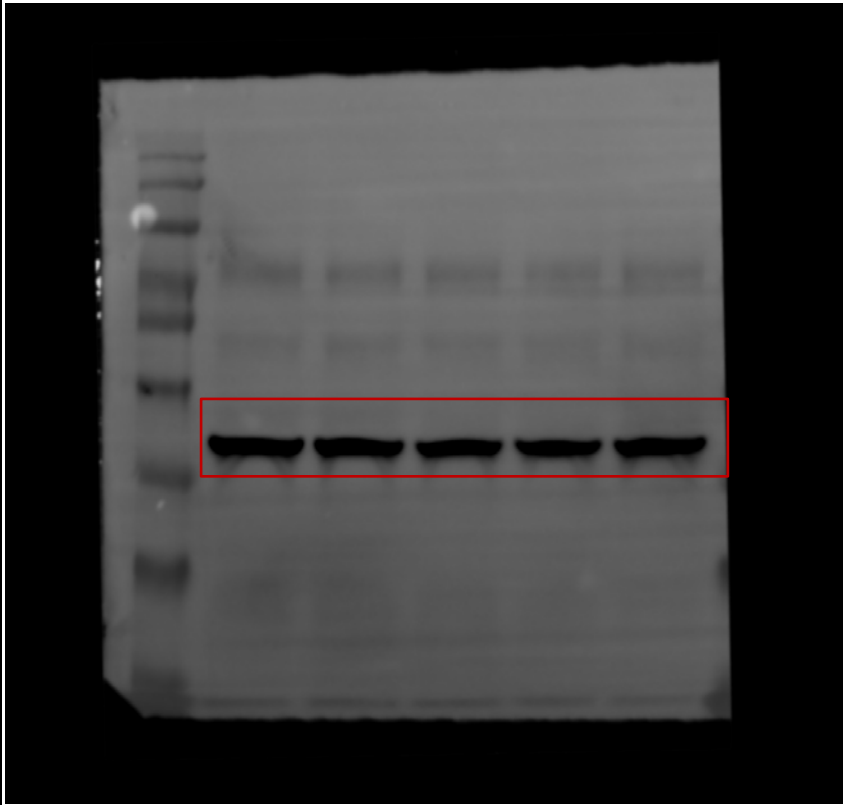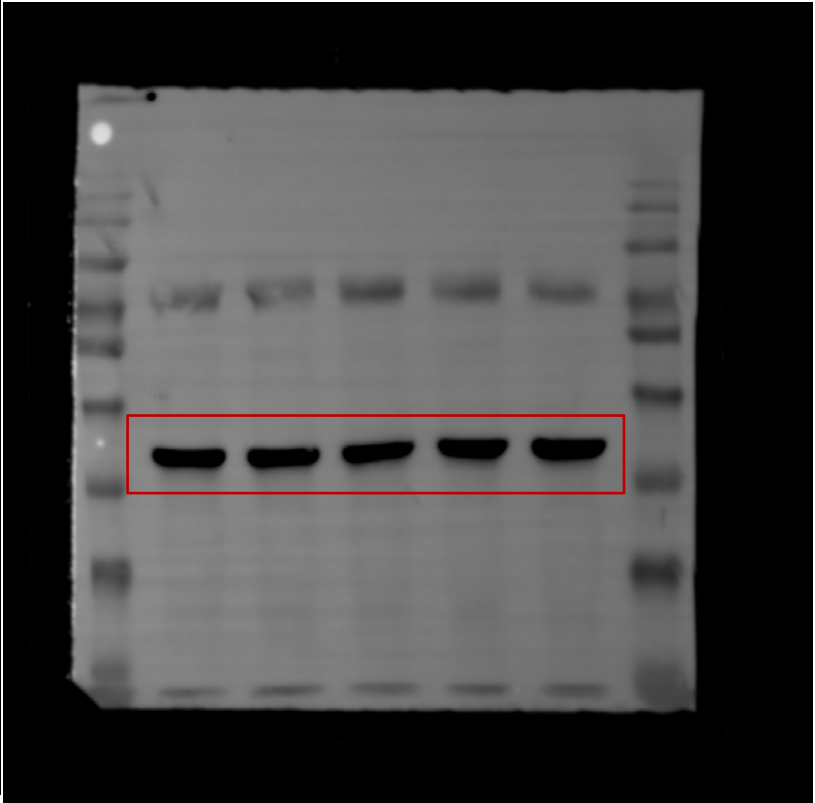

Supplement: Supplementary file 3 [file DataSheet1.ZIP › original data of figure 4/1. WB image of NF-κB.pdf]

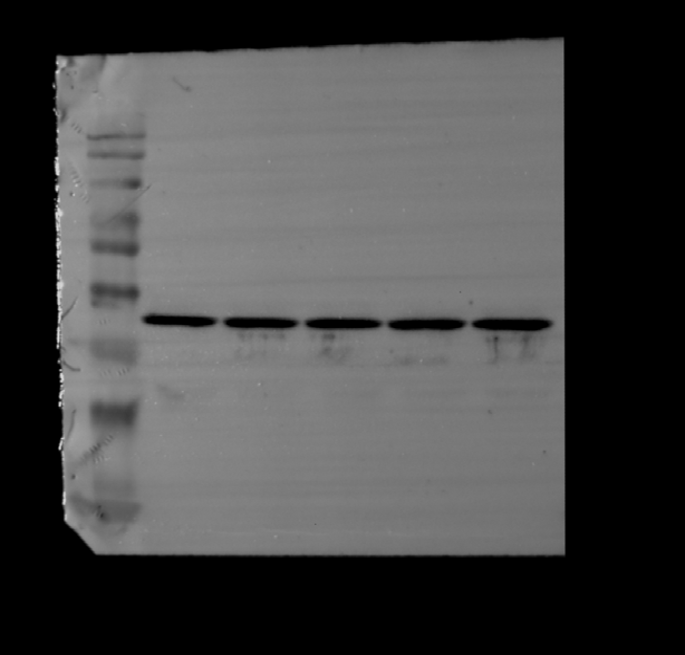

Supplement: Supplementary file 3 [file DataSheet1.ZIP › original data of figure 4/Actin-1.tif]

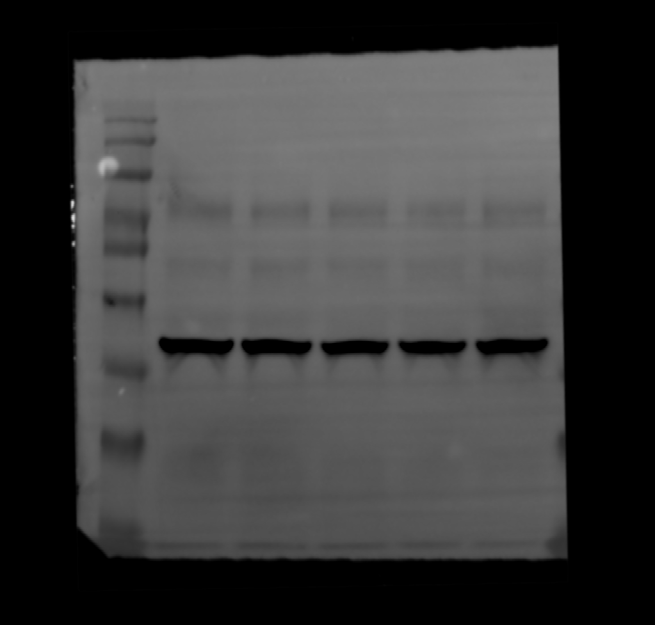

Supplement: Supplementary file 3 [file DataSheet1.ZIP › original data of figure 4/Actin-2.tif]

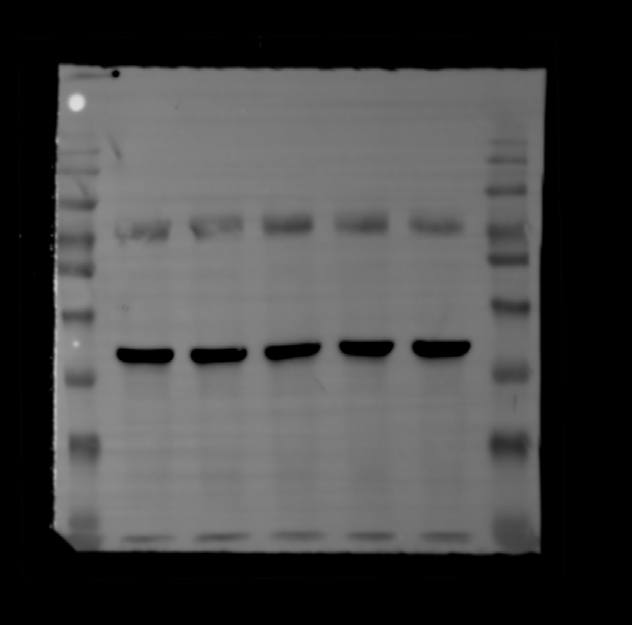

Supplement: Supplementary file 3 [file DataSheet1.ZIP › original data of figure 4/Actin-3.png.tif]

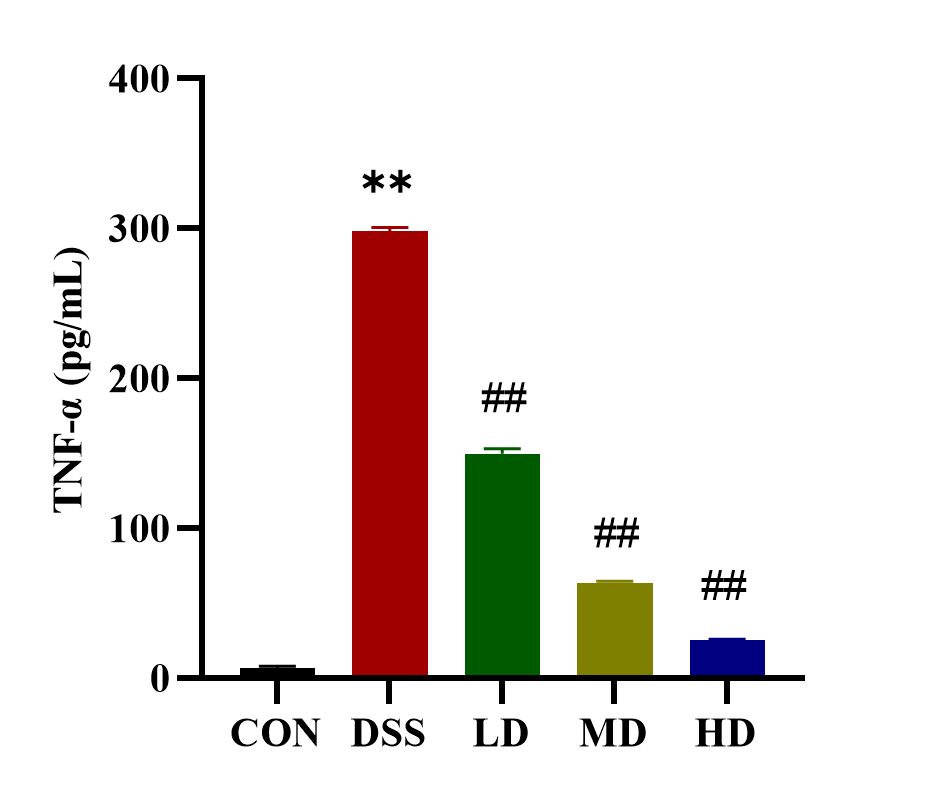

Supplement: Supplementary file 3 [file DataSheet1.ZIP › original data of figure 4/Figure1 rat TNF-α.tif]

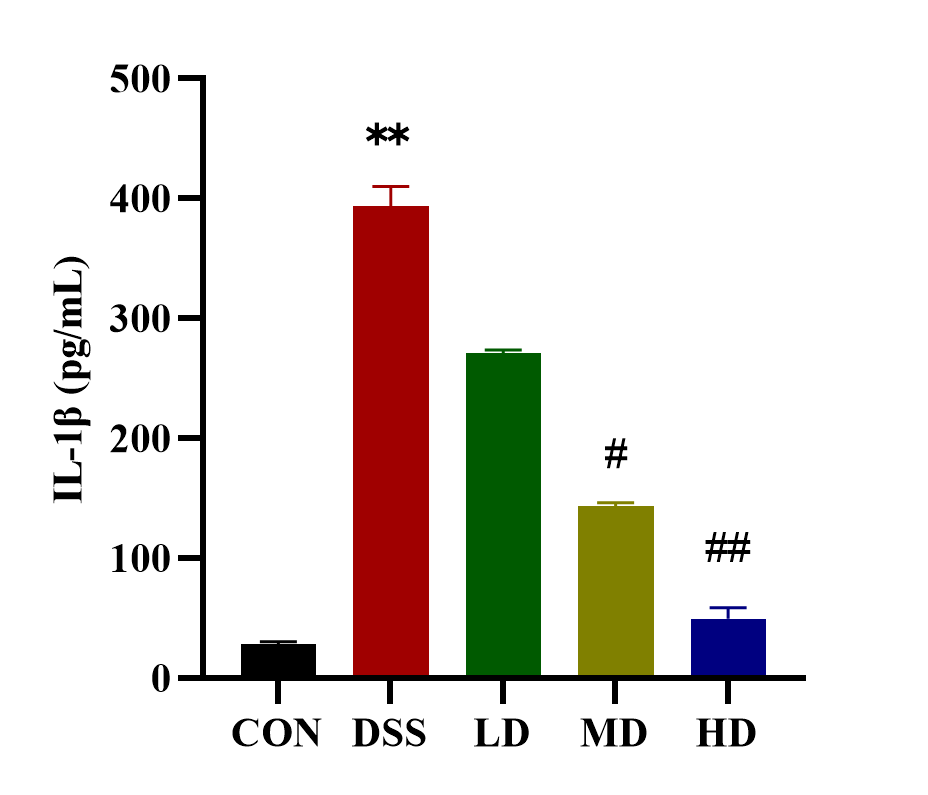

Supplement: Supplementary file 3 [file DataSheet1.ZIP › original data of figure 4/Figure2 rat IL-1beta.tif]

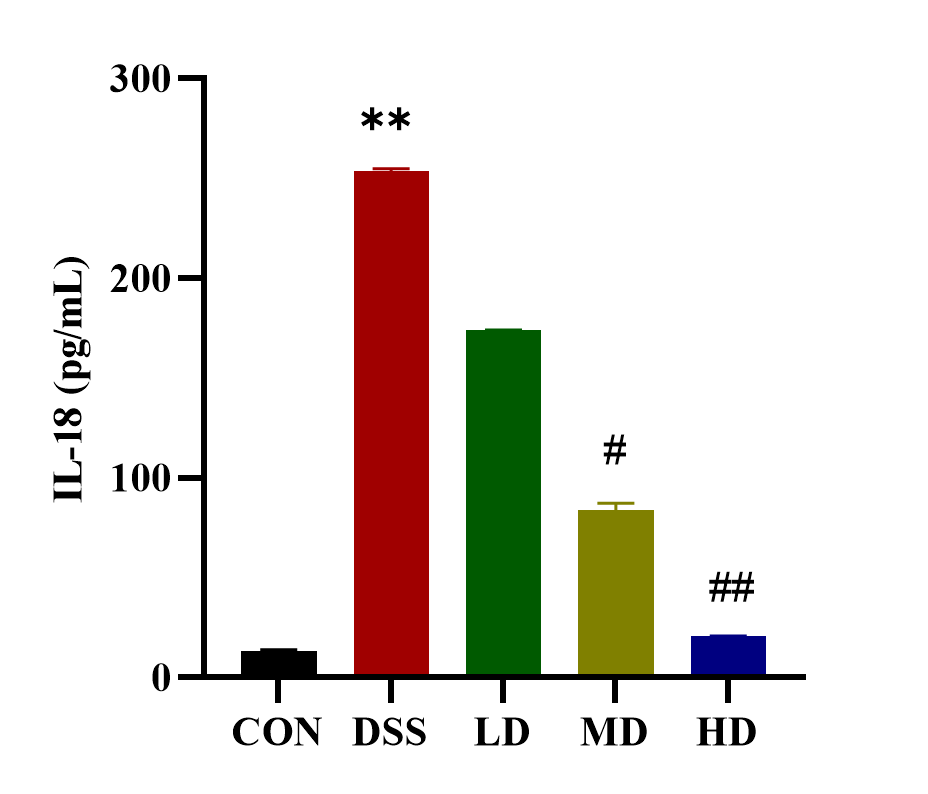

Supplement: Supplementary file 3 [file DataSheet1.ZIP › original data of figure 4/Figure3 rat IL-18.tif]

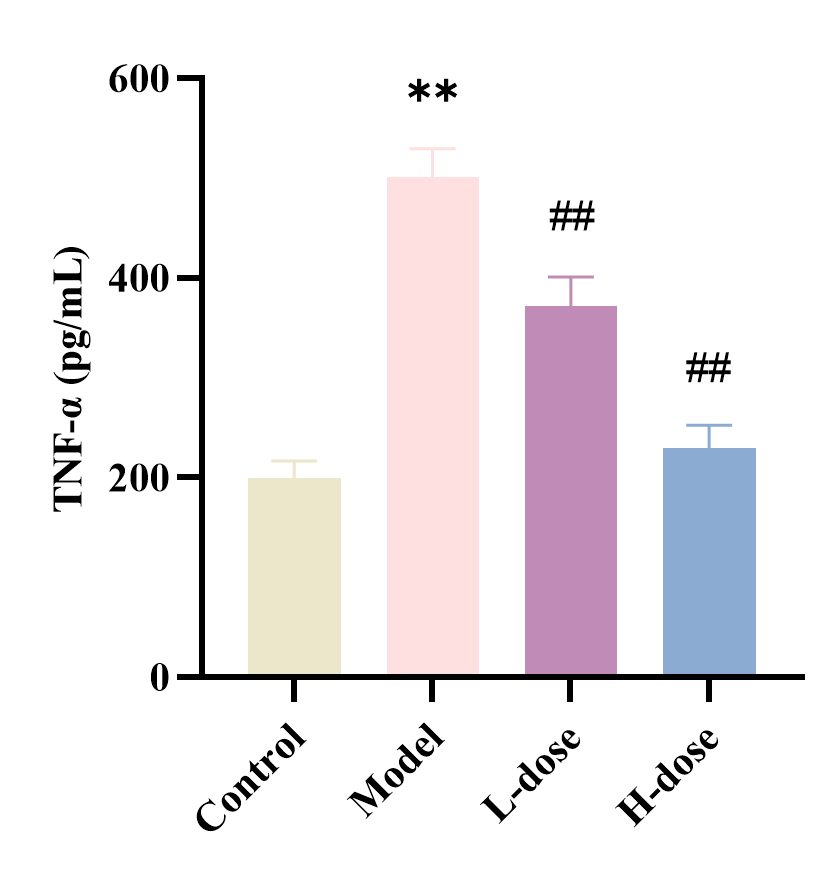

Supplement: Supplementary file 3 [file DataSheet1.ZIP › original data of figure 4/Figure4 cell TNF-α.tif]

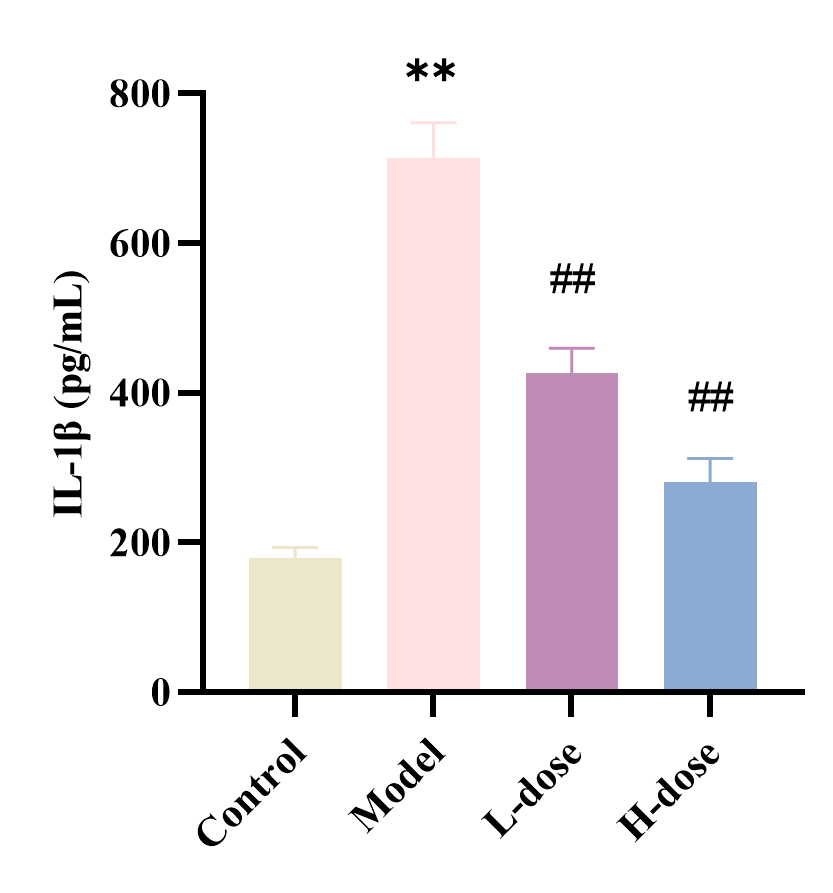

Supplement: Supplementary file 3 [file DataSheet1.ZIP › original data of figure 4/Figure5 cell IL-1β.tif]

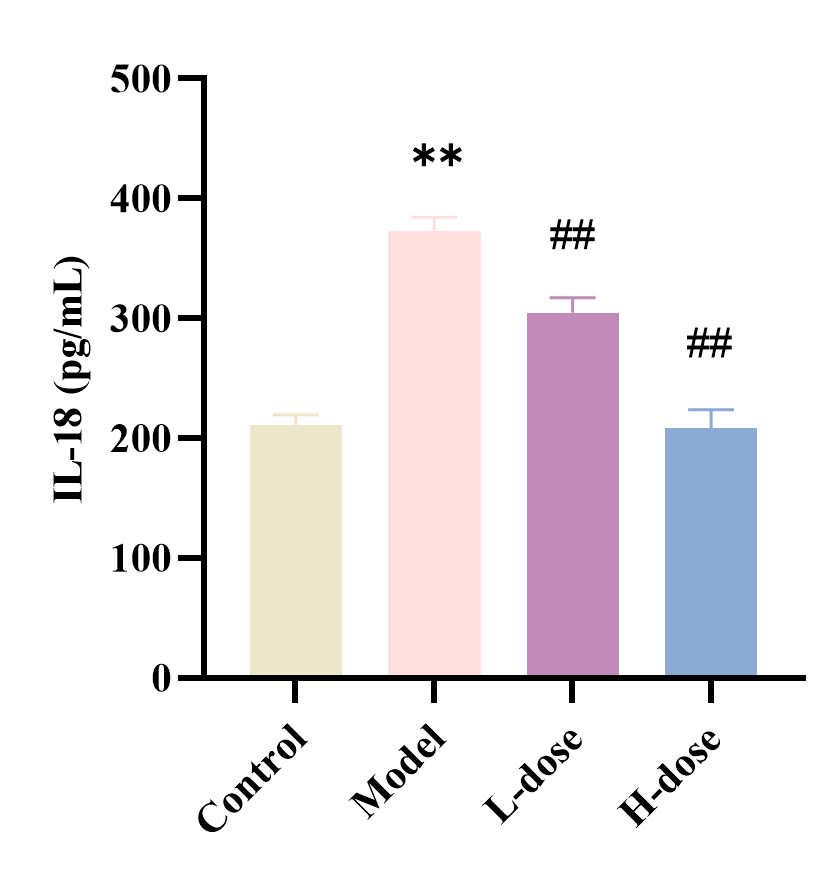

Supplement: Supplementary file 3 [file DataSheet1.ZIP › original data of figure 4/Figure6 cell IL-18.tif]

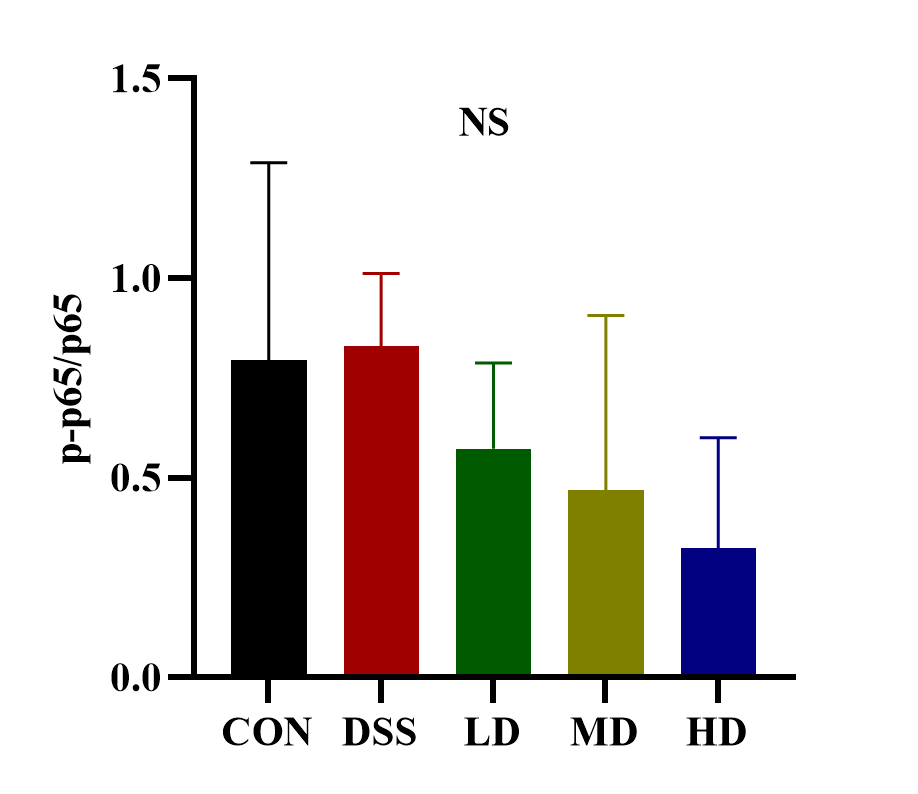

Supplement: Supplementary file 3 [file DataSheet1.ZIP › original data of figure 4/Figure7 p-p65_p65_ratio.tif]

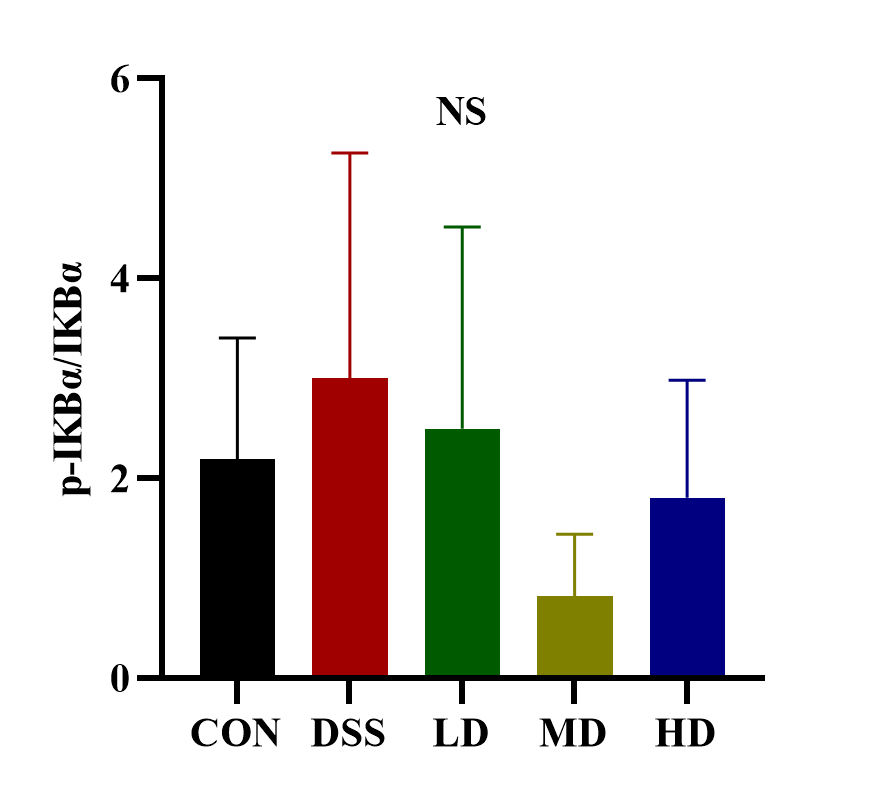

Supplement: Supplementary file 3 [file DataSheet1.ZIP › original data of figure 4/Figure8 p-IKBa_IKBa_ratio.tif]

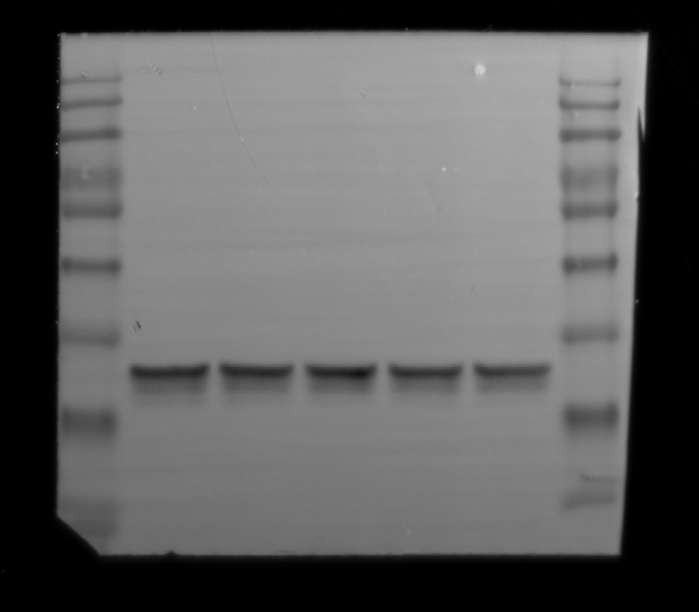

Supplement: Supplementary file 3 [file DataSheet1.ZIP › original data of figure 4/IKBα-1.tif]

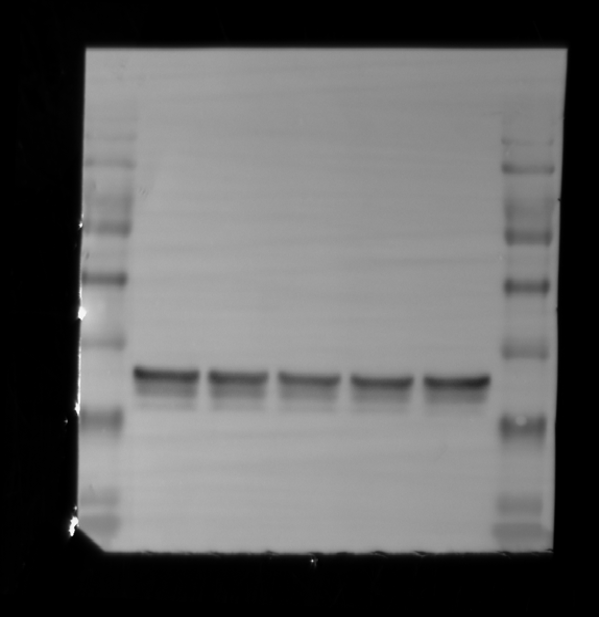

Supplement: Supplementary file 3 [file DataSheet1.ZIP › original data of figure 4/IKBα-2.tif]

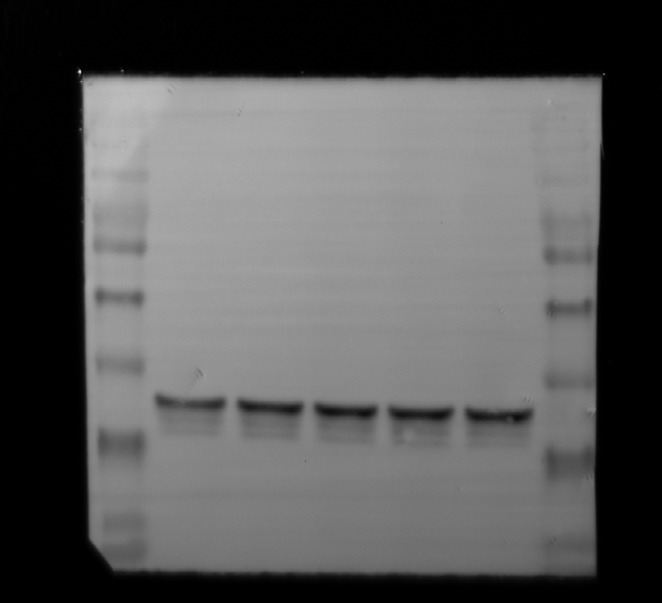

Supplement: Supplementary file 3 [file DataSheet1.ZIP › original data of figure 4/IKBα-3.tif]

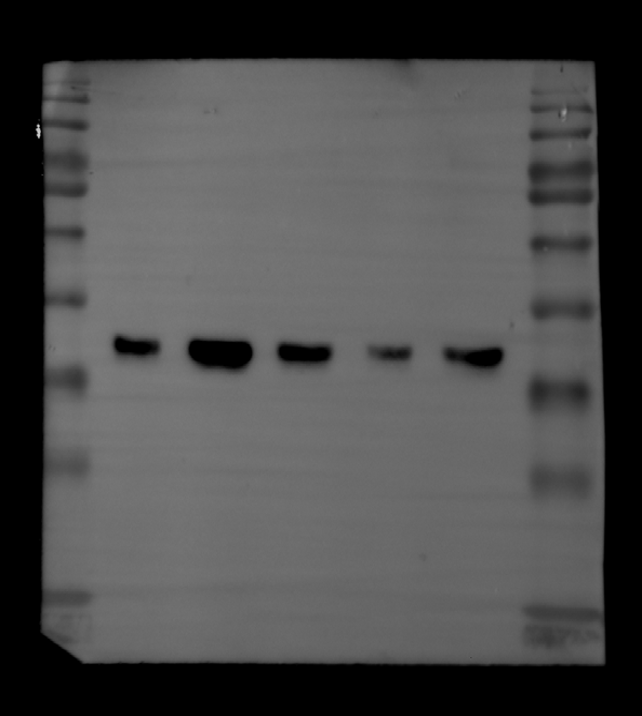

Supplement: Supplementary file 3 [file DataSheet1.ZIP › original data of figure 4/p-IKBα-1.tif]

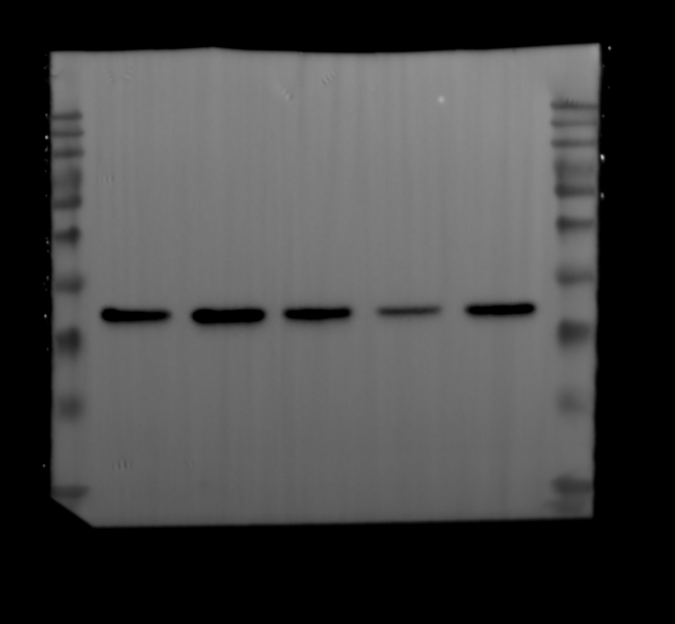

Supplement: Supplementary file 3 [file DataSheet1.ZIP › original data of figure 4/p-IKBα-2.tif]

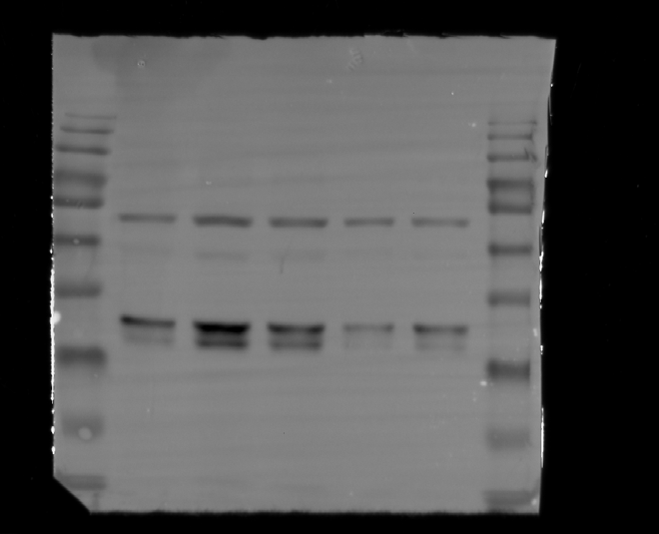

Supplement: Supplementary file 3 [file DataSheet1.ZIP › original data of figure 4/p-IKBα-3.tif]

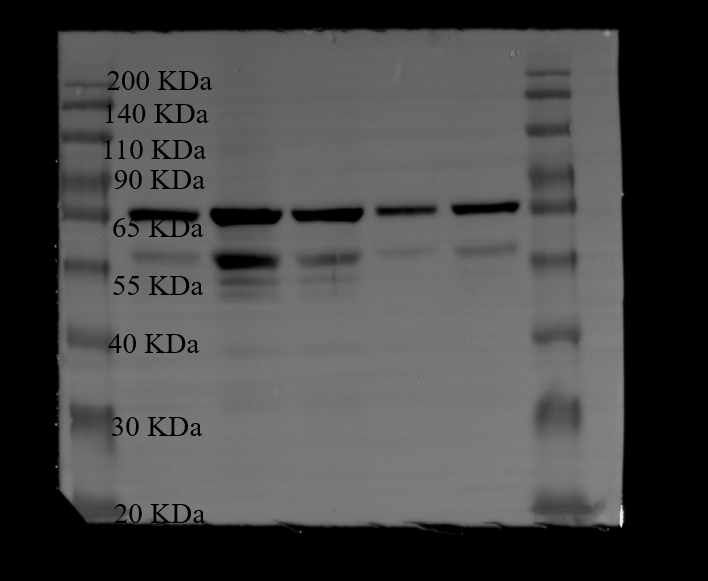

Supplement: Supplementary file 3 [file DataSheet1.ZIP › original data of figure 4/p-p65-1.tif]

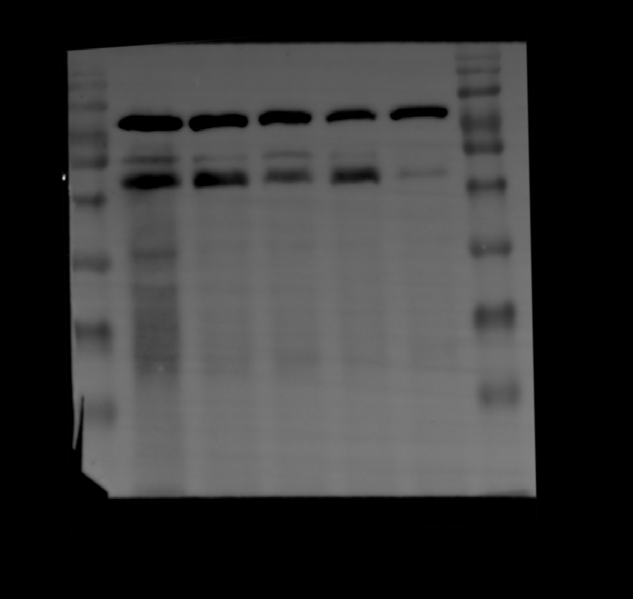

Supplement: Supplementary file 3 [file DataSheet1.ZIP › original data of figure 4/p-p65-2.tif]

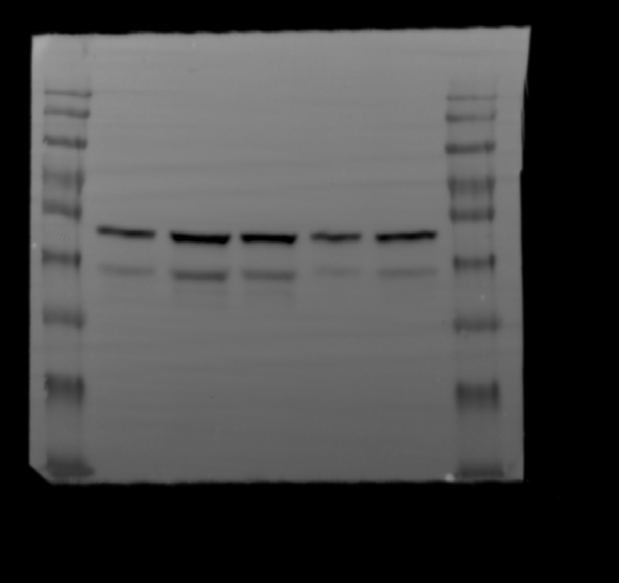

Supplement: Supplementary file 3 [file DataSheet1.ZIP › original data of figure 4/p-p65-3.tif]

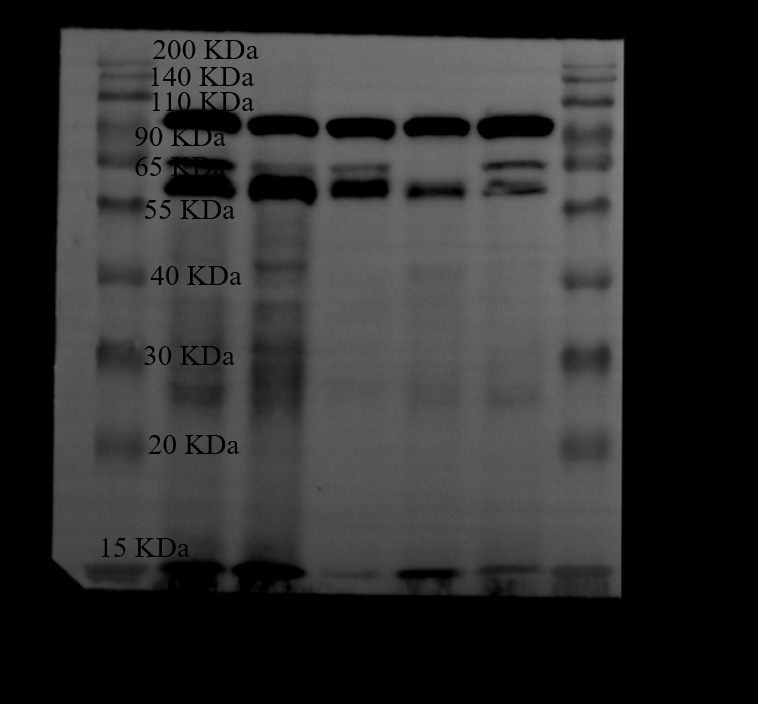

Supplement: Supplementary file 3 [file DataSheet1.ZIP › original data of figure 4/p65-1.tif]

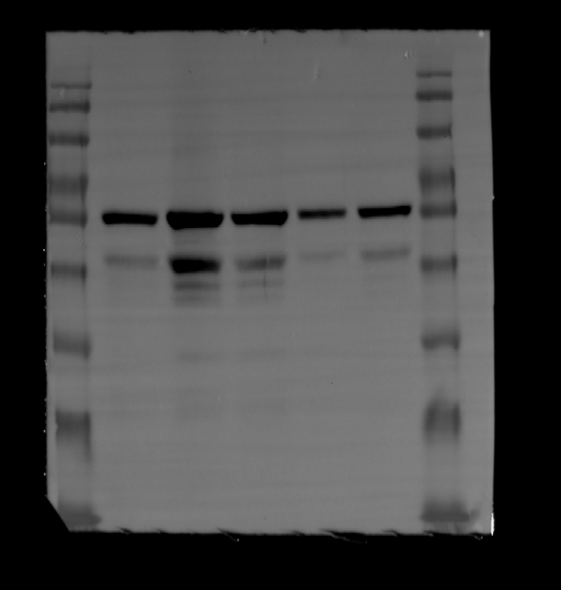

Supplement: Supplementary file 3 [file DataSheet1.ZIP › original data of figure 4/p65-2.tif]

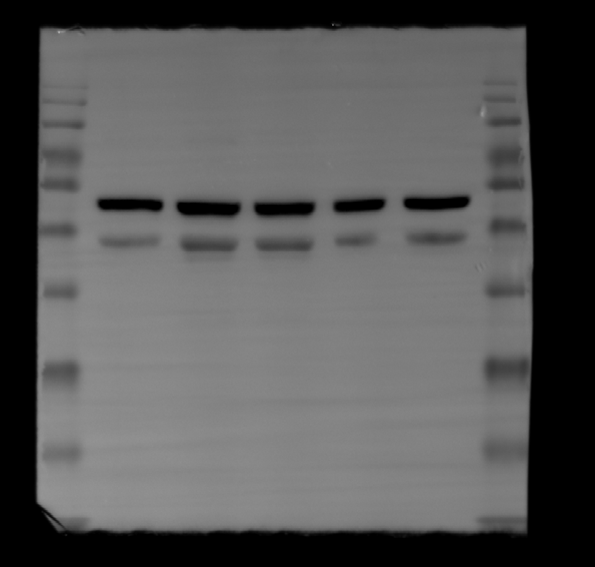

Supplement: Supplementary file 3 [file DataSheet1.ZIP › original data of figure 4/p65-3.tif]

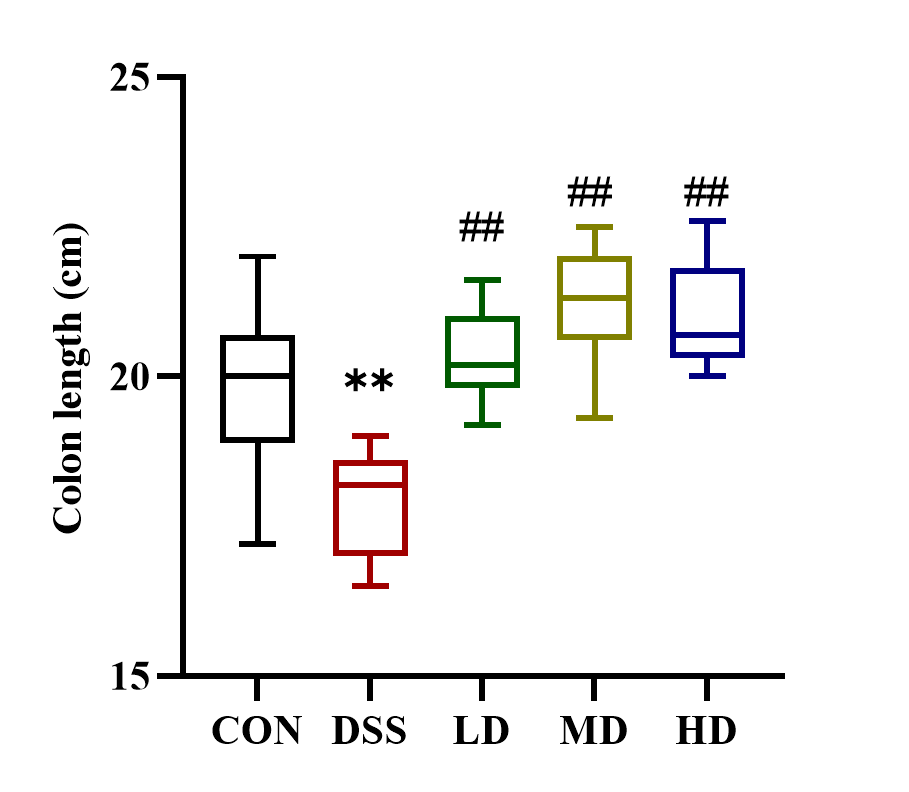

Supplement: Supplementary file 4 [file DataSheet6.ZIP › original data of figure 2-2/Colon length.tif]

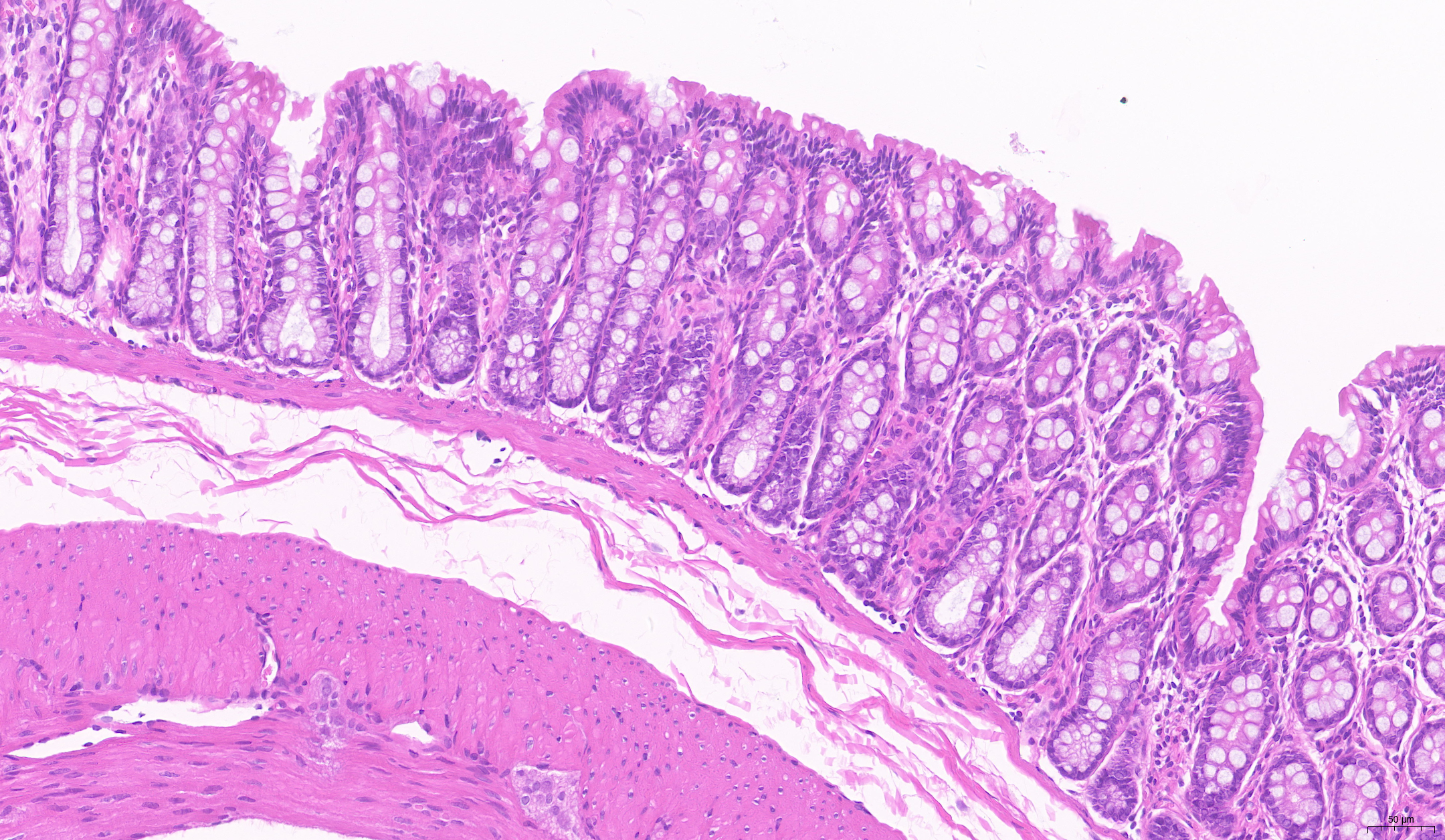

Supplement: Supplementary file 4 [file DataSheet6.ZIP › original data of figure 2-2/Figure1 CON of HE_20.0x.tif]

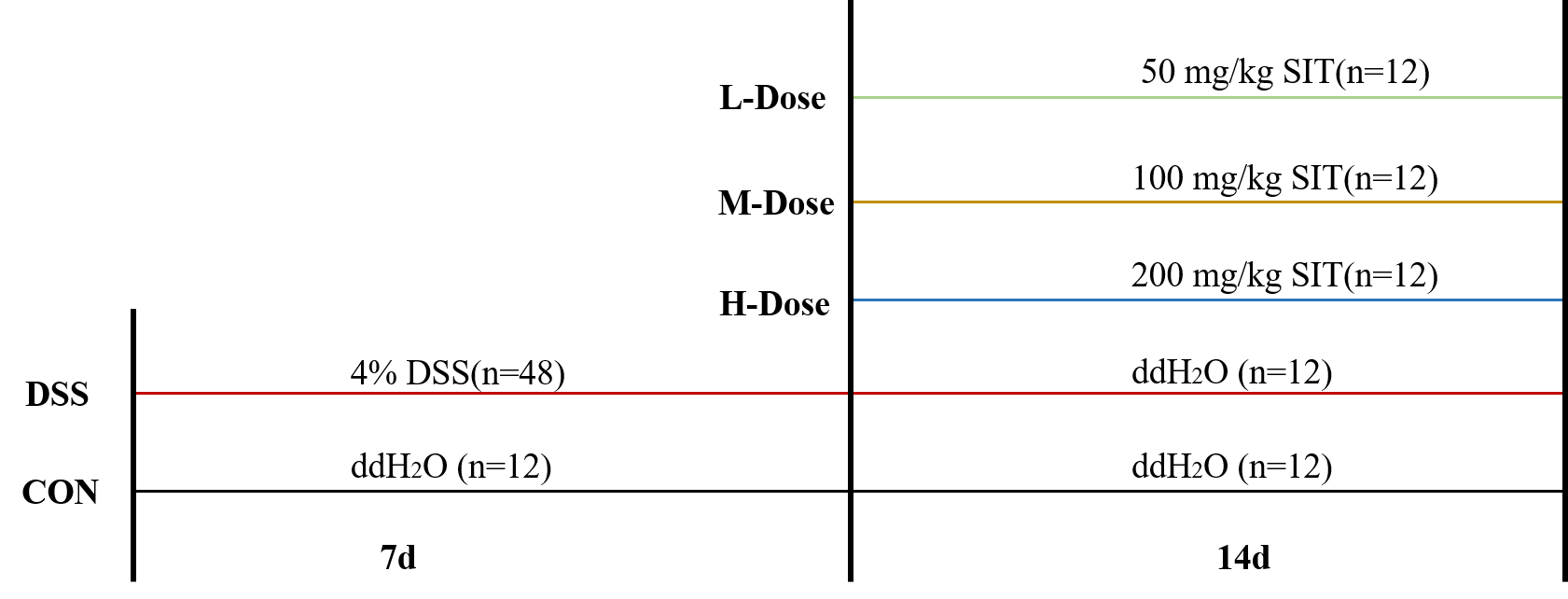

Supplement: Supplementary file 4 [file DataSheet6.ZIP › original data of figure 2-2/Figure10 study design.tif]

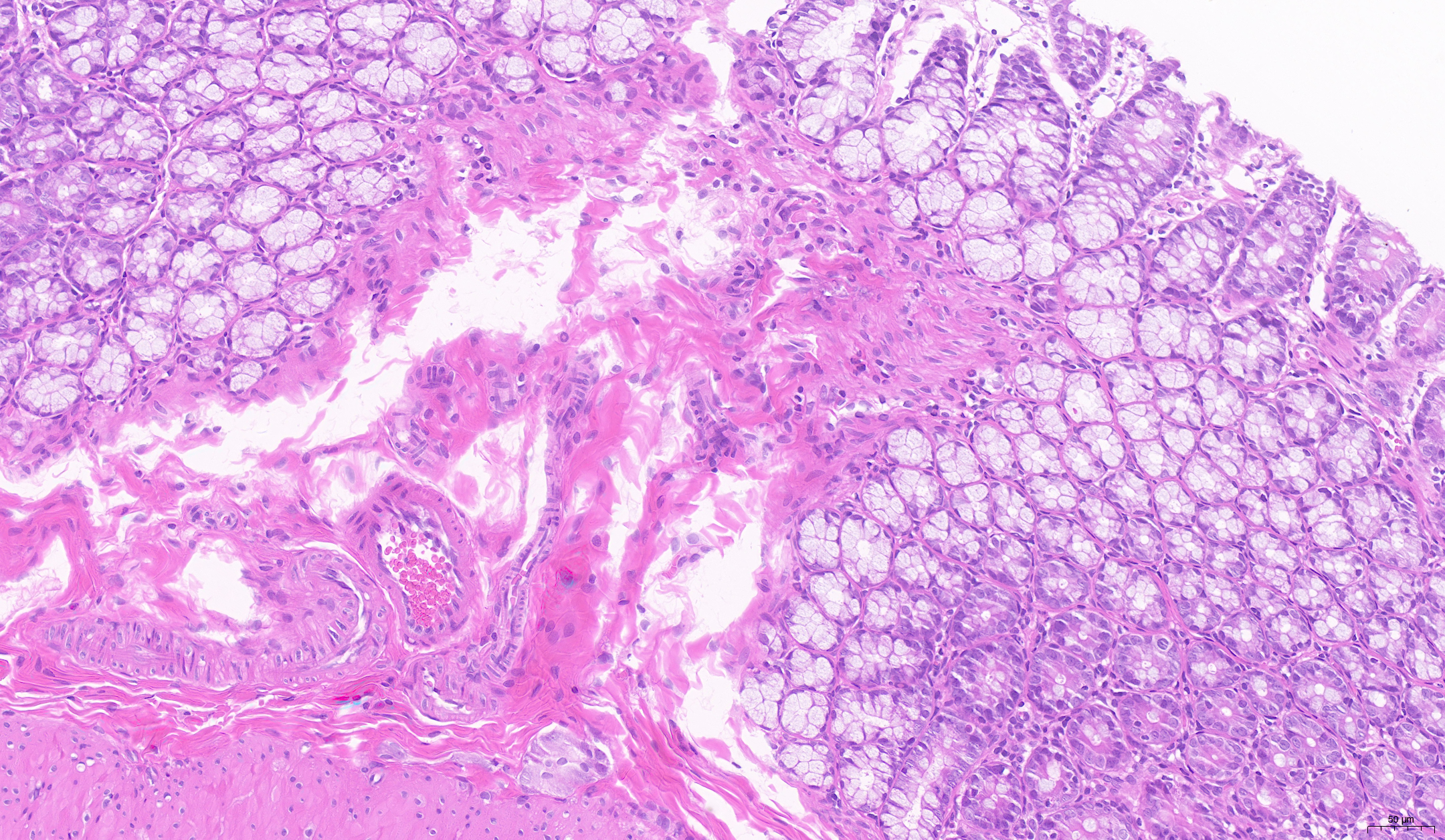

Supplement: Supplementary file 4 [file DataSheet6.ZIP › original data of figure 2-2/Figure2 DSS of HE_20.0x.tif]

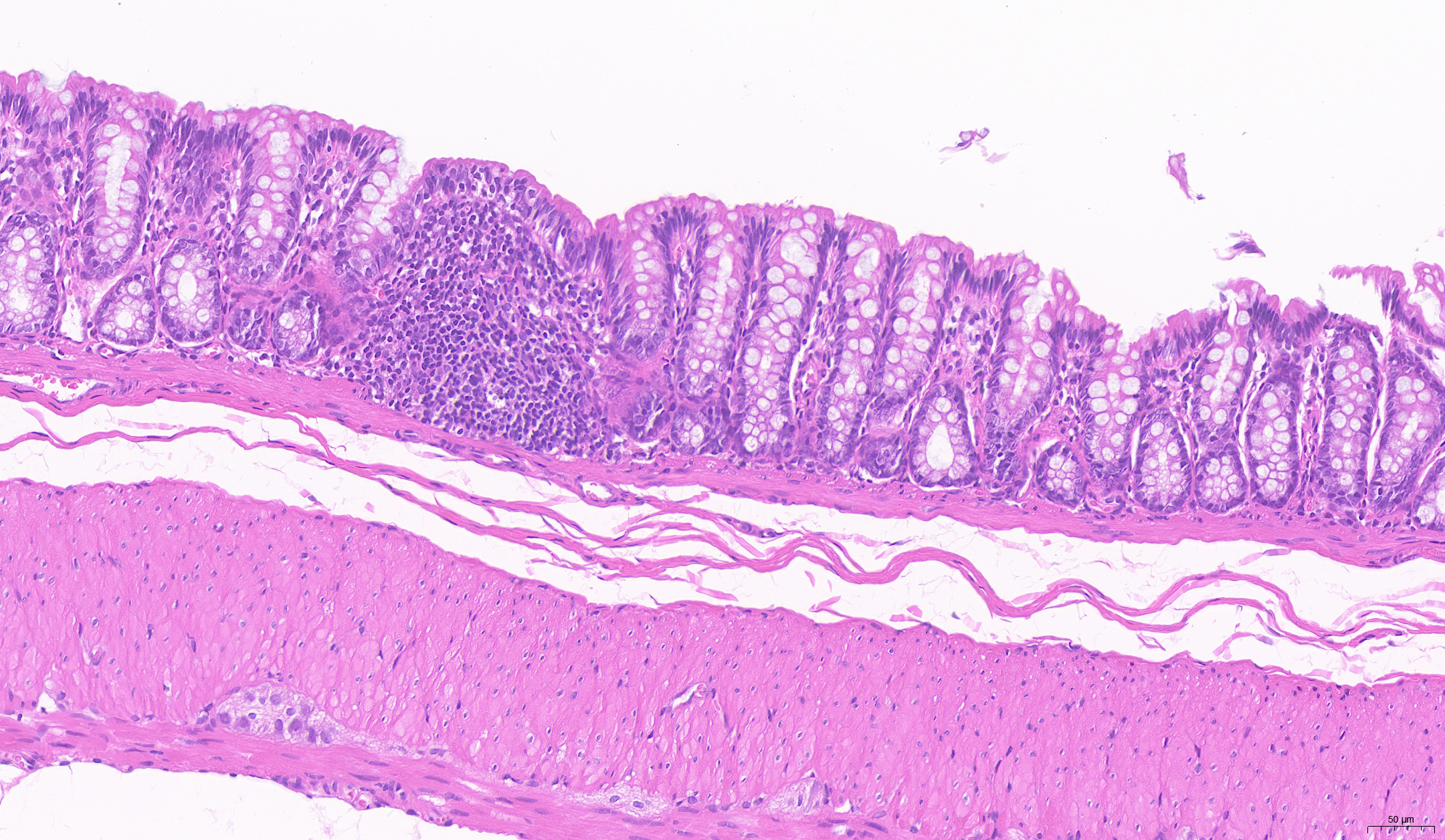

Supplement: Supplementary file 4 [file DataSheet6.ZIP › original data of figure 2-2/Figure3 LD of HE_20.0x.tif]

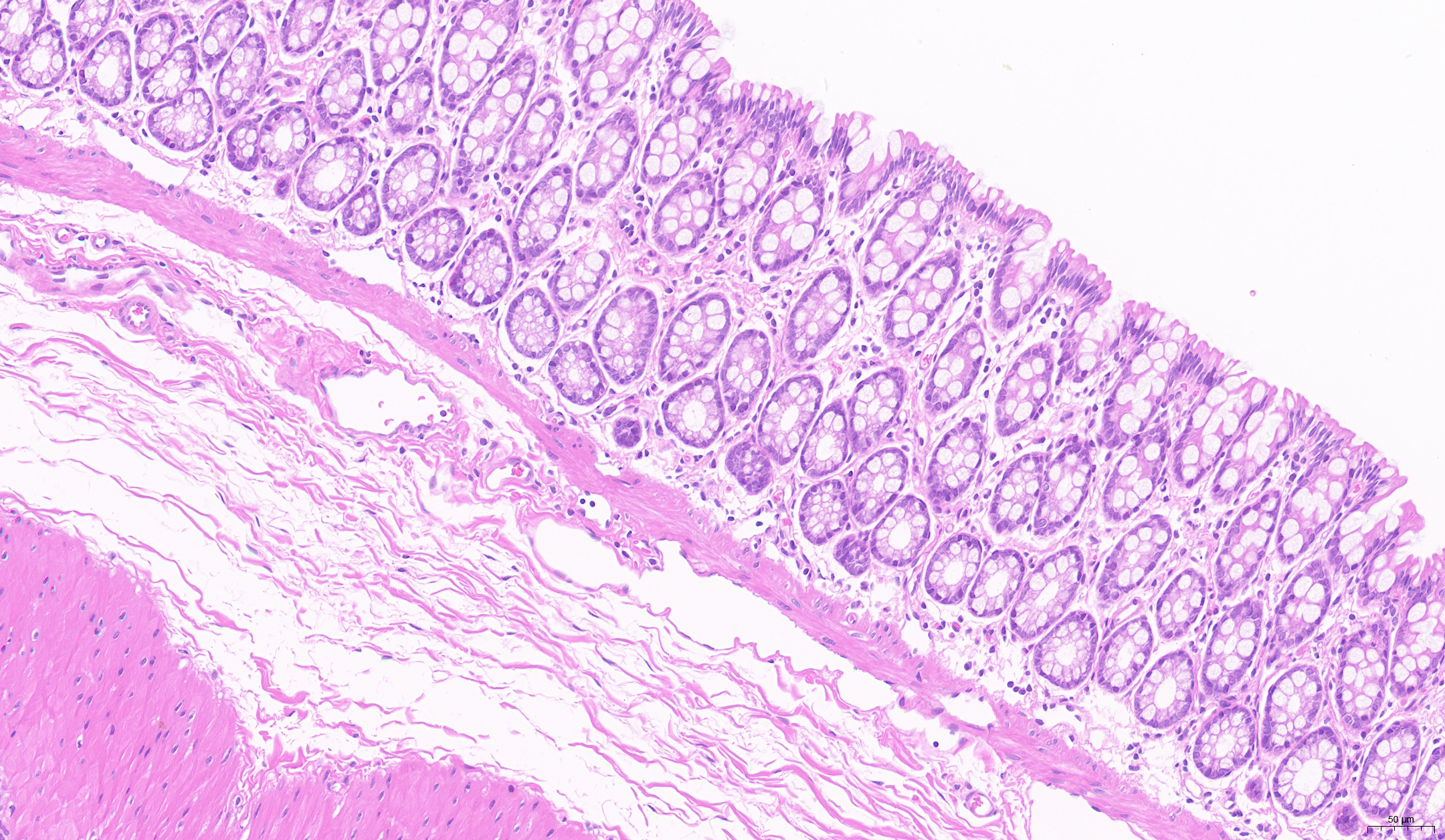

Supplement: Supplementary file 4 [file DataSheet6.ZIP › original data of figure 2-2/Figure4 MD of HE_20.0x.tif]

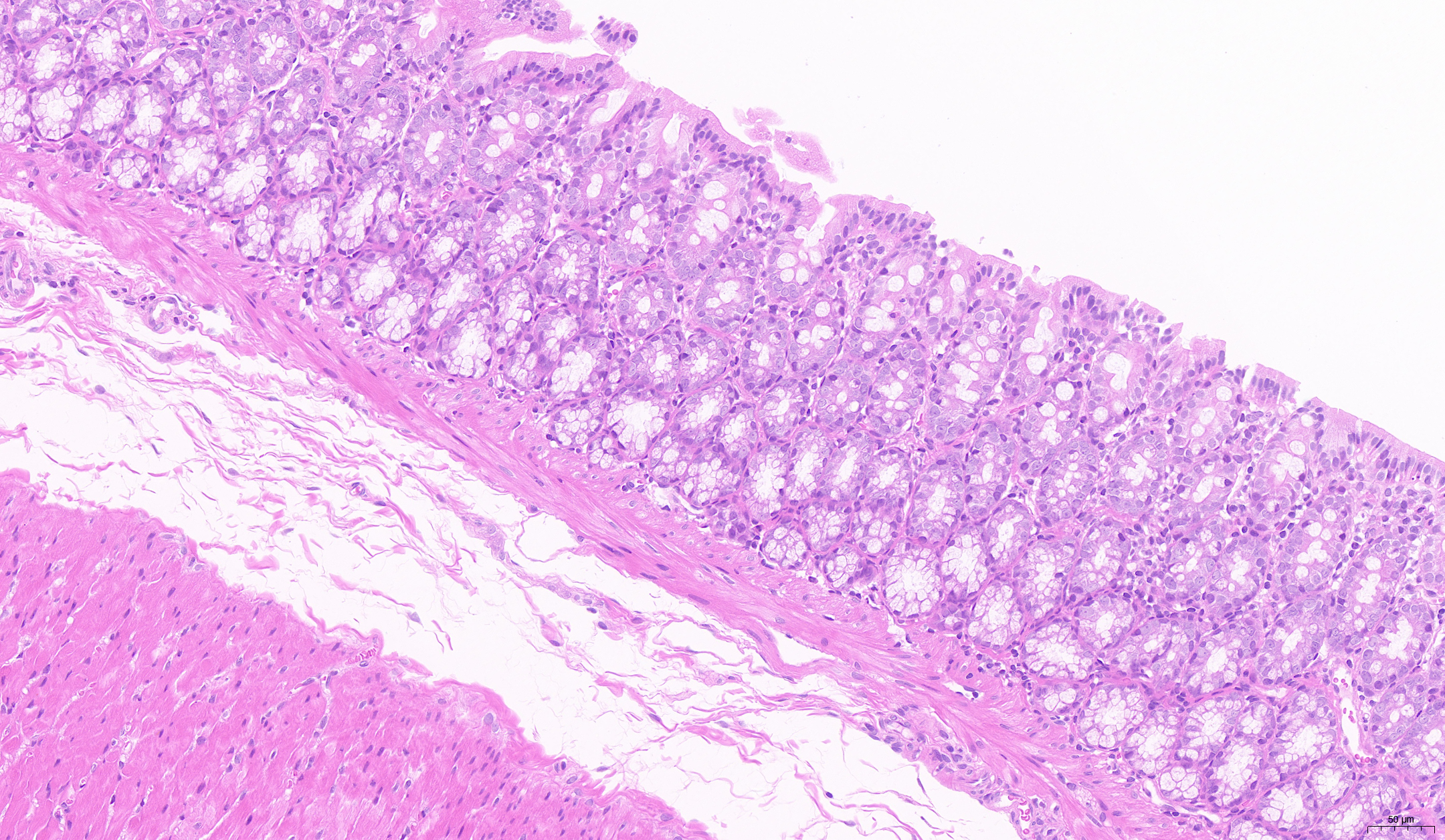

Supplement: Supplementary file 4 [file DataSheet6.ZIP › original data of figure 2-2/Figure5 HD of HE_20.0x.tif]

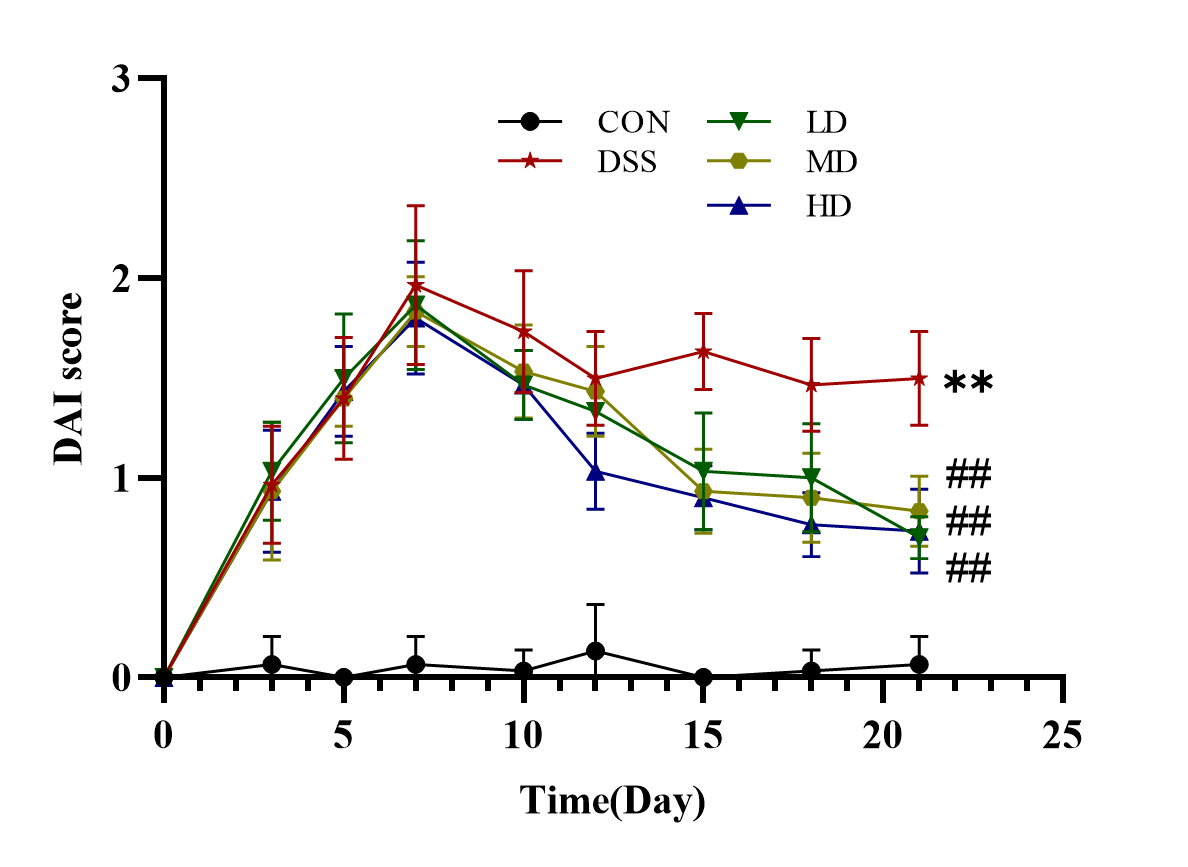

Supplement: Supplementary file 4 [file DataSheet6.ZIP › original data of figure 2-2/Figure7 DAI.tif]

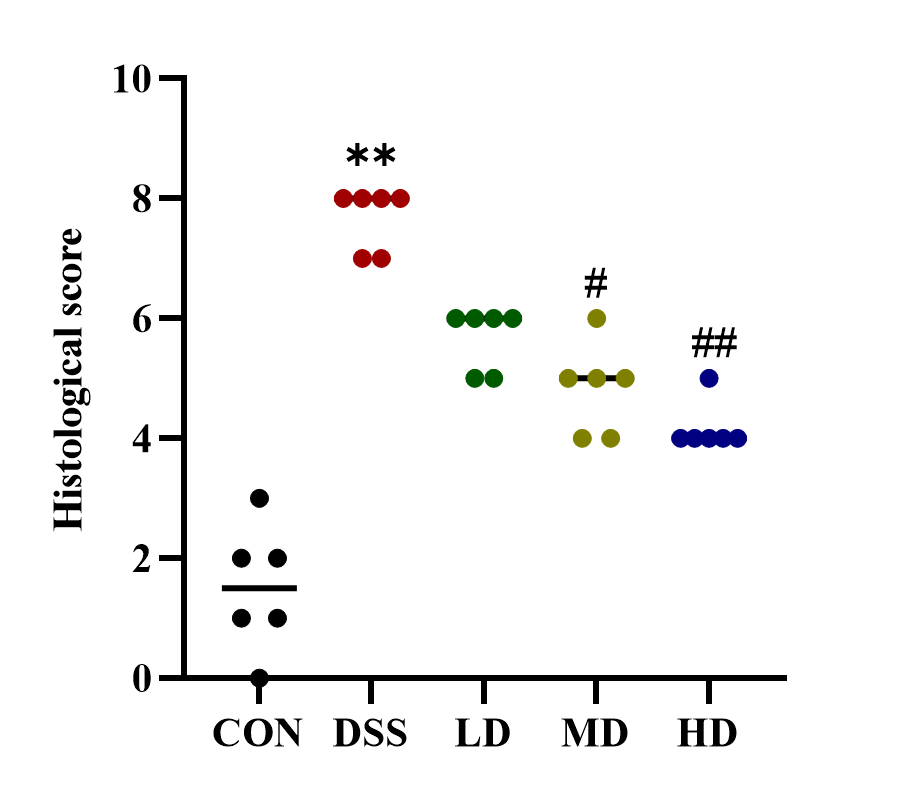

Supplement: Supplementary file 4 [file DataSheet6.ZIP › original data of figure 2-2/Figure8 HE score.tif]

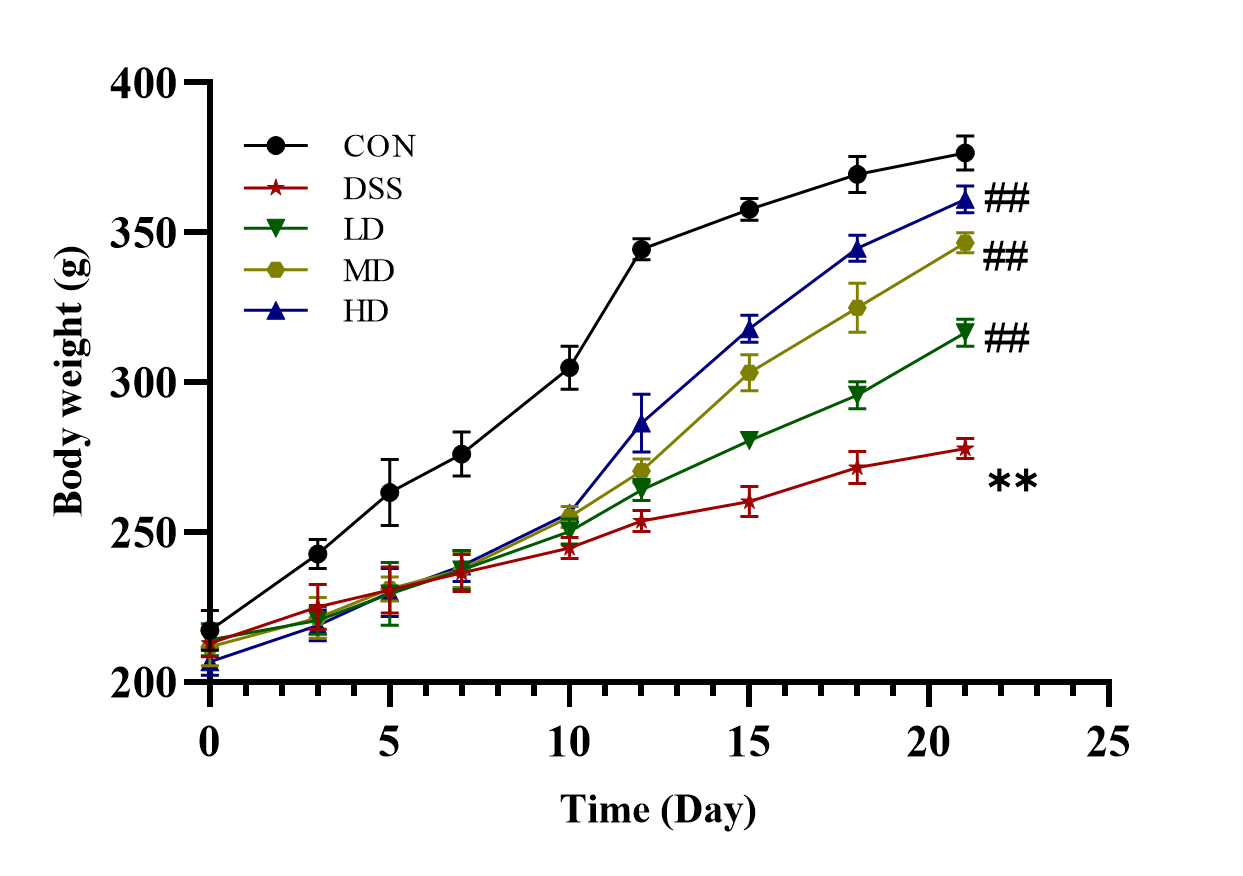

Supplement: Supplementary file 4 [file DataSheet6.ZIP › original data of figure 2-2/Figure9 weight.tif]

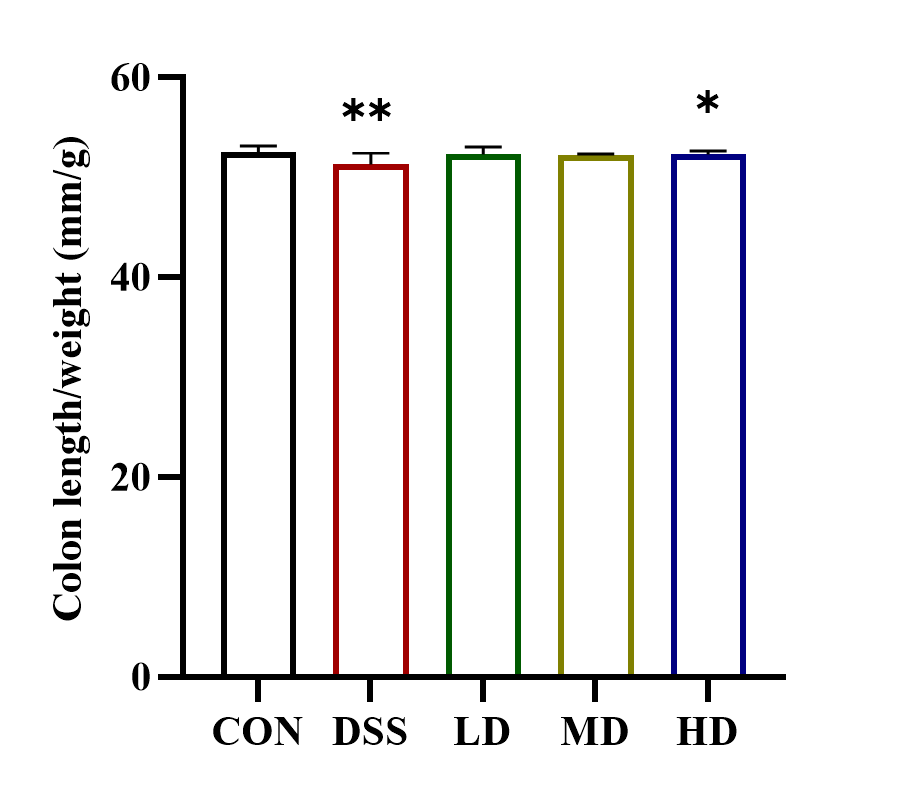

Supplement: Supplementary file 4 [file DataSheet6.ZIP › original data of figure 2-2/ratio.tif]

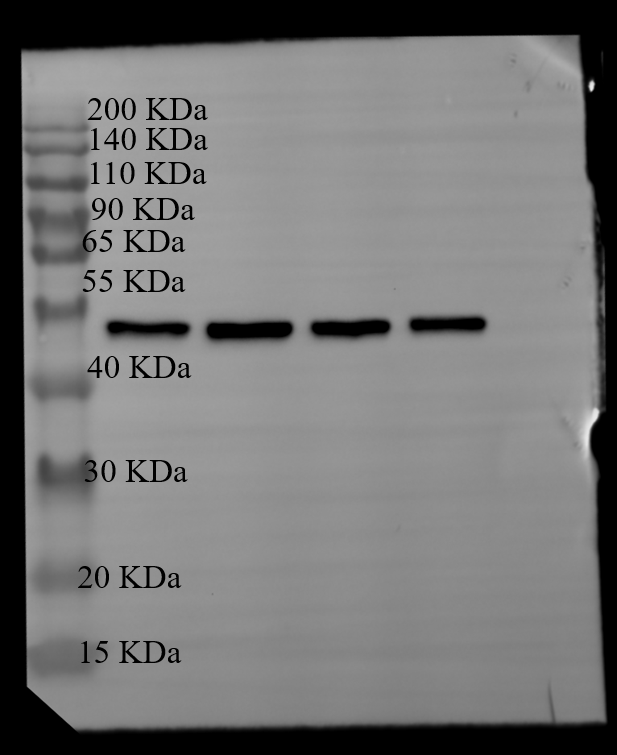

Supplement: Supplementary file 5 [file DataSheet2.ZIP › original data of figure 5/Caspase-1 in Caco2-1.tif]

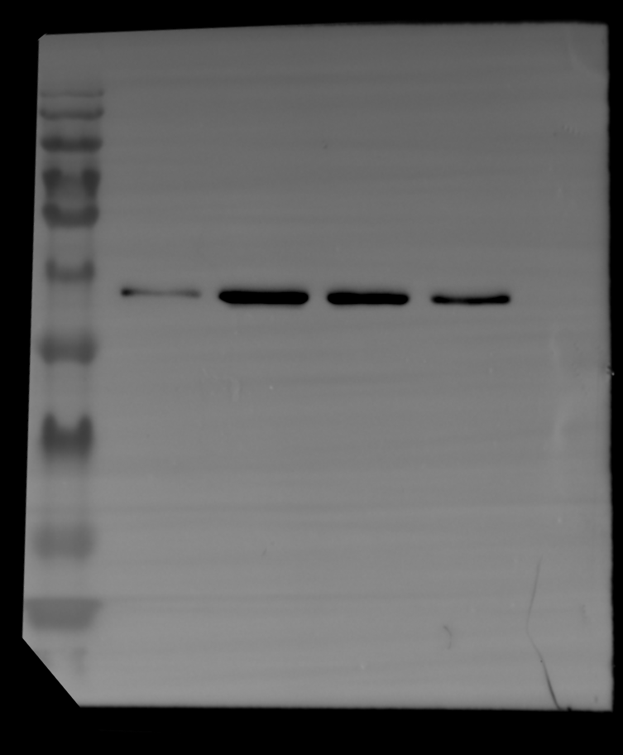

Supplement: Supplementary file 5 [file DataSheet2.ZIP › original data of figure 5/Caspase-1 in Caco2-2.tif]

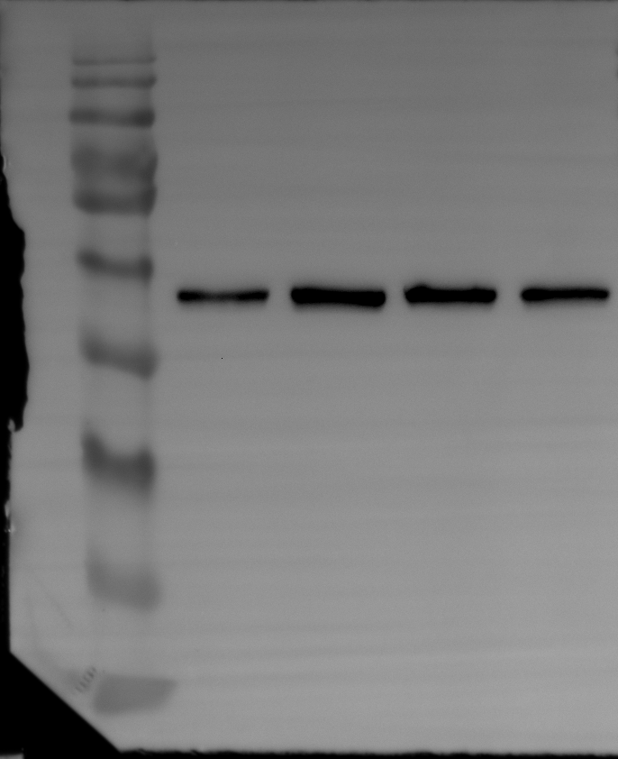

Supplement: Supplementary file 5 [file DataSheet2.ZIP › original data of figure 5/Caspase-1 in Caco2-3.tif]

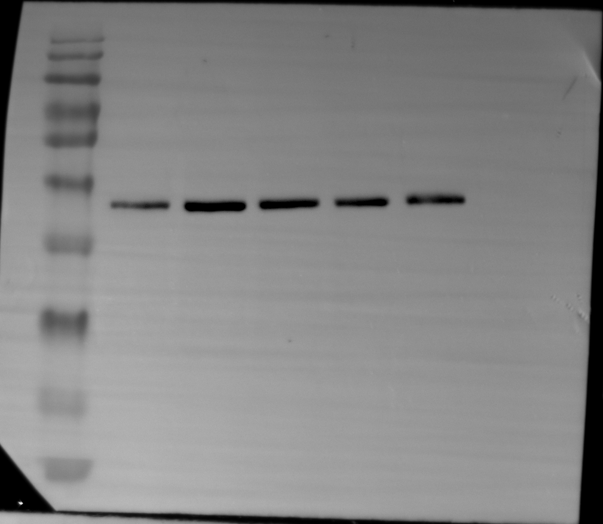

Supplement: Supplementary file 5 [file DataSheet2.ZIP › original data of figure 5/Caspase-1 in rats-1.tif]

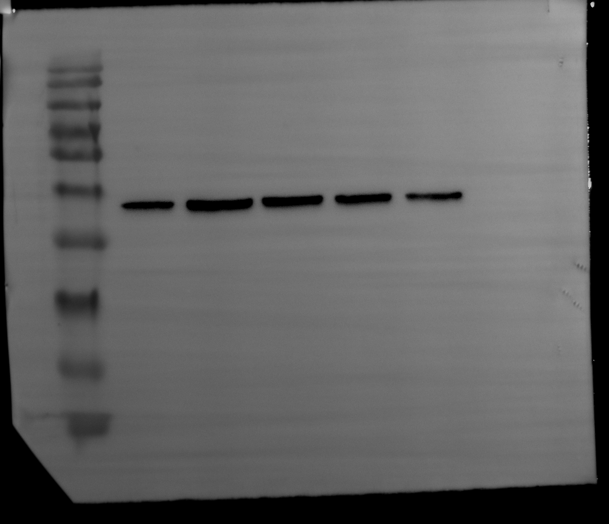

Supplement: Supplementary file 5 [file DataSheet2.ZIP › original data of figure 5/Caspase-1 in rats-2.tif]

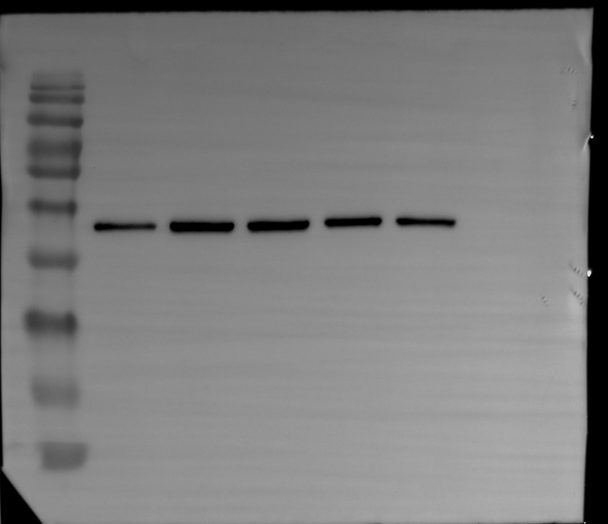

Supplement: Supplementary file 5 [file DataSheet2.ZIP › original data of figure 5/Caspase-1 in rats-3.tif]

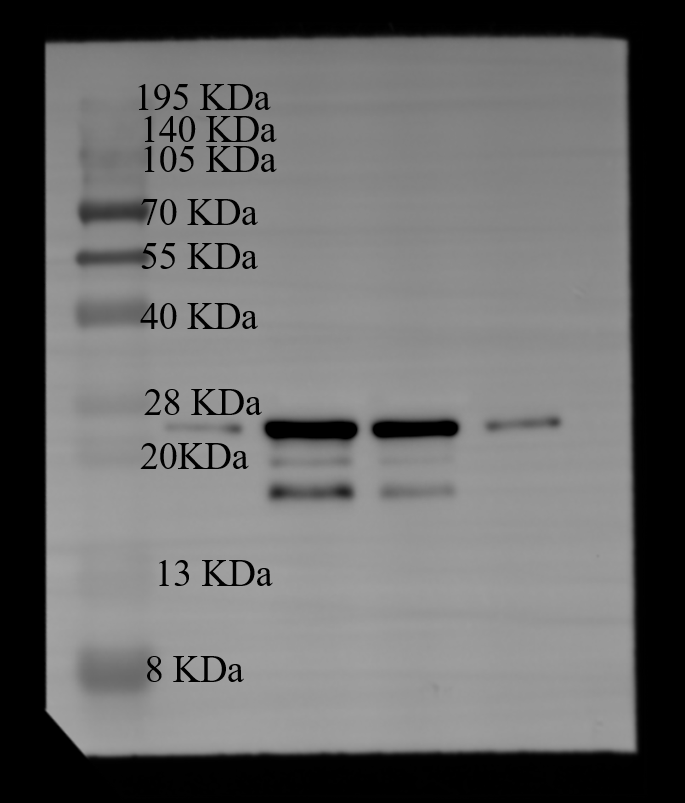

Supplement: Supplementary file 5 [file DataSheet2.ZIP › original data of figure 5/Cleaved-Caspase-1 in Caco2-1.tif]

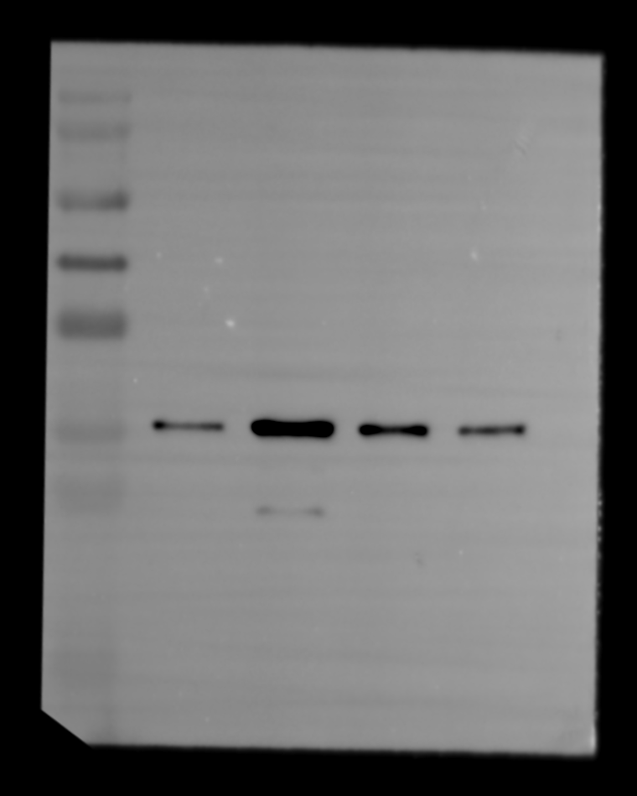

Supplement: Supplementary file 5 [file DataSheet2.ZIP › original data of figure 5/Cleaved-Caspase-1 in Caco2-2.tif]

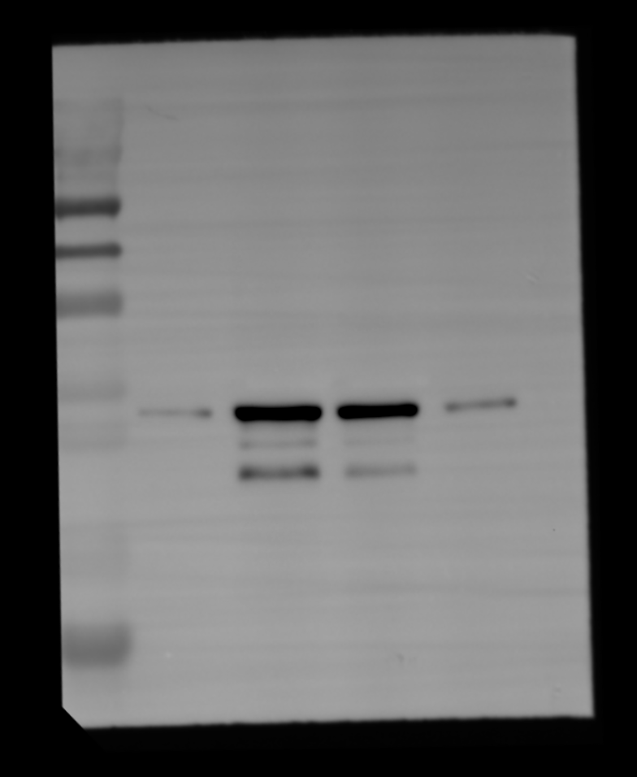

Supplement: Supplementary file 5 [file DataSheet2.ZIP › original data of figure 5/Cleaved-Caspase-1 in Caco2-3.tif]

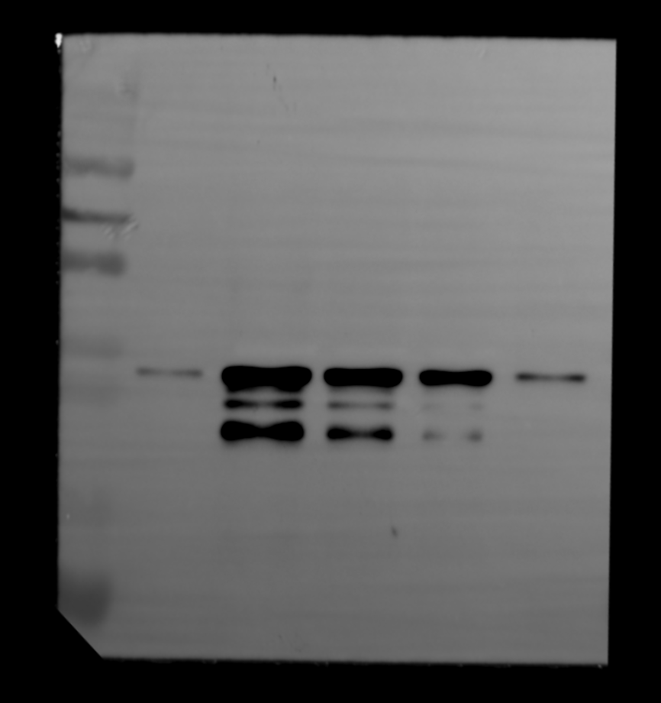

Supplement: Supplementary file 5 [file DataSheet2.ZIP › original data of figure 5/Cleaved-Caspase-1 in rats-1.tif]

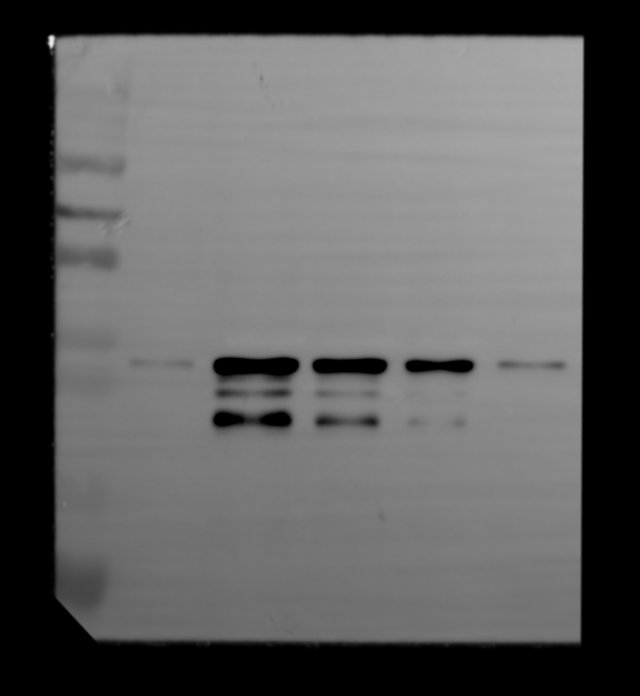

Supplement: Supplementary file 5 [file DataSheet2.ZIP › original data of figure 5/Cleaved-Caspase-1 in rats-2.tif]

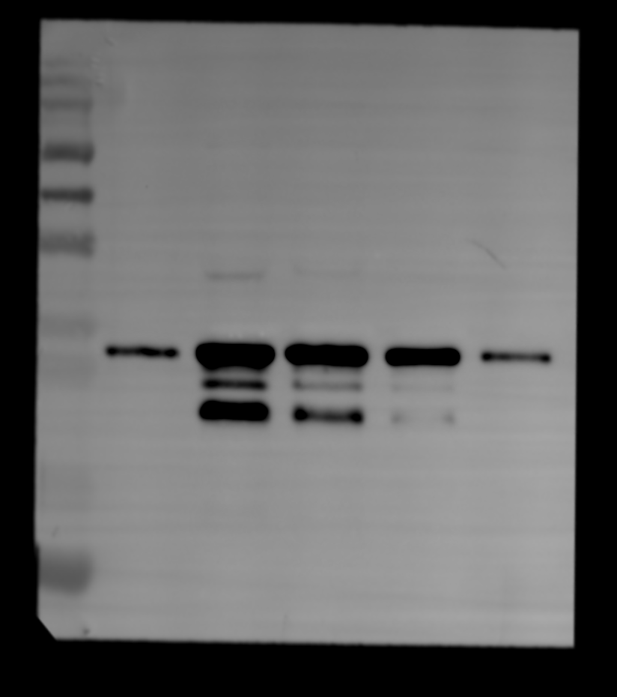

Supplement: Supplementary file 5 [file DataSheet2.ZIP › original data of figure 5/Cleaved-Caspase-1 in rats-3.tif]

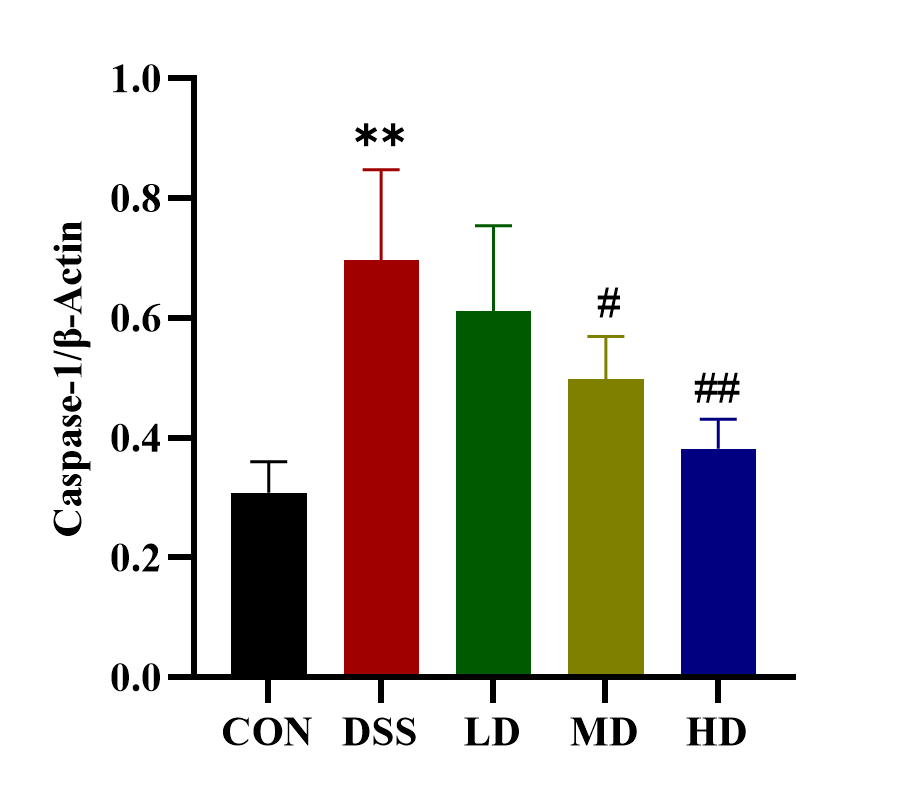

Supplement: Supplementary file 5 [file DataSheet2.ZIP › original data of figure 5/Figure1 rat Caspase-1.tif]

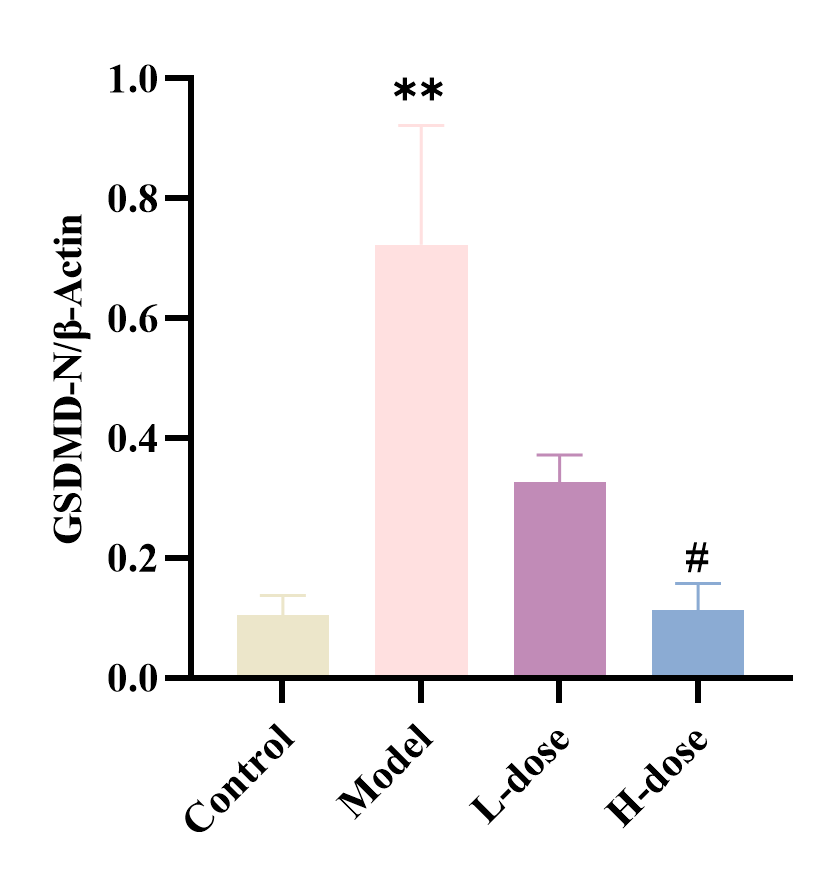

Supplement: Supplementary file 5 [file DataSheet2.ZIP › original data of figure 5/Figure10 cell GSDMD-N.tif]

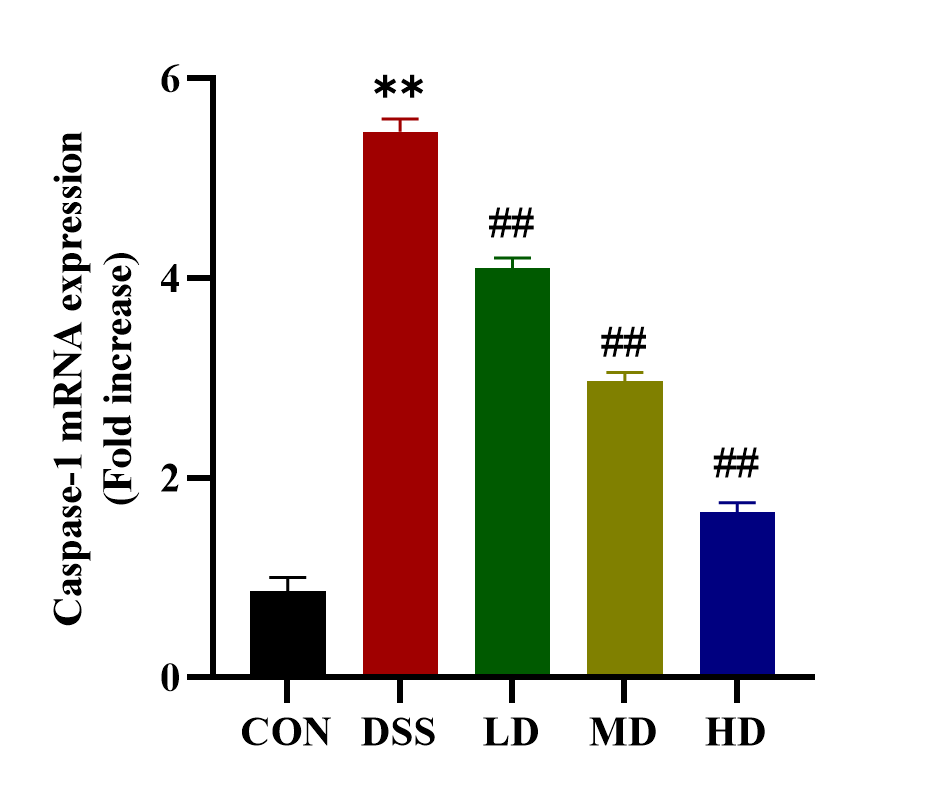

Supplement: Supplementary file 5 [file DataSheet2.ZIP › original data of figure 5/Figure11 pcr-rat Caspase-1.tif]

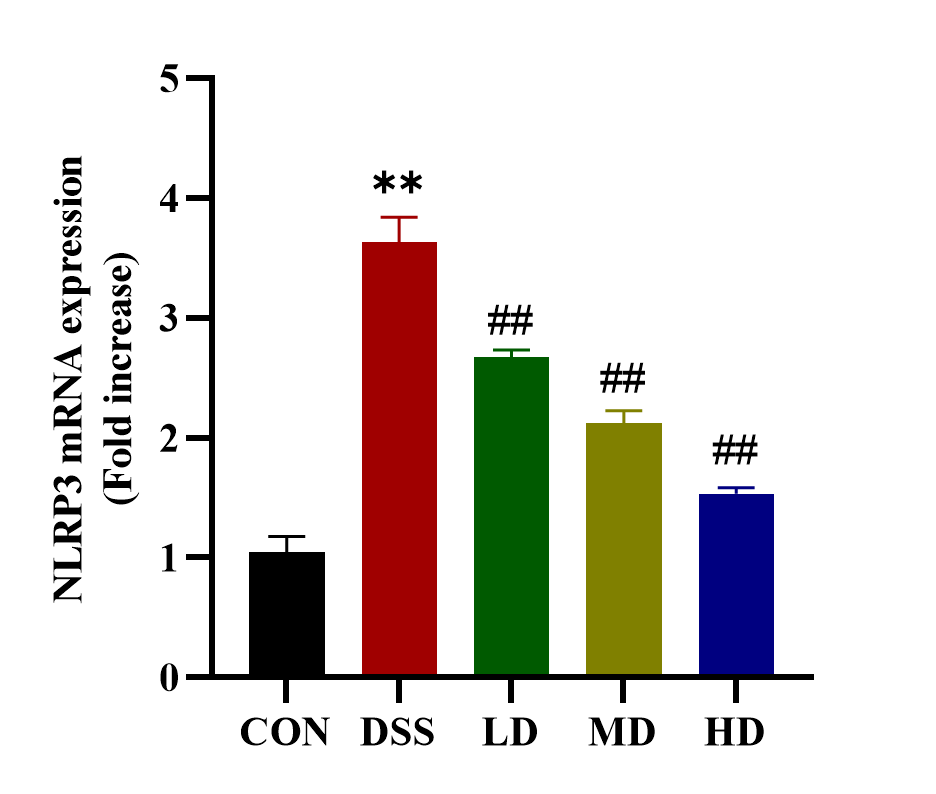

Supplement: Supplementary file 5 [file DataSheet2.ZIP › original data of figure 5/Figure12 pcr-rat NLRP3.tif]

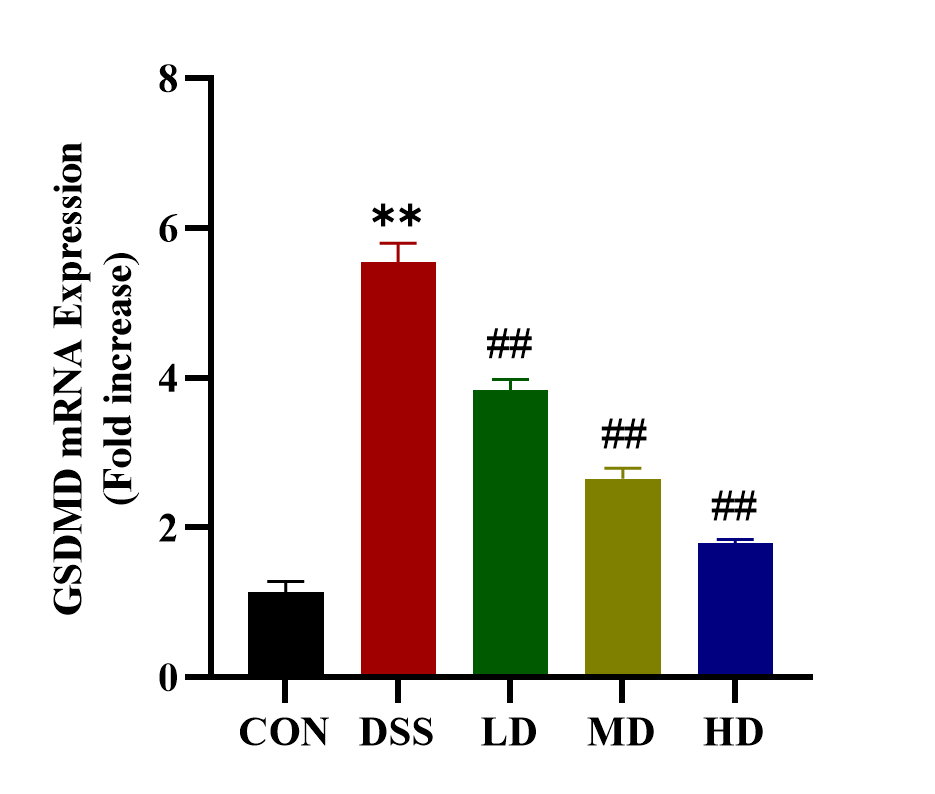

Supplement: Supplementary file 5 [file DataSheet2.ZIP › original data of figure 5/Figure13 pcr-rat GSDMD.tif]

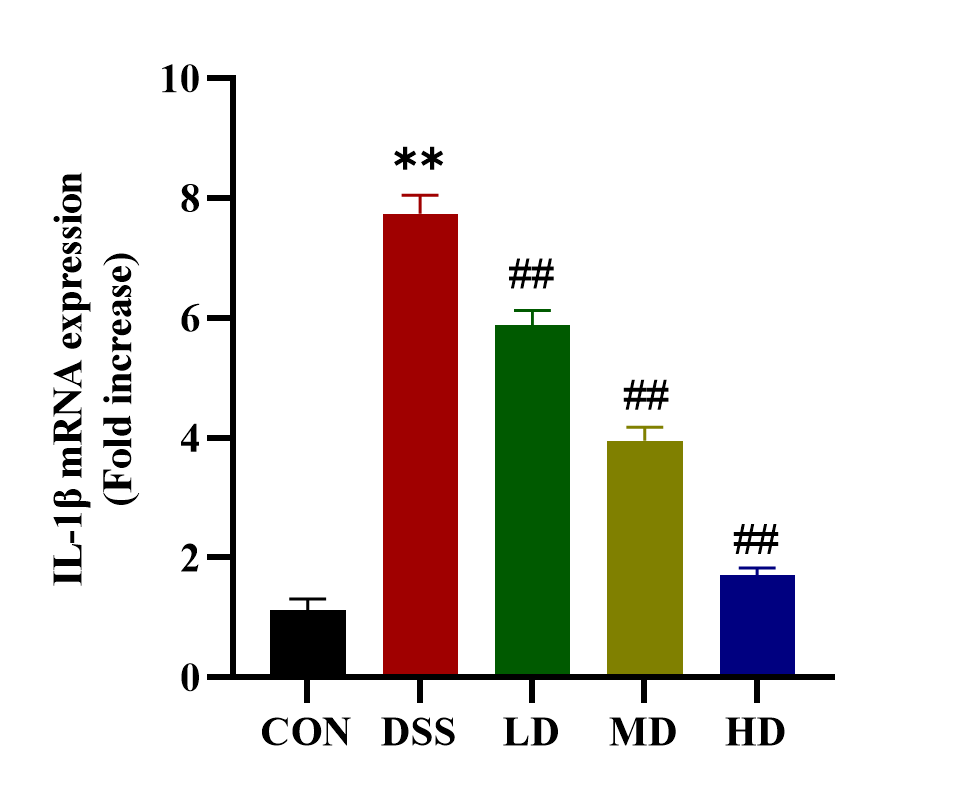

Supplement: Supplementary file 5 [file DataSheet2.ZIP › original data of figure 5/Figure14 pcr-rat IL-1beta.tif]

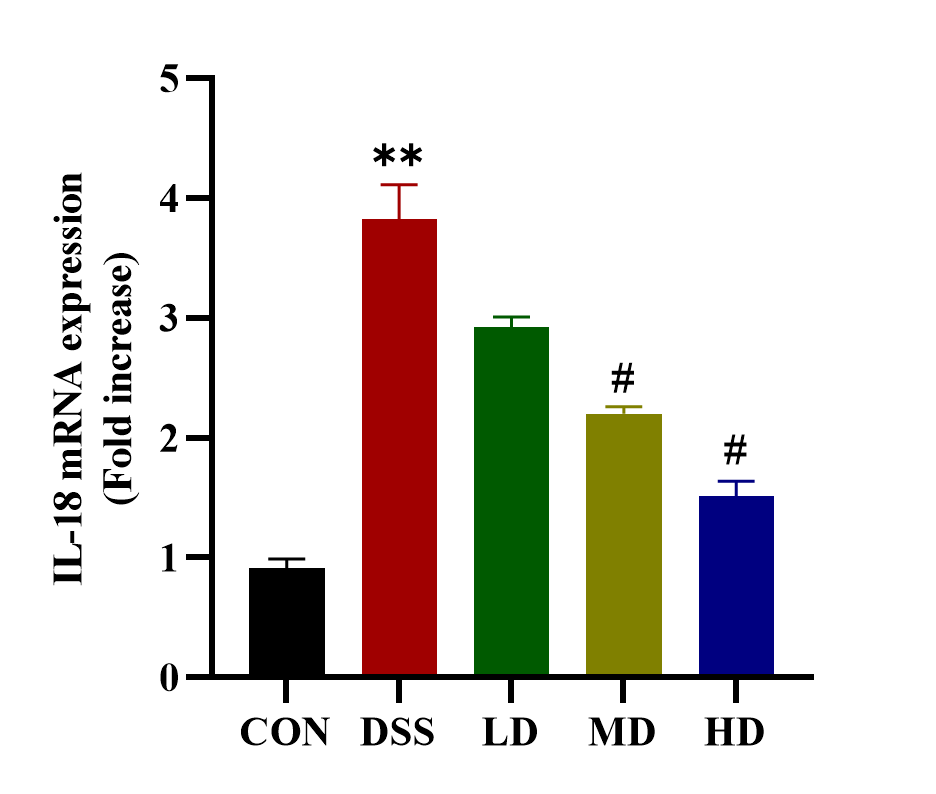

Supplement: Supplementary file 5 [file DataSheet2.ZIP › original data of figure 5/Figure15 pcr-rat IL-18.tif]

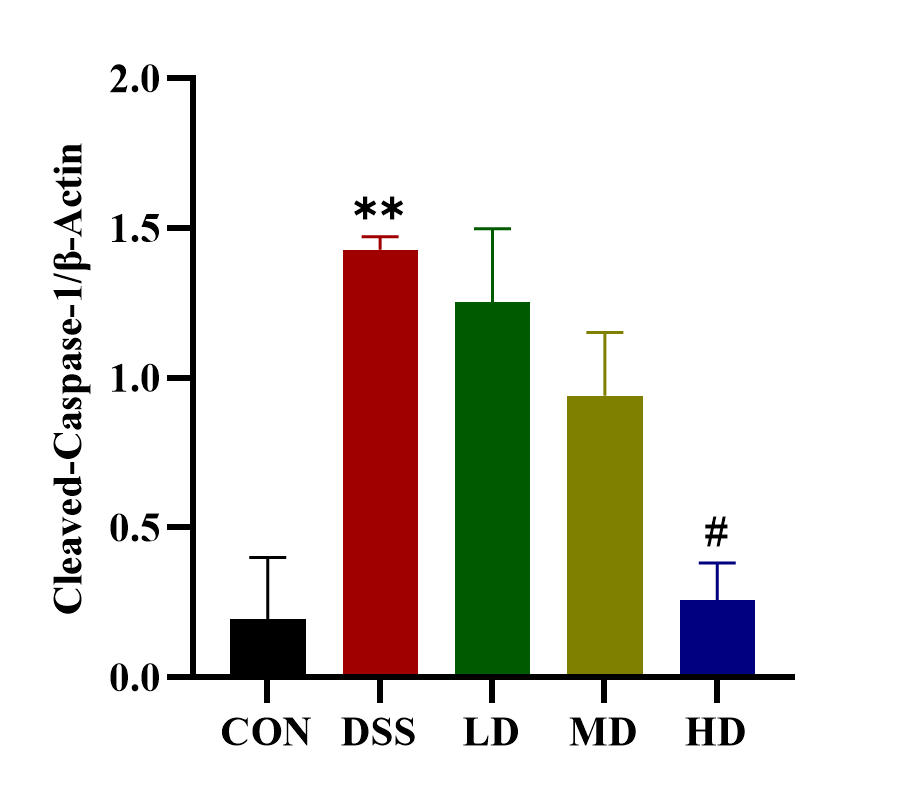

Supplement: Supplementary file 5 [file DataSheet2.ZIP › original data of figure 5/Figure2 rat Cleaved-Caspase-1.tif]

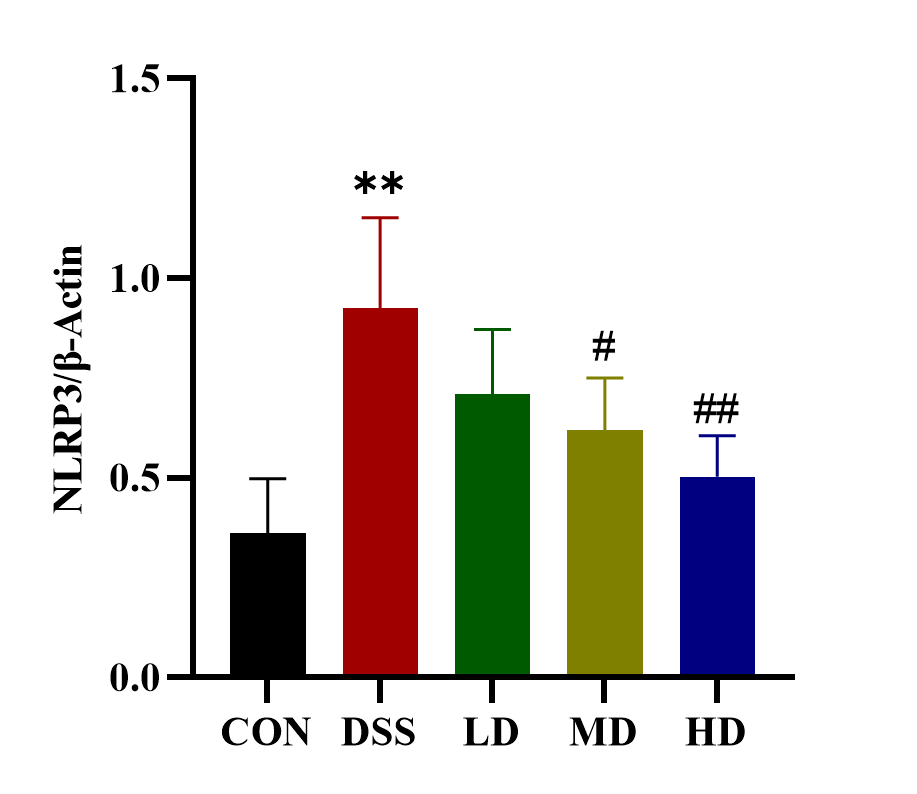

Supplement: Supplementary file 5 [file DataSheet2.ZIP › original data of figure 5/Figure3 rat NLRP3.tif]

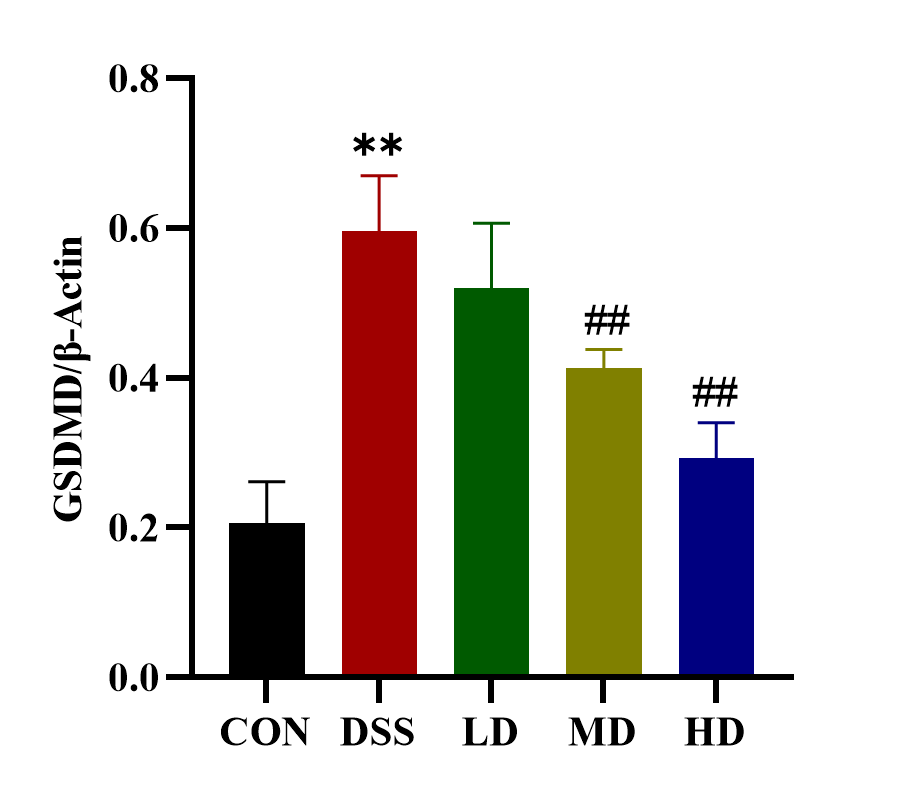

Supplement: Supplementary file 5 [file DataSheet2.ZIP › original data of figure 5/Figure4 rat GSDMD.tif]

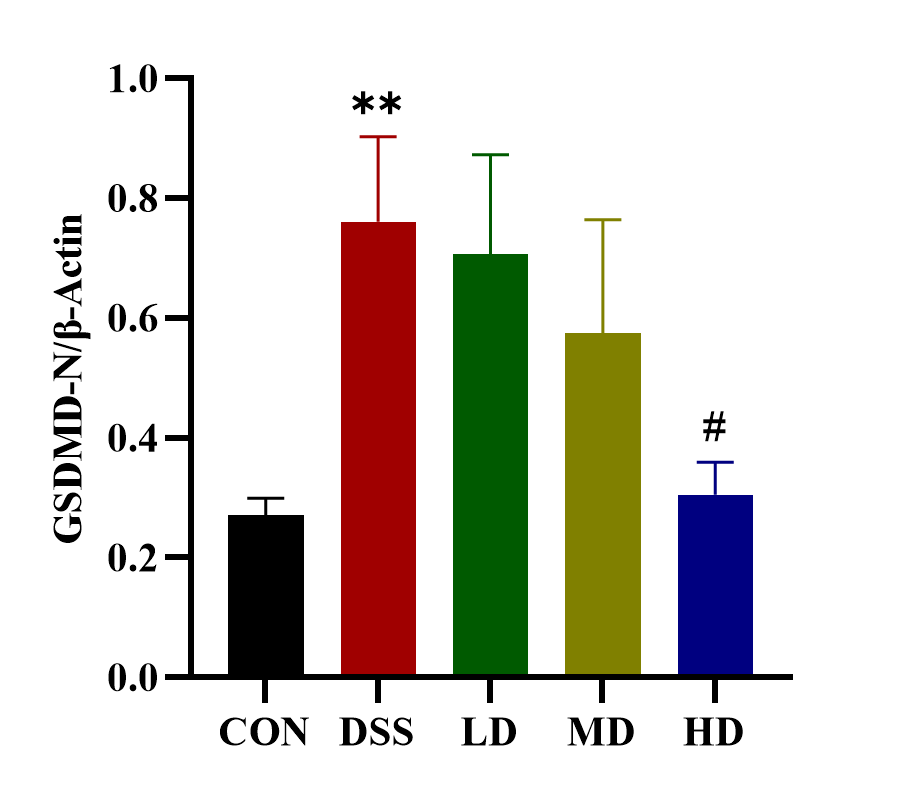

Supplement: Supplementary file 5 [file DataSheet2.ZIP › original data of figure 5/Figure5 rat GSDMD-N.tif]

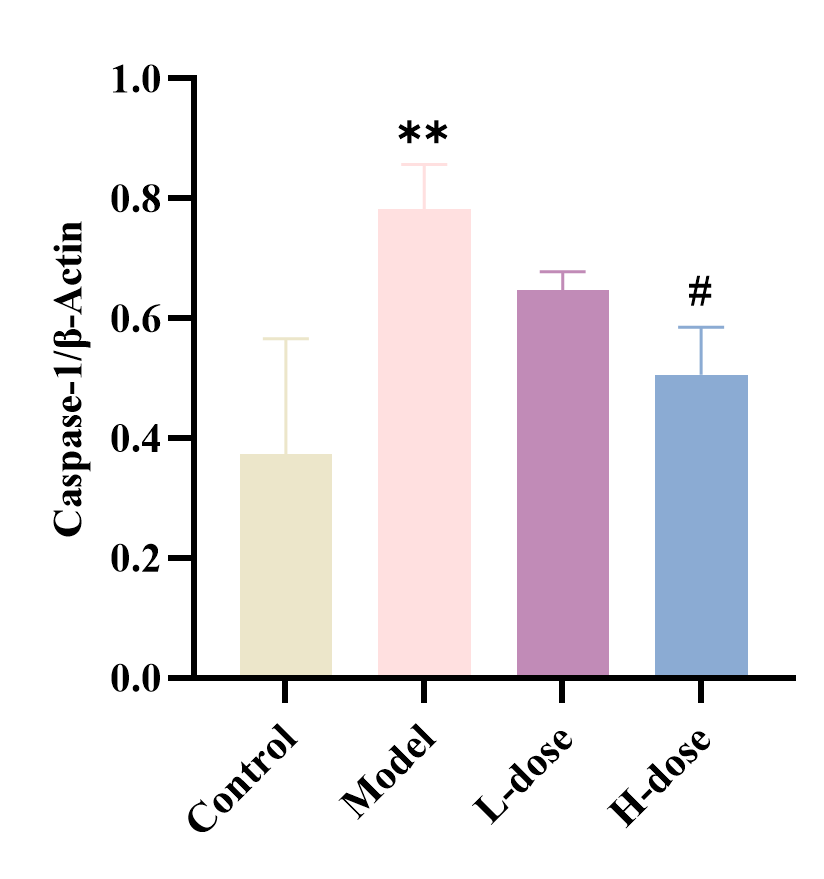

Supplement: Supplementary file 5 [file DataSheet2.ZIP › original data of figure 5/Figure6 cell Caspase-1.tif]

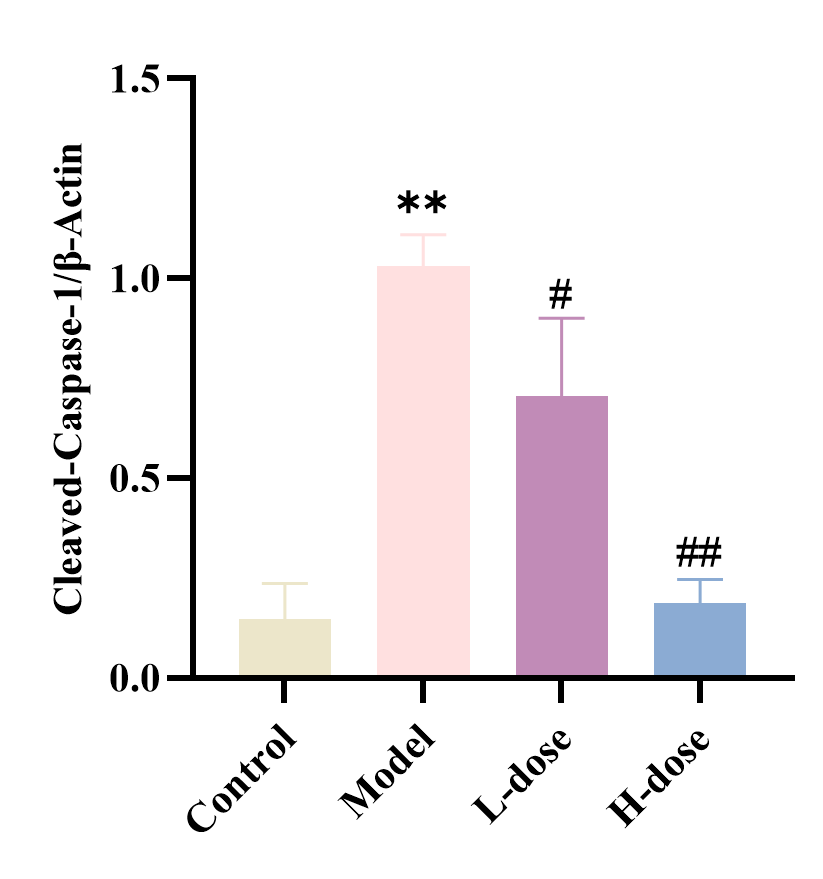

Supplement: Supplementary file 5 [file DataSheet2.ZIP › original data of figure 5/Figure7 cell Cleaved-Caspase-1.tif]

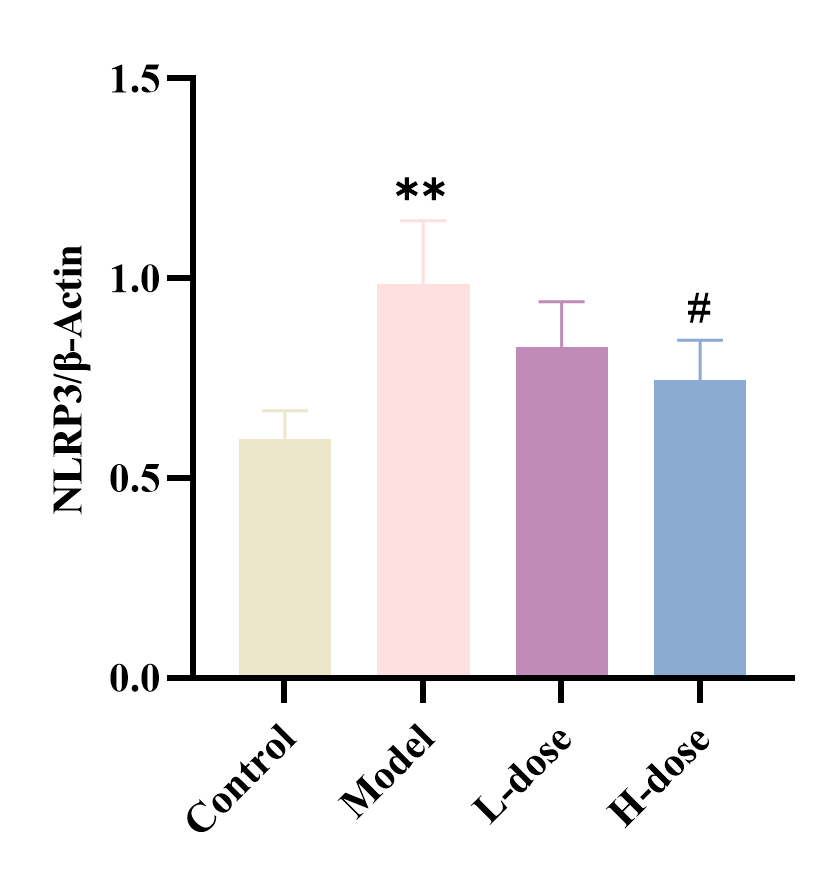

Supplement: Supplementary file 5 [file DataSheet2.ZIP › original data of figure 5/Figure8 cell NLRP3.tif]

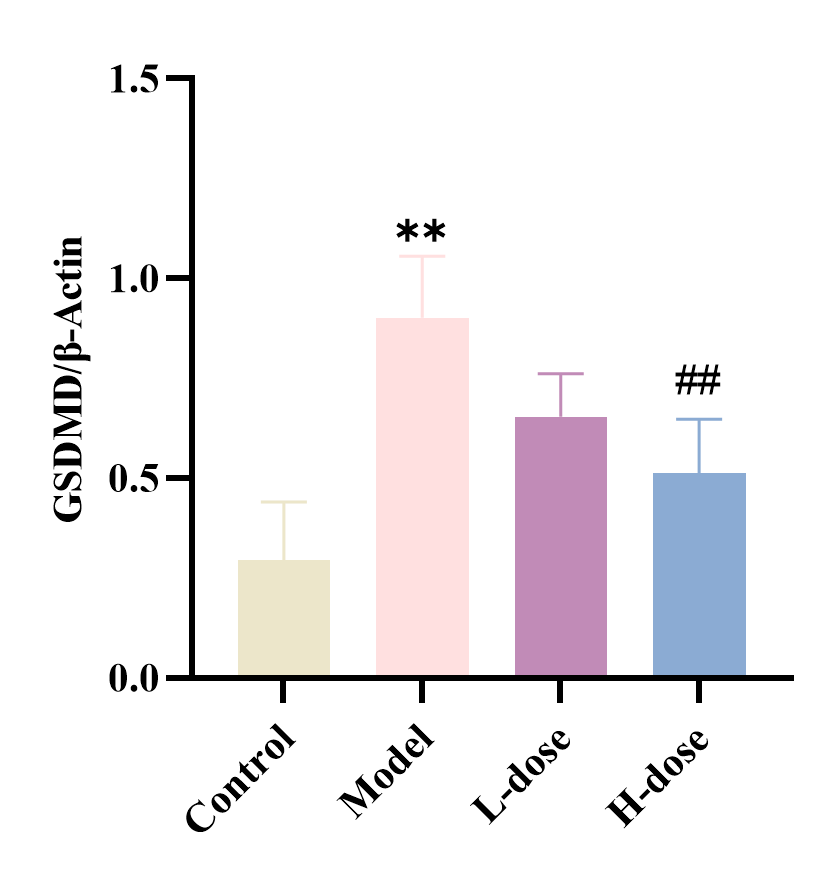

Supplement: Supplementary file 5 [file DataSheet2.ZIP › original data of figure 5/Figure9 cell GSDMD.tif]

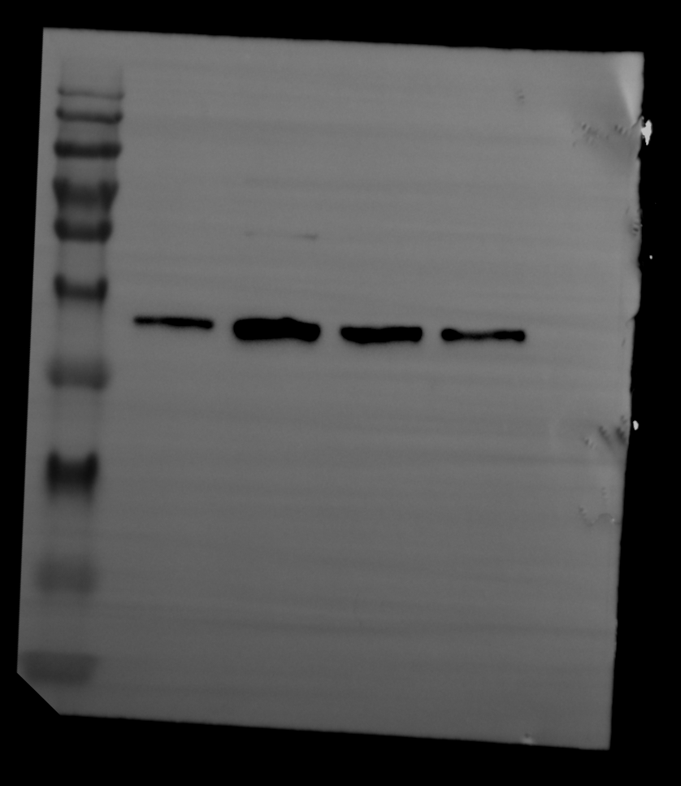

Supplement: Supplementary file 5 [file DataSheet2.ZIP › original data of figure 5/GSDMD in Caco2-1.tif]

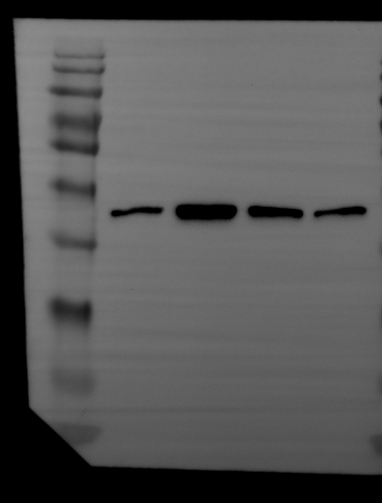

Supplement: Supplementary file 5 [file DataSheet2.ZIP › original data of figure 5/GSDMD in Caco2-2.tif]

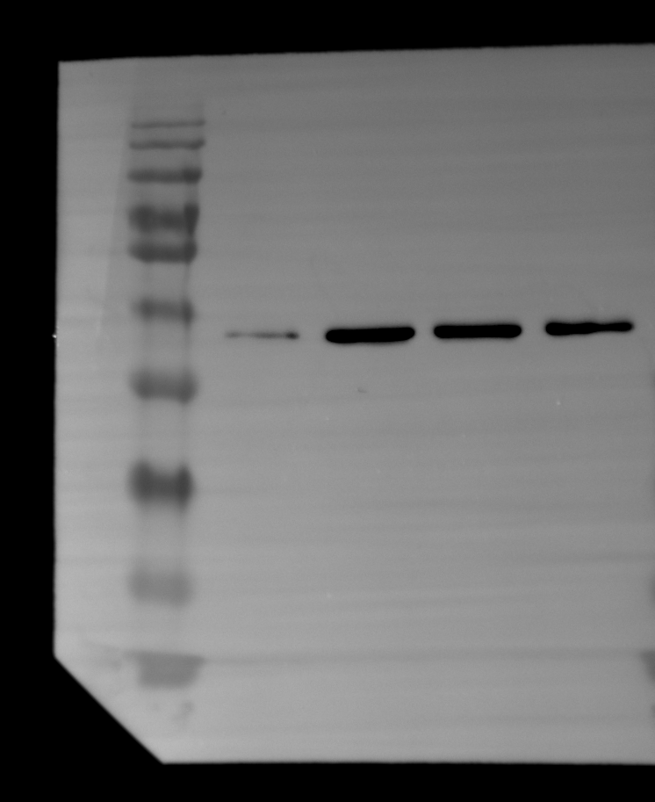

Supplement: Supplementary file 5 [file DataSheet2.ZIP › original data of figure 5/GSDMD in Caco2-3.tif]

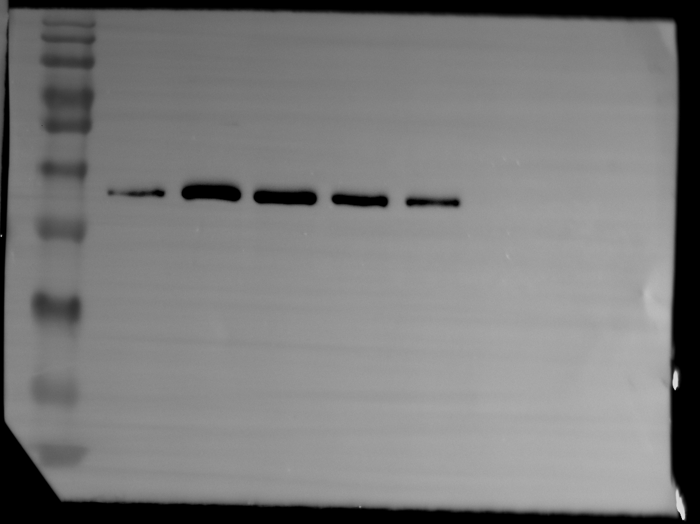

Supplement: Supplementary file 5 [file DataSheet2.ZIP › original data of figure 5/GSDMD in rats-1.tif]

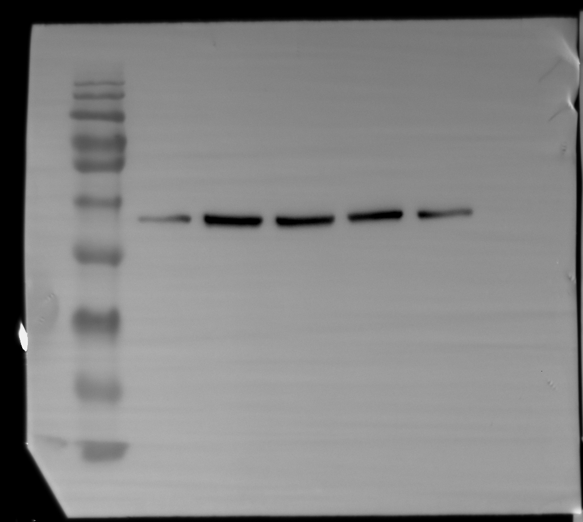

Supplement: Supplementary file 5 [file DataSheet2.ZIP › original data of figure 5/GSDMD in rats-2.tif]

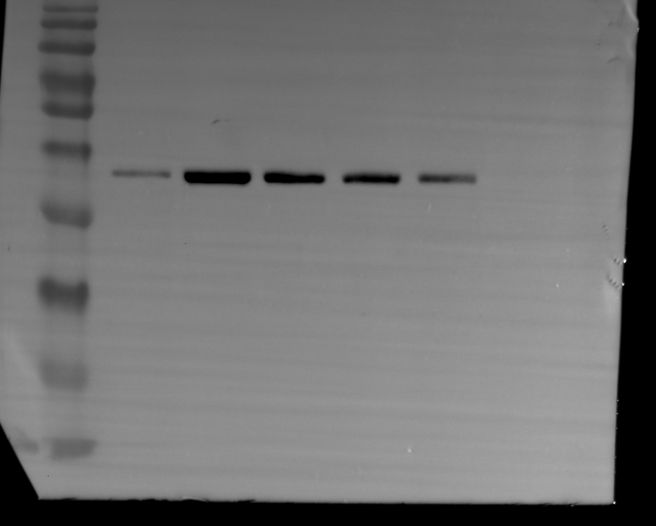

Supplement: Supplementary file 5 [file DataSheet2.ZIP › original data of figure 5/GSDMD in rats-3.tif]

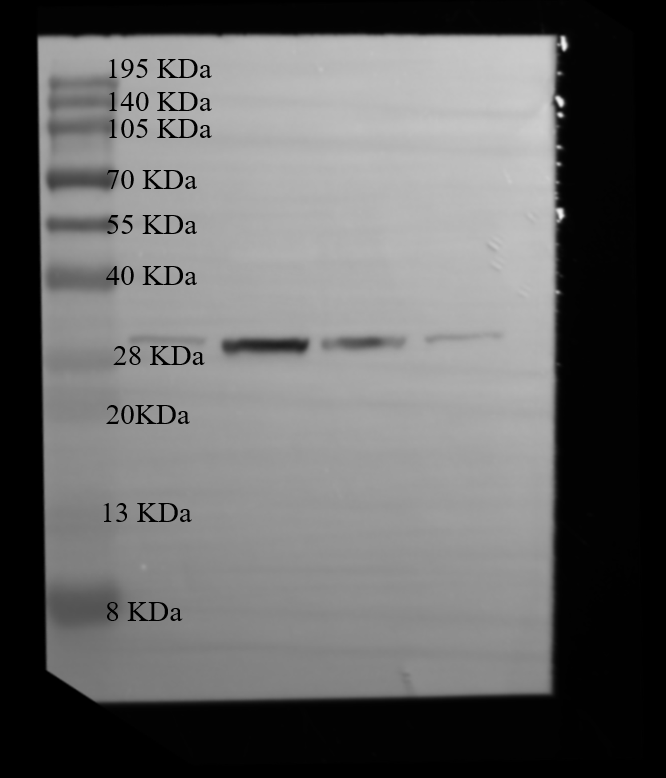

Supplement: Supplementary file 5 [file DataSheet2.ZIP › original data of figure 5/GSDMD-N in Caco2-1.tif]

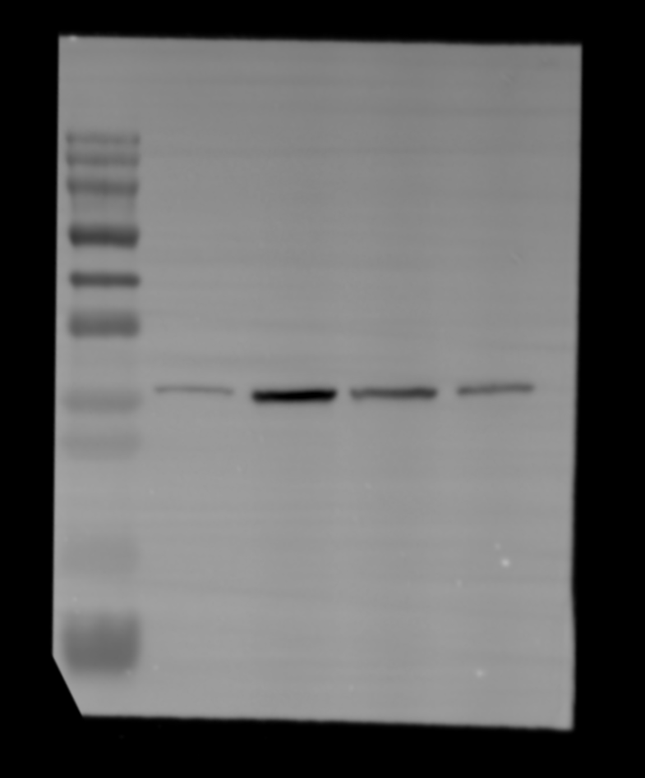

Supplement: Supplementary file 5 [file DataSheet2.ZIP › original data of figure 5/GSDMD-N in Caco2-2.tif]

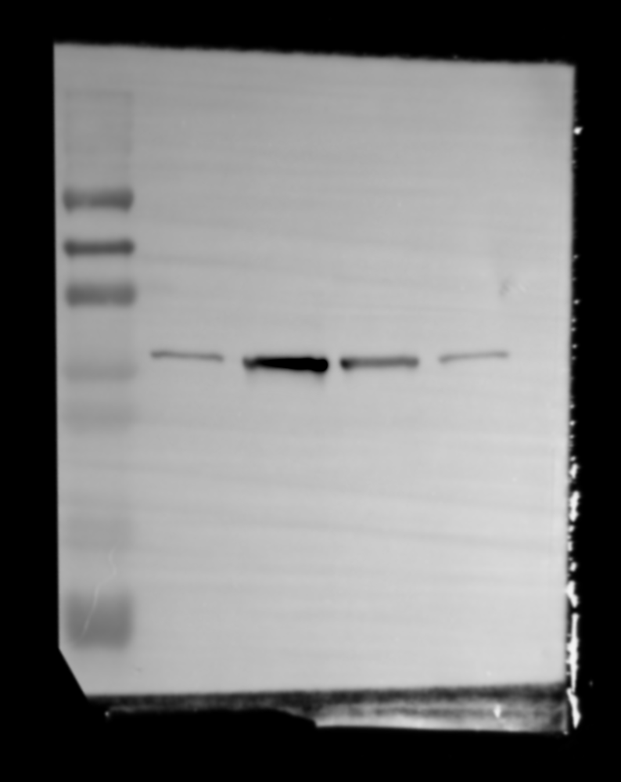

Supplement: Supplementary file 5 [file DataSheet2.ZIP › original data of figure 5/GSDMD-N in Caco2-3.tif]

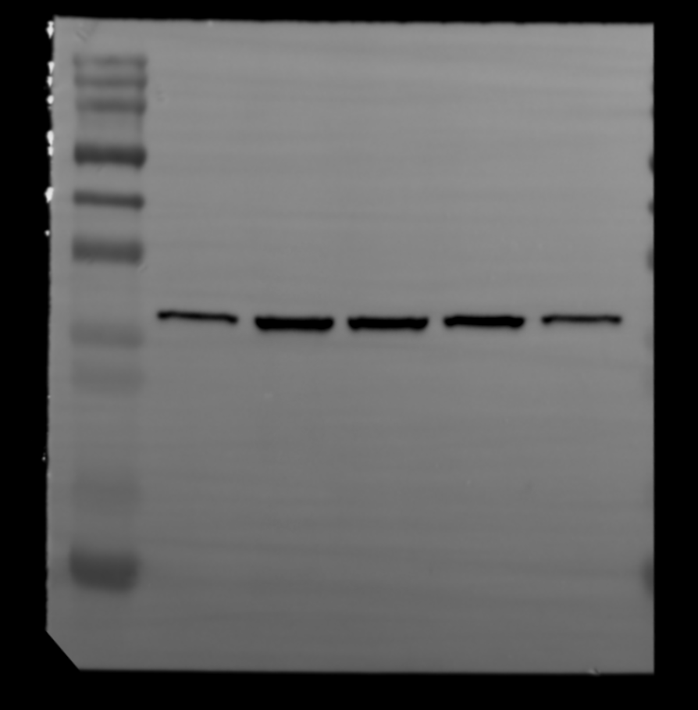

Supplement: Supplementary file 5 [file DataSheet2.ZIP › original data of figure 5/GSDMD-N in rats-1.tif]

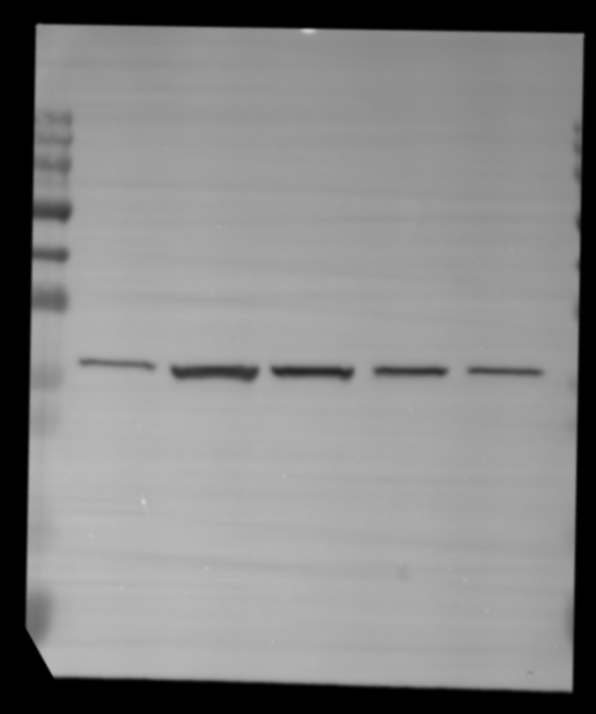

Supplement: Supplementary file 5 [file DataSheet2.ZIP › original data of figure 5/GSDMD-N in rats-2.tif]

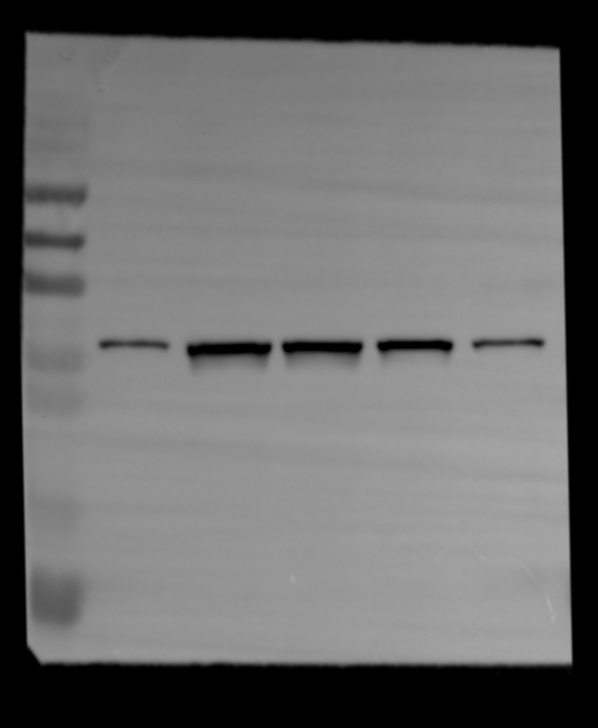

Supplement: Supplementary file 5 [file DataSheet2.ZIP › original data of figure 5/GSDMD-N in rats-3.tif]

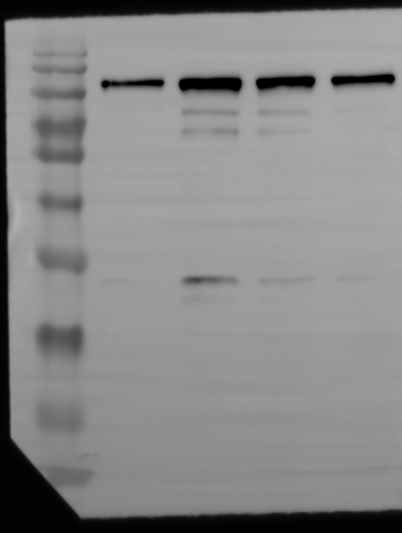

Supplement: Supplementary file 5 [file DataSheet2.ZIP › original data of figure 5/NLRP3 in Caco2-1.tif]

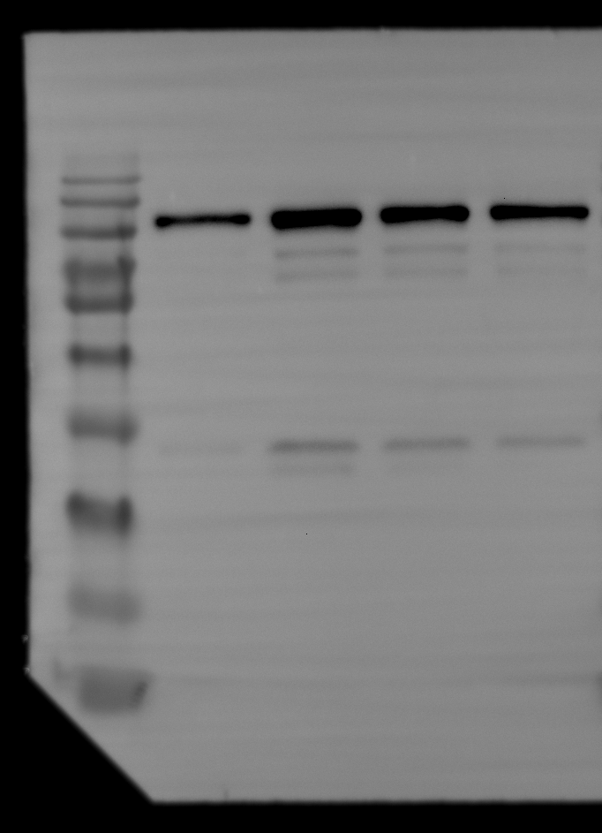

Supplement: Supplementary file 5 [file DataSheet2.ZIP › original data of figure 5/NLRP3 in Caco2-2.tif]

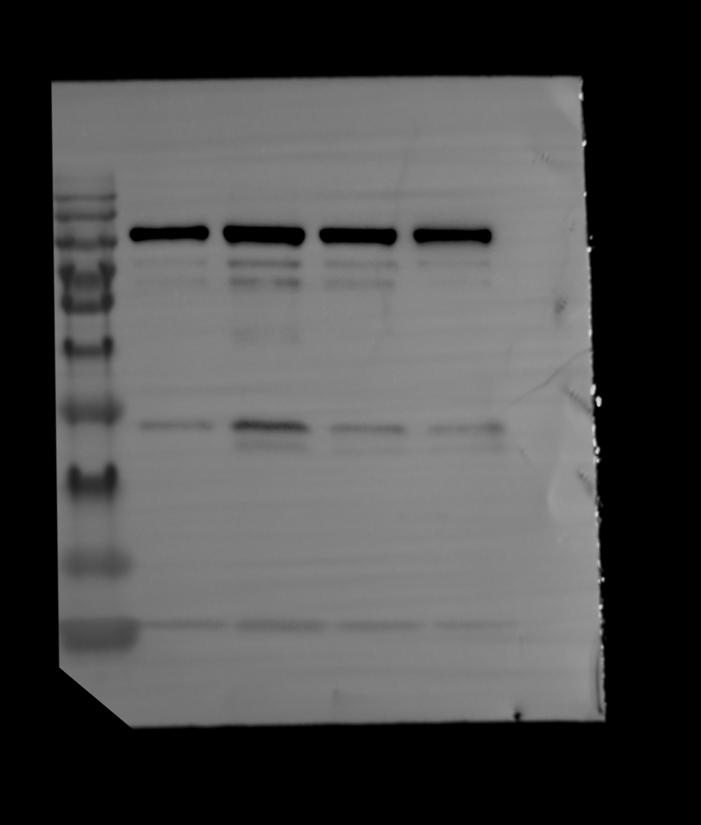

Supplement: Supplementary file 5 [file DataSheet2.ZIP › original data of figure 5/NLRP3 in Caco2-3.tif]

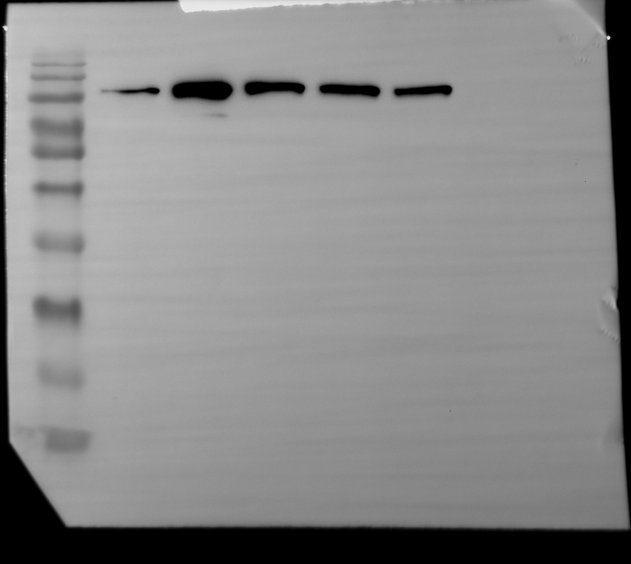

Supplement: Supplementary file 5 [file DataSheet2.ZIP › original data of figure 5/NLRP3 in rats-1.tif]

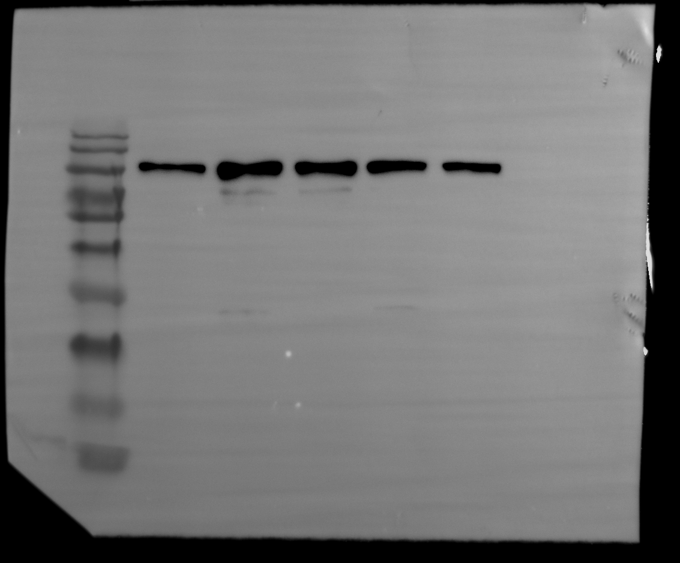

Supplement: Supplementary file 5 [file DataSheet2.ZIP › original data of figure 5/NLRP3 in rats-2.tif]

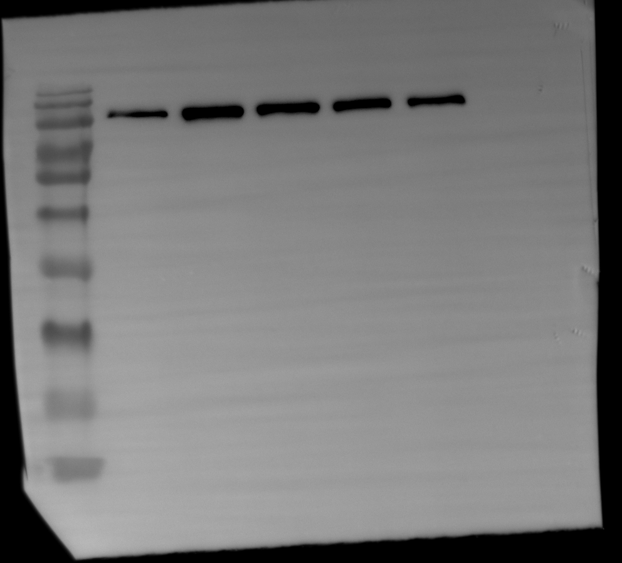

Supplement: Supplementary file 5 [file DataSheet2.ZIP › original data of figure 5/NLRP3 in rats-3.tif]

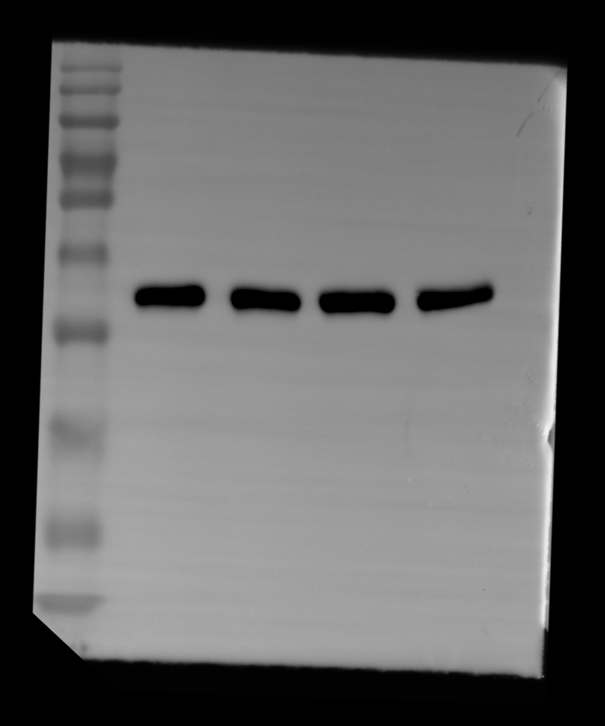

Supplement: Supplementary file 5 [file DataSheet2.ZIP › original data of figure 5/β-Actin in Caco2-1.tif]

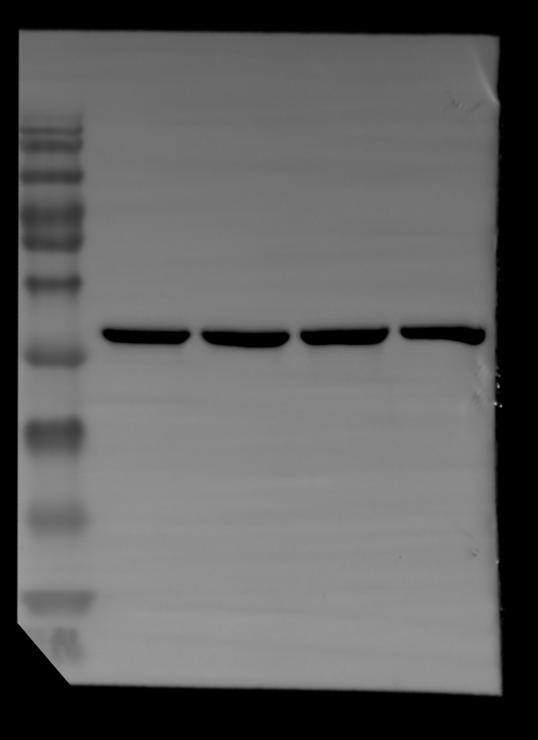

Supplement: Supplementary file 5 [file DataSheet2.ZIP › original data of figure 5/β-Actin in Caco2-2.tif]

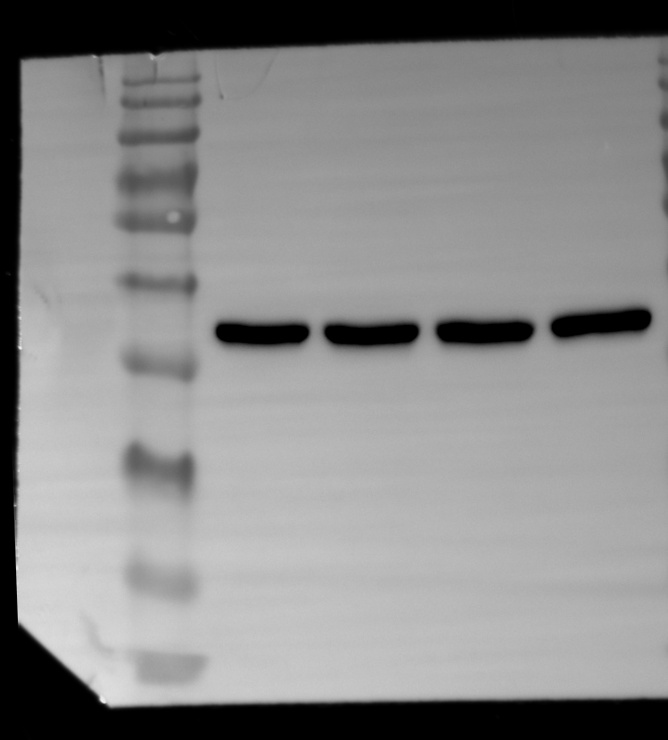

Supplement: Supplementary file 5 [file DataSheet2.ZIP › original data of figure 5/β-Actin in Caco2-3.tif]
