# Supplementary material for: β-sitosterol alleviates dextran sulfate sodium-induced experimental colitis via inhibition of NLRP3/Caspase-1/GSDMD-mediated pyroptosis
Source: Front Pharmacol. 2023 Oct 26;14:1218477. doi: 10.3389/fphar.2023.1218477 (PMC10637366; doi:10.3389/fphar.2023.1218477)

Caspase-1 in rats

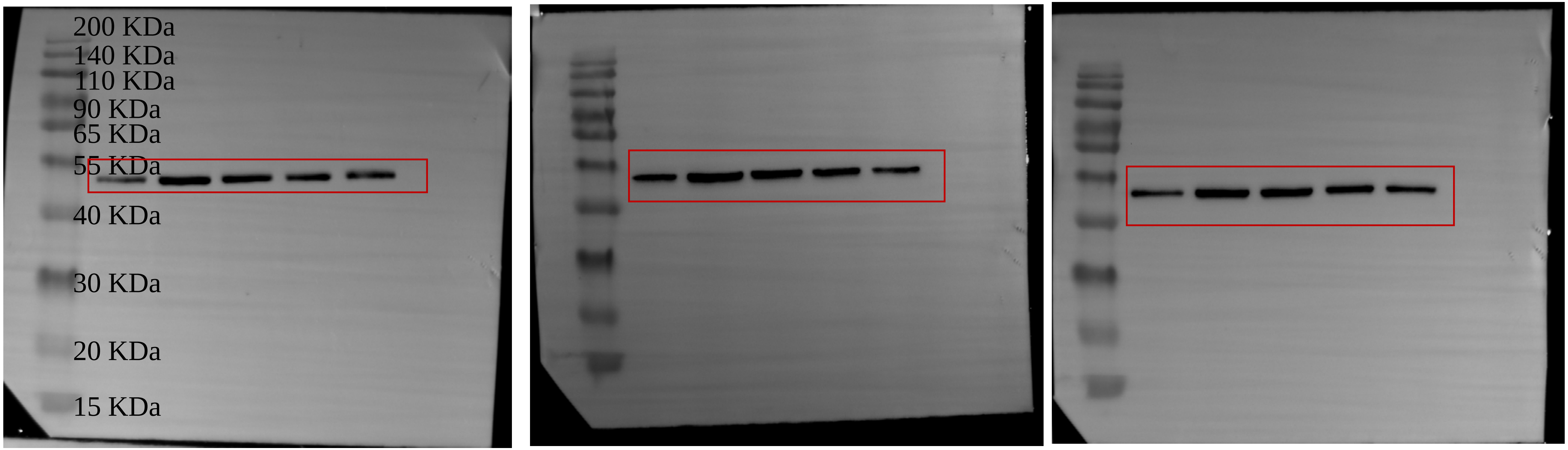

Cleaved-Caspase-1 in rats

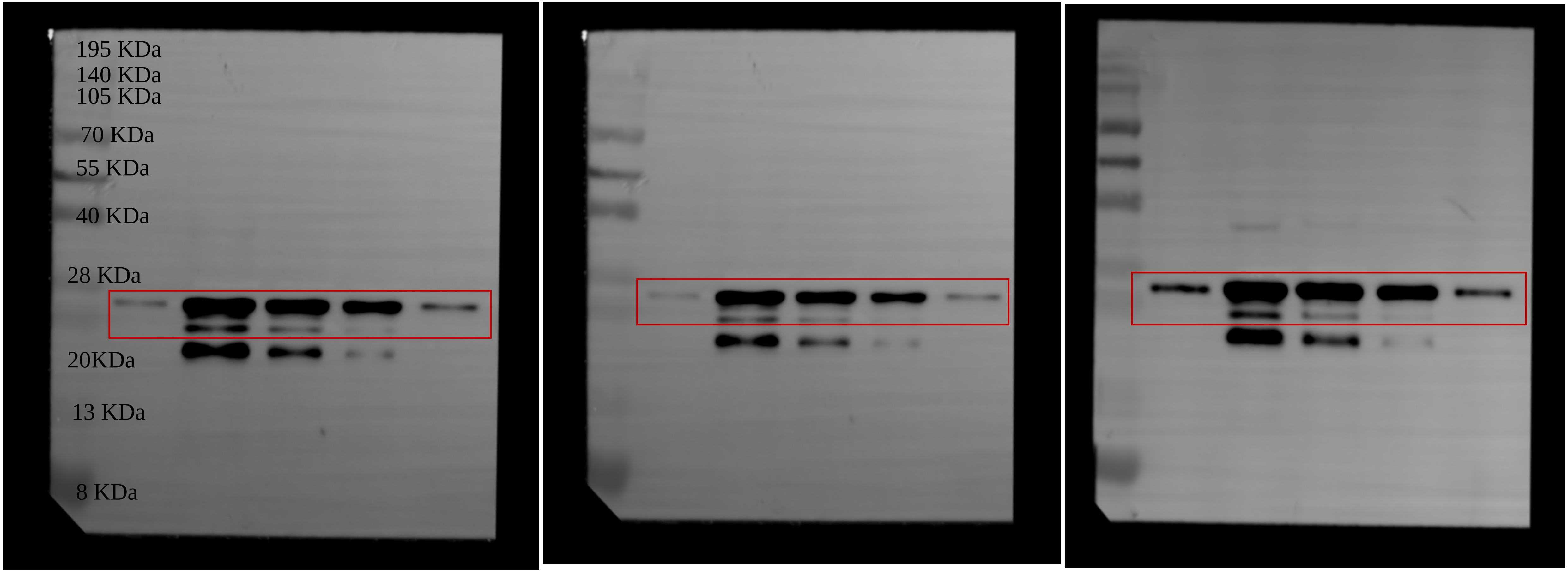

NLRP3 in rats

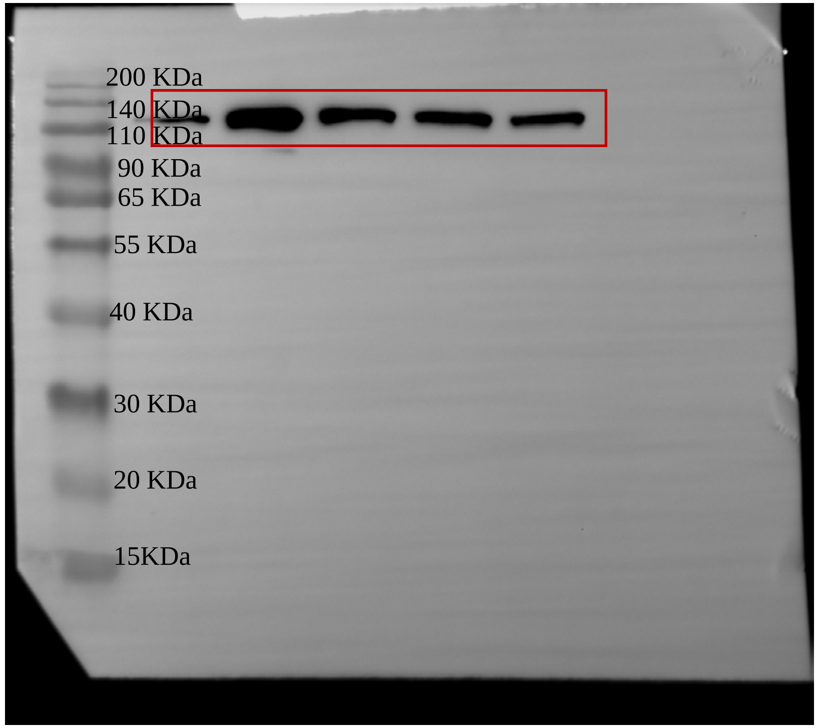

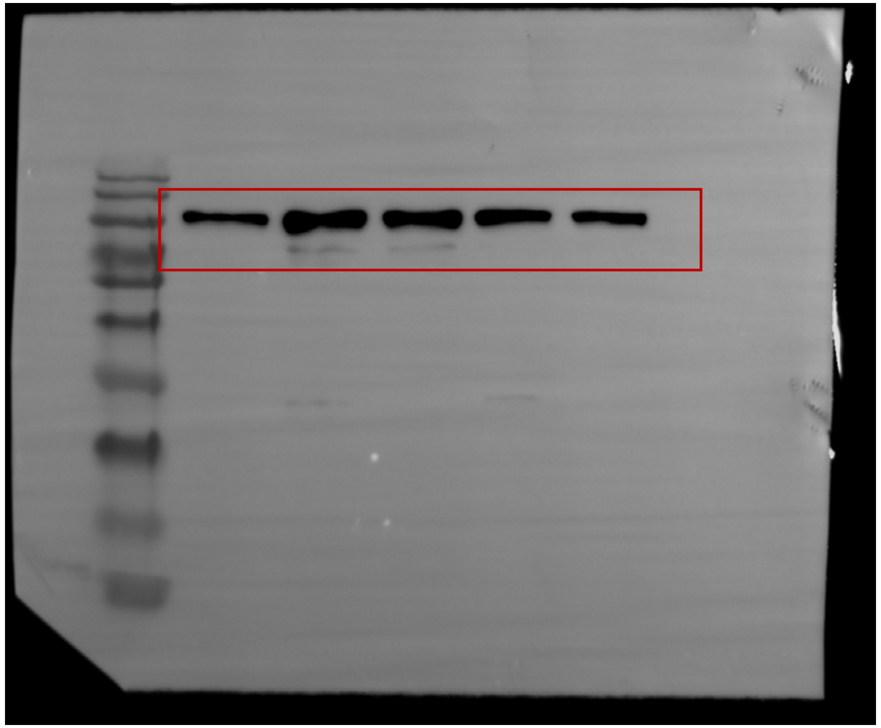

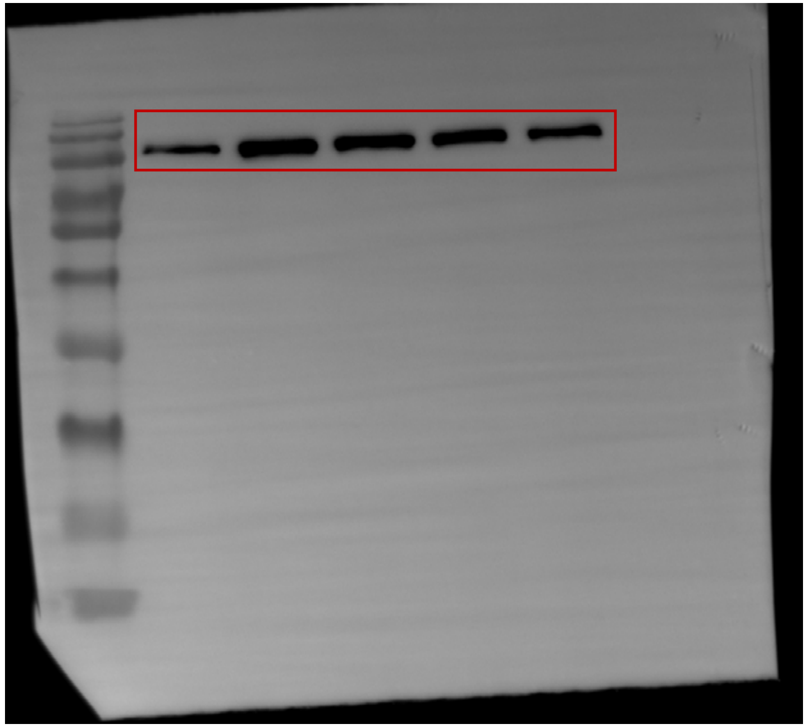

GSDMD in rats

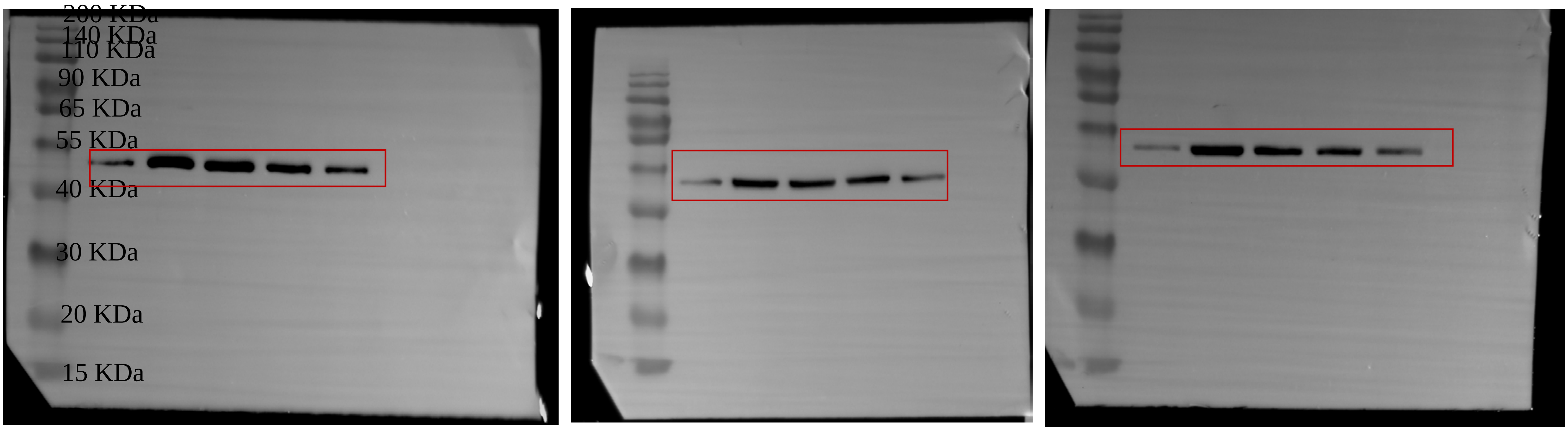

GSDMD-N in rats

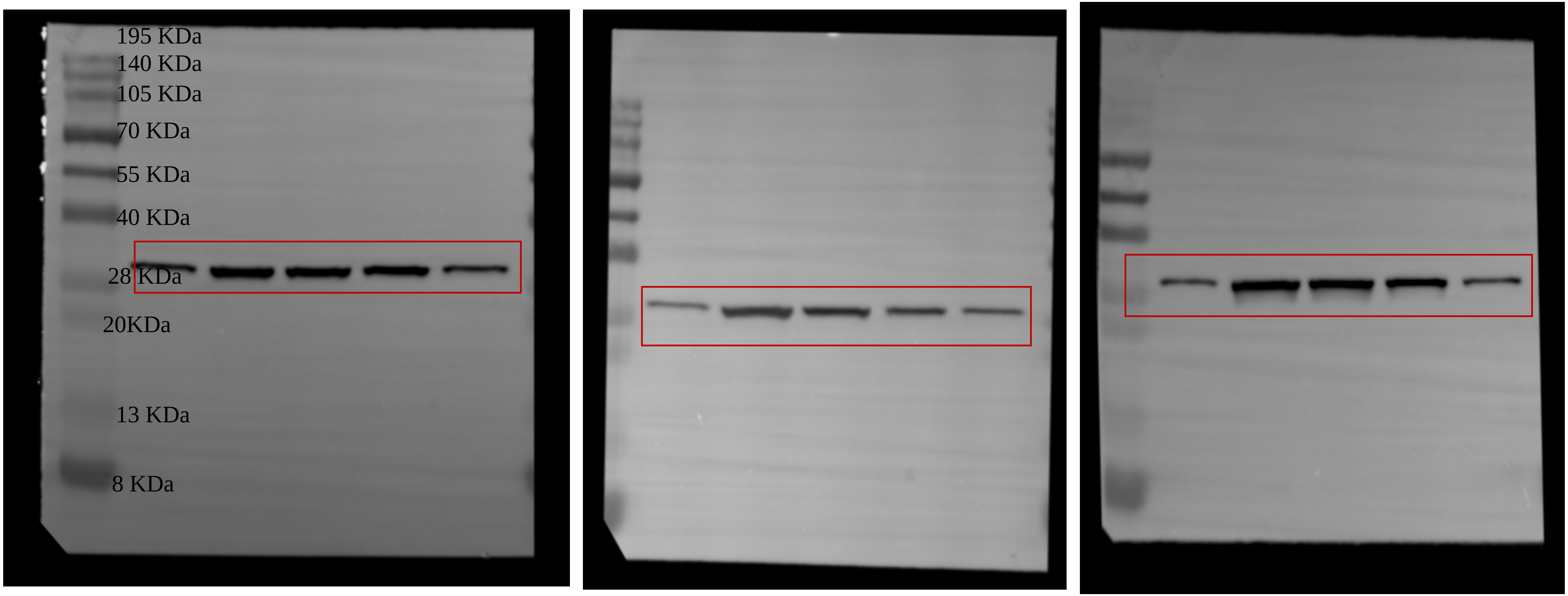

β-Actin in rats

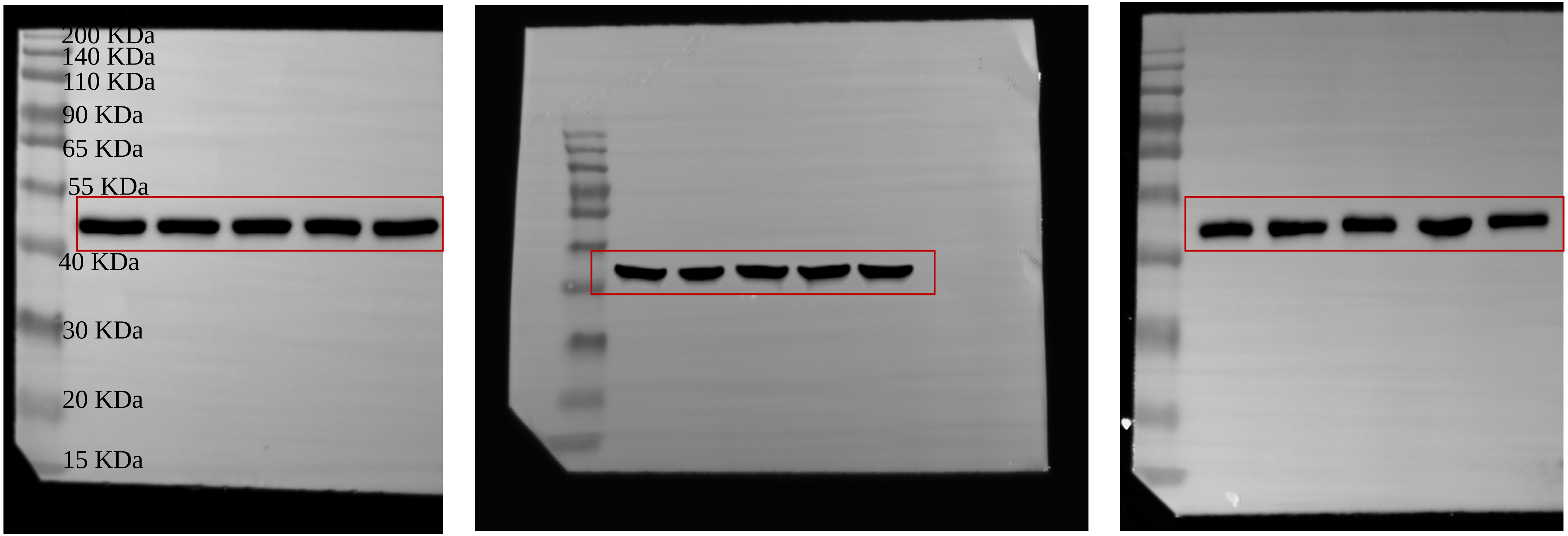

β-Actin in rats

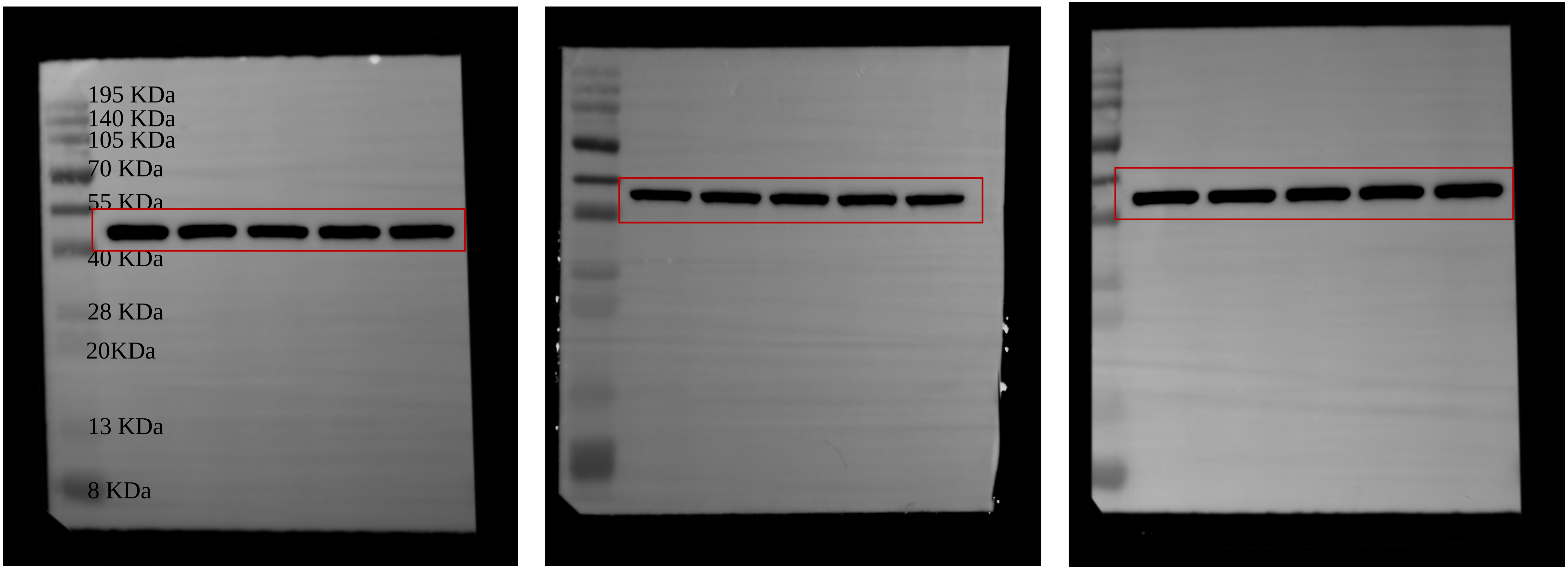

Supplement: Supplementary file 5 [file DataSheet2.ZIP › original data of figure 5/1. WB image of pyroptosis in rats.pdf]
