# Supplementary figures and images for: β-sitosterol alleviates dextran sulfate sodium-induced experimental colitis via inhibition of NLRP3/Caspase-1/GSDMD-mediated pyroptosis (part 2 of 2)
Source: Front Pharmacol. 2023 Oct 26;14:1218477. doi: 10.3389/fphar.2023.1218477 (PMC10637366; doi:10.3389/fphar.2023.1218477)

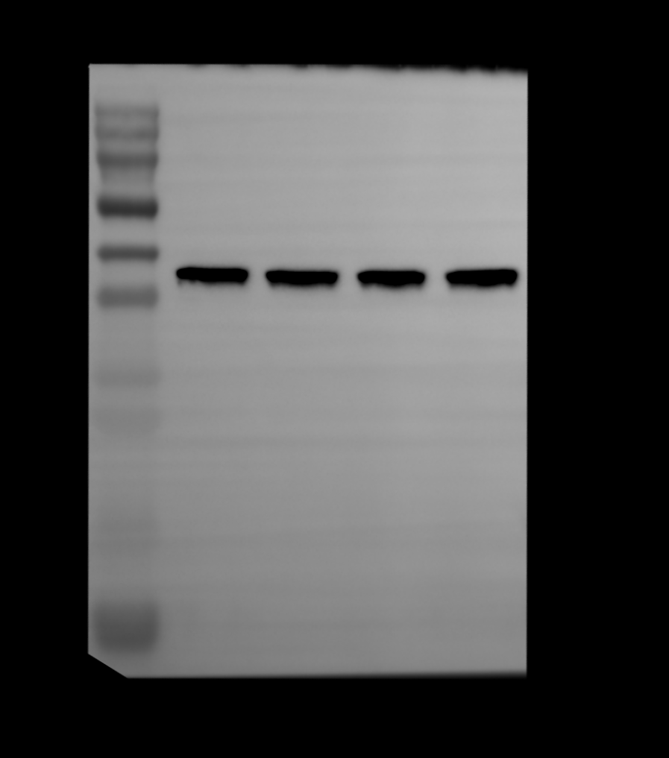

Supplement: Supplementary file 5 [file DataSheet2.ZIP › original data of figure 5/β-Actin in Caco2-4.tif]

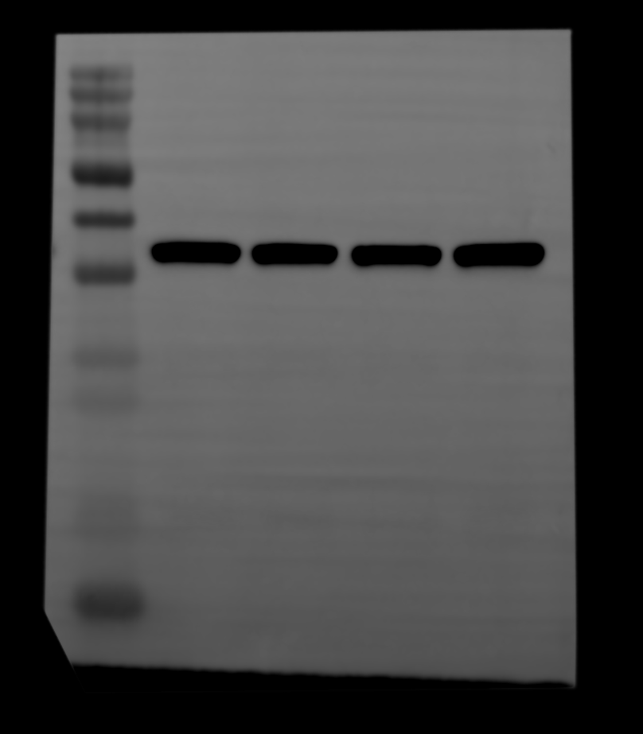

Supplement: Supplementary file 5 [file DataSheet2.ZIP › original data of figure 5/β-Actin in Caco2-5.tif]

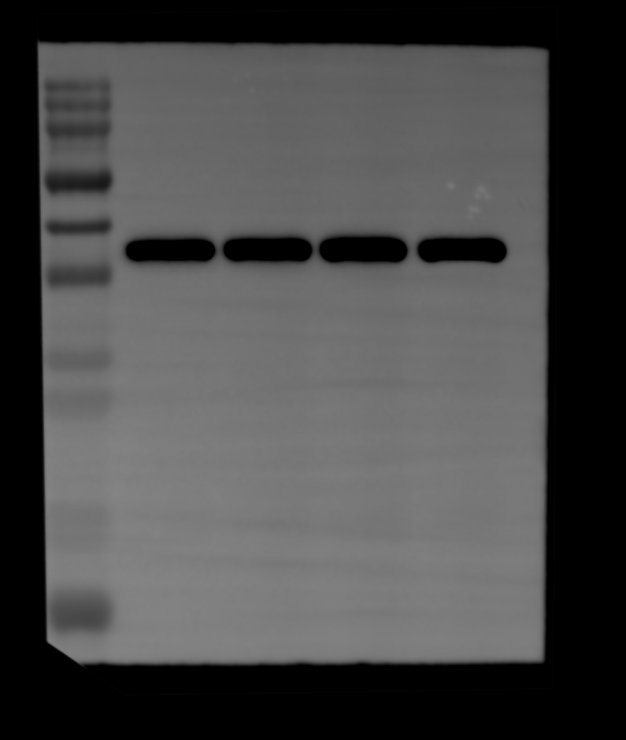

Supplement: Supplementary file 5 [file DataSheet2.ZIP › original data of figure 5/β-Actin in Caco2-6.tif]

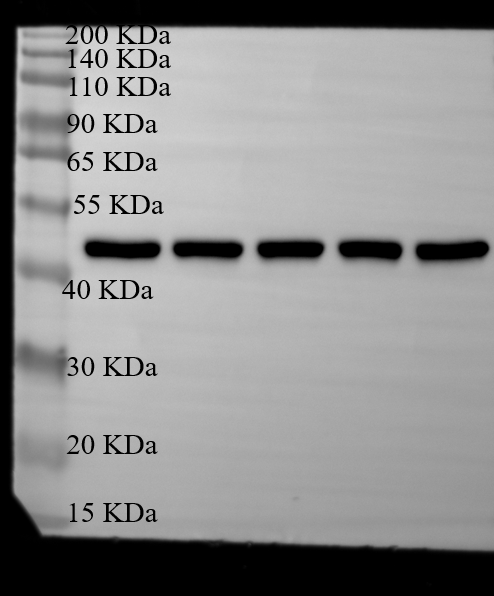

Supplement: Supplementary file 5 [file DataSheet2.ZIP › original data of figure 5/β-Actin in rats-1.tif]

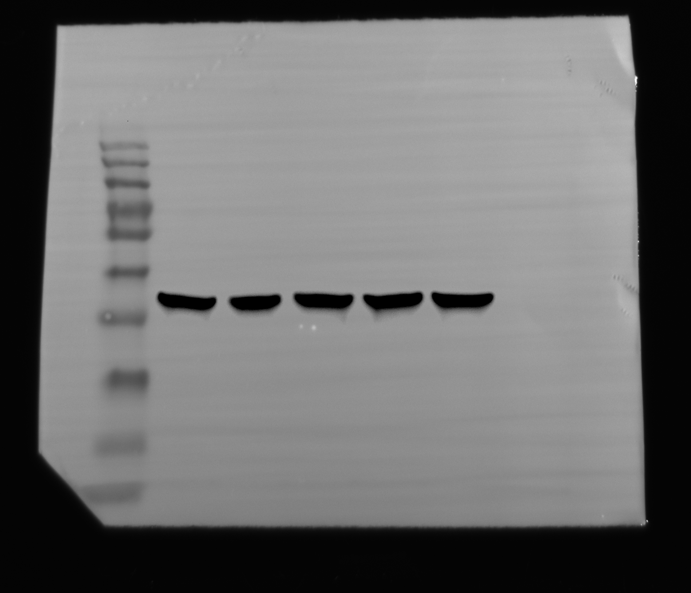

Supplement: Supplementary file 5 [file DataSheet2.ZIP › original data of figure 5/β-Actin in rats-2.tif]

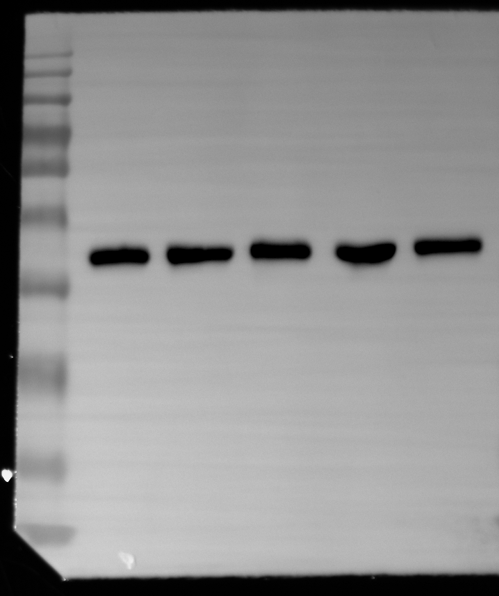

Supplement: Supplementary file 5 [file DataSheet2.ZIP › original data of figure 5/β-Actin in rats-3.tif]

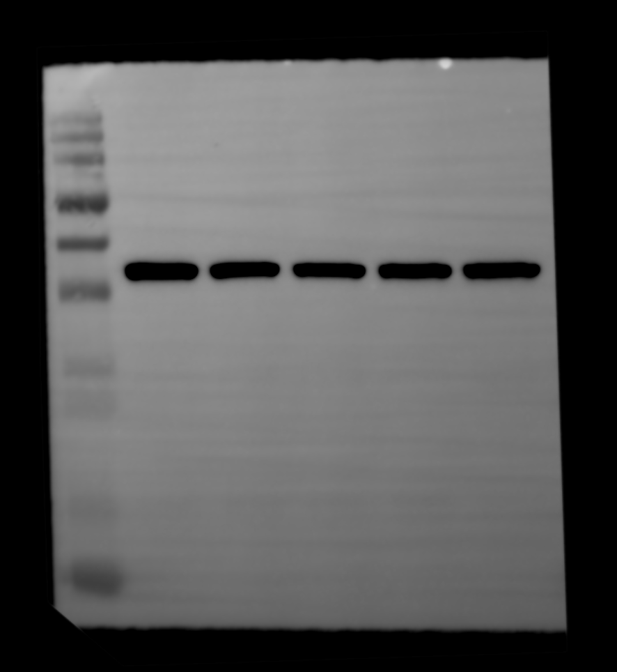

Supplement: Supplementary file 5 [file DataSheet2.ZIP › original data of figure 5/β-Actin in rats-4.tif]

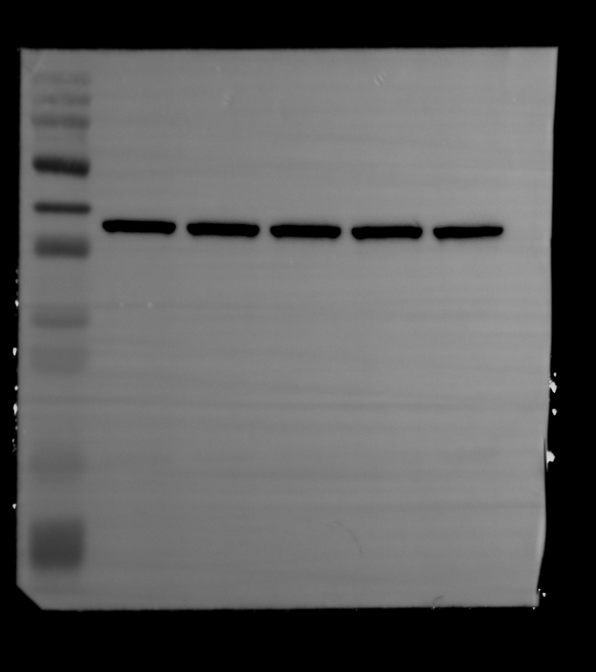

Supplement: Supplementary file 5 [file DataSheet2.ZIP › original data of figure 5/β-Actin in rats-5.tif]

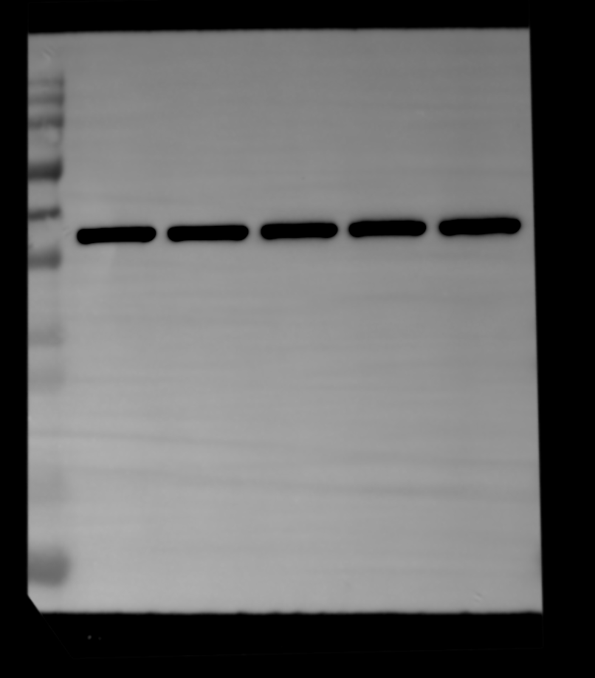

Supplement: Supplementary file 5 [file DataSheet2.ZIP › original data of figure 5/β-Actin in rats-6.tif]

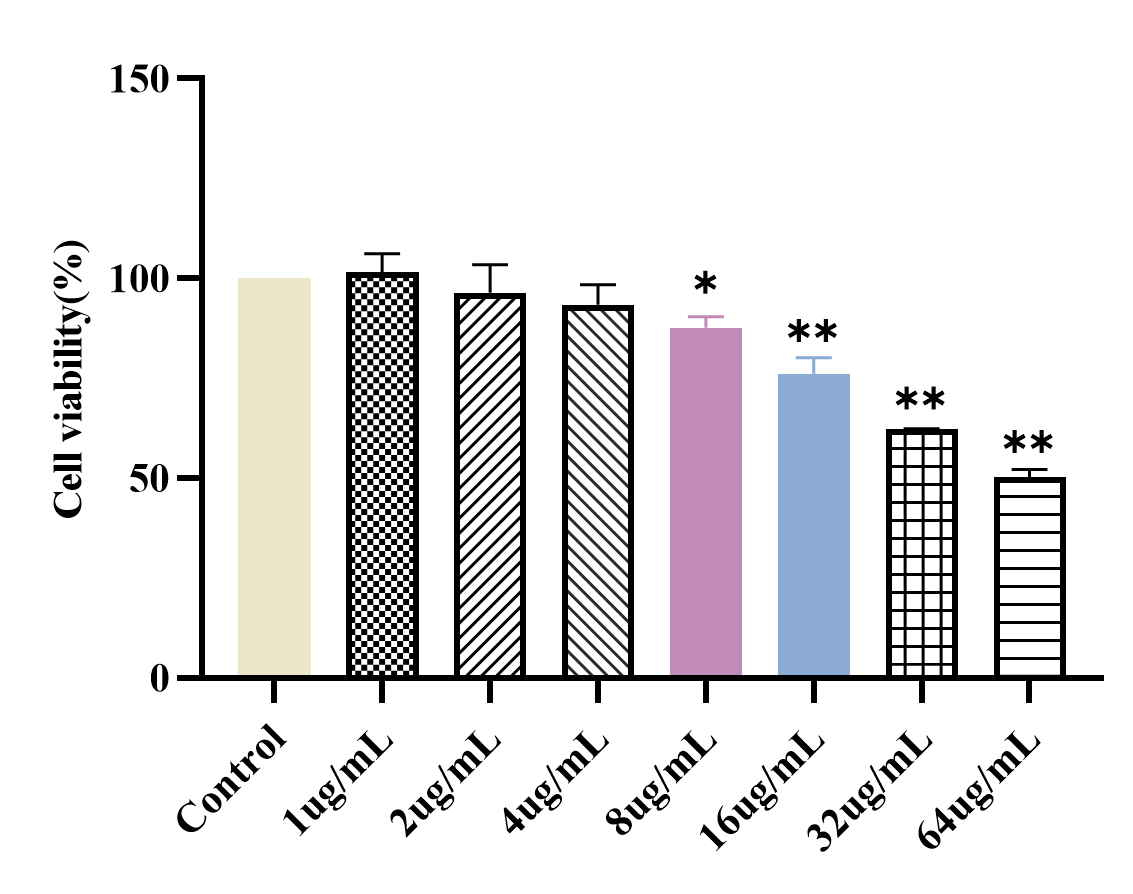

Supplement: Supplementary file 6 [file DataSheet5.ZIP › original data of figure 3/Figure1 SIT.tif]

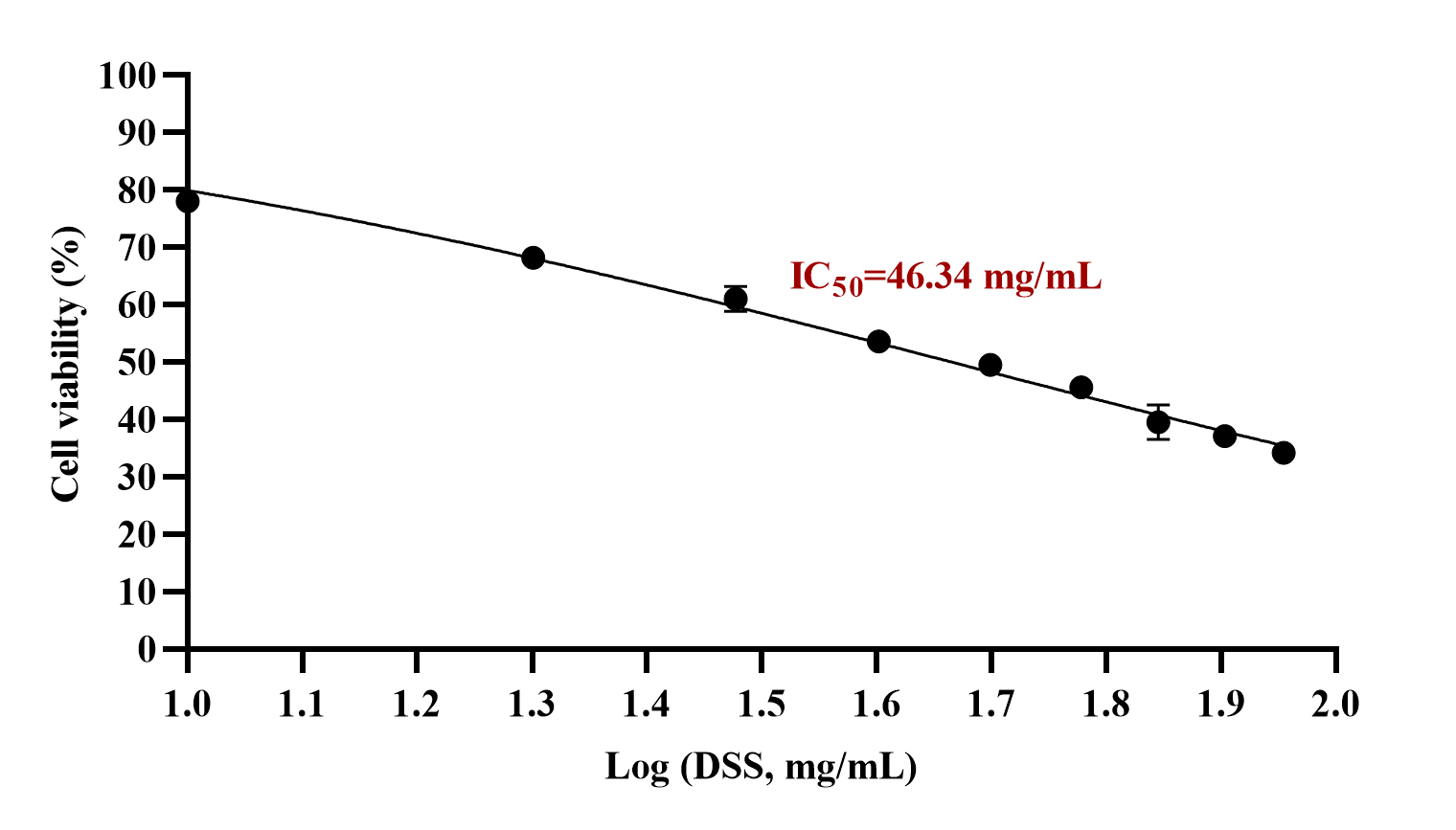

Supplement: Supplementary file 6 [file DataSheet5.ZIP › original data of figure 3/Figure2 Transform X of IC50.tif]

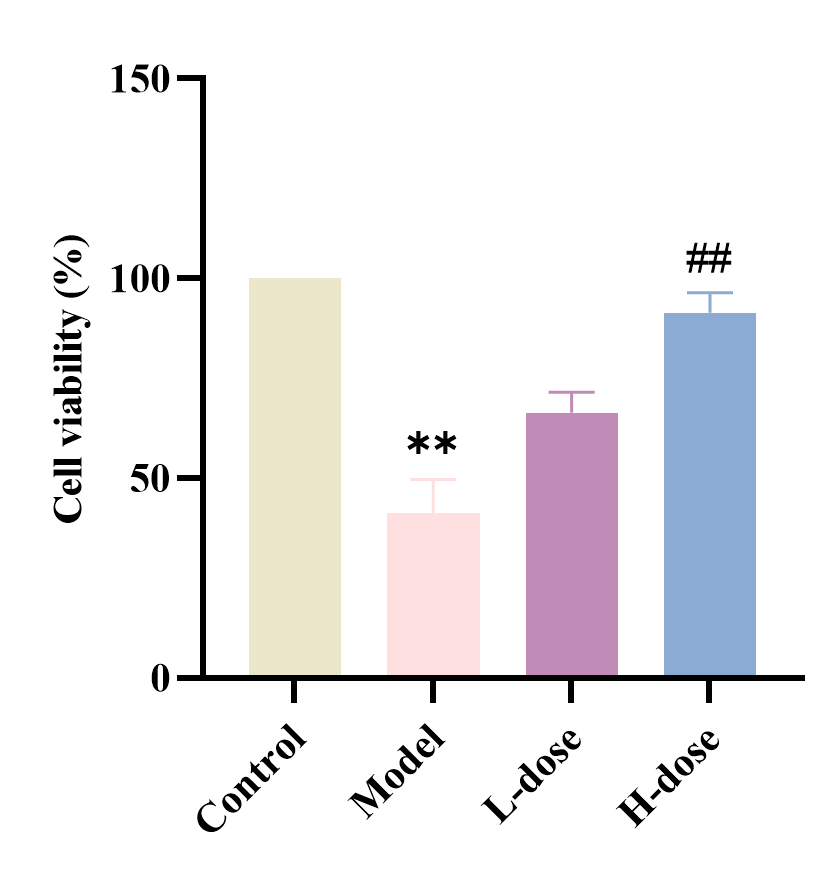

Supplement: Supplementary file 6 [file DataSheet5.ZIP › original data of figure 3/Figure3 SIT+DSS.tif]

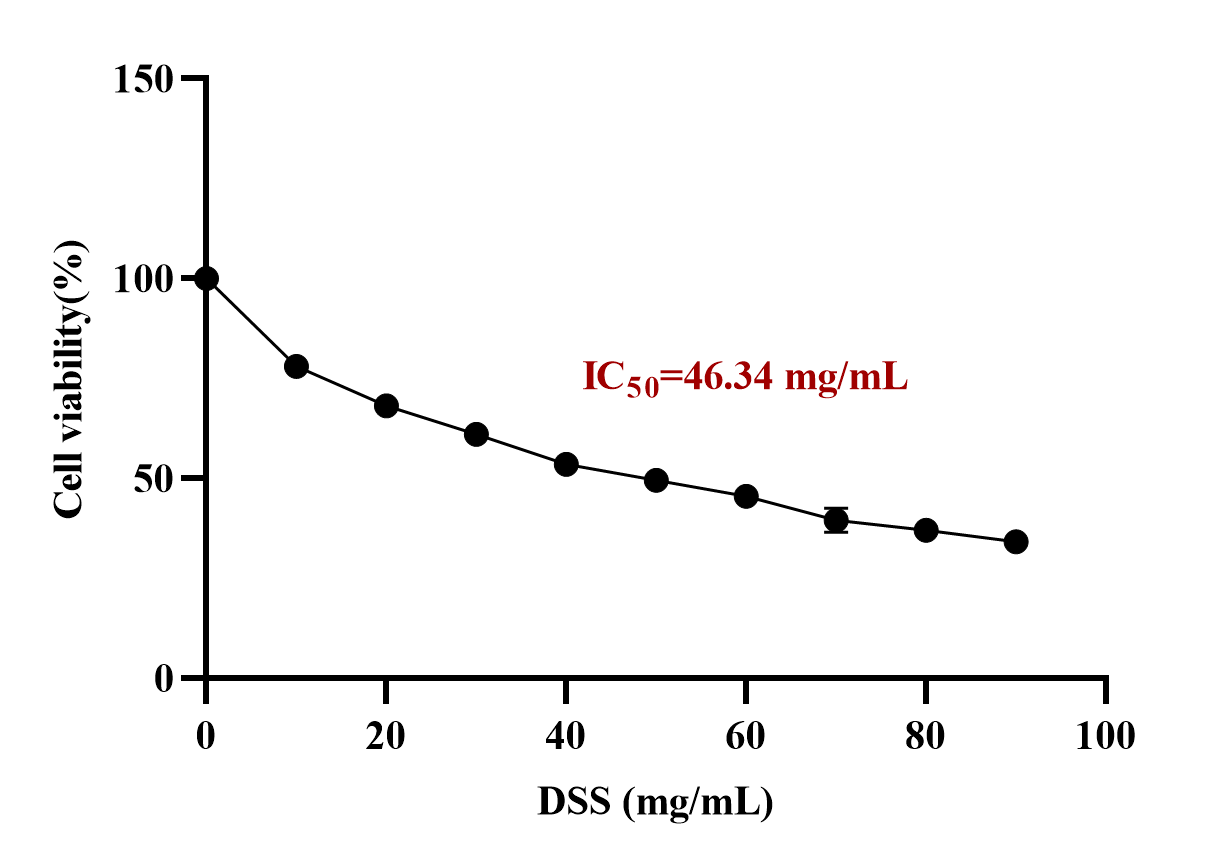

Supplement: Supplementary file 6 [file DataSheet5.ZIP › original data of figure 3/IC50.tif]
